# Increase of depression among children and adolescents after the onset of the COVID-19 pandemic in Europe: a systematic review and meta-analysis

**DOI:** 10.1186/s13034-022-00546-y

**Published:** 2022-12-31

**Authors:** Helena Ludwig-Walz, Indra Dannheim, Lisa M. Pfadenhauer, Jörg M. Fegert, Martin Bujard

**Affiliations:** 1 Federal Institute for Population Research (BiB), Wiesbaden, Germany; 2 Regional Innovative Centre of Health and Quality of Live Fulda (RIGL), Fulda University of Applied Sciences, Fulda, Germany; 3 Department of Nutritional, Food and Consumer Sciences, Fulda University of Applied Sciences, Fulda, Germany; 4 Institute for Medical Information Processing, Biometry and Epidemiology-IBE, Chair of Public Health and Health Services Research, LMU Munich, Munich, Germany; 5 Pettenkofer School of Public Health, Munich, Germany; 6 Department for Child and Adolescent Psychiatry and Psychotherapy, University Medical Center, Competence Domain Mental Health Prevention, Ulm, Germany; 7 Institute for Medical Psychology, Medical Faculty, University Heidelberg, Heidelberg, Germany

**Keywords:** Depression, COVID-19, Children, Adolescents, Europe, Systematic review, Meta-analysis

## Abstract

**Background:**

Research points to a high depression burden among youth during the COVID-19 pandemic; however, a lack of systematic evidence exists. We determine the change in depression symptoms among children and adolescents during COVID-19 compared to pre-pandemic baselines. By using country differences in pandemic-related restrictions and school closures in Europe as quasi-experimental design, we evaluate policy impacts on depression.

**Methods:**

In this systematic review and meta-analysis, following the PRISMA statement, we searched six databases (MEDLINE, EMBASE, PsycINFO, Cochrane Central, Web of Science, WHO COVID-19) using a peer-reviewed search string up until March 18, 2022 with citation tracking and grey literature searches. No limitations regarding language and effect measures existed. We included studies that compared (1) general depression symptoms or (2) clinically relevant depression rates in children and adolescents (≤ 19 years) before and during the COVID-19 pandemic in Europe. The validated Oxford Stringency Index was used as indicator for pandemic-related restrictions. Screening for eligibility, extracting data from published reports and from unpublished data requested directly from study authors, assessing the study risk of bias and grading certainty of evidence using the GRADE approach, were all done in duplicate. Data were pooled in a random-effects model. PROSPERO: CRD42022303714.

**Results:**

Of 7,422 nonduplicate records, 22 studies with data from 868,634 participants pre-pandemic and 807,480 during pandemic, met full inclusion criteria. For the comparison of depression symptoms before and during the COVID-19 pandemic, moderate certainty of evidence was observed for general depression symptoms (standardized mean difference, 0.21 [95%CI, 0.12–0.30]; I^2^ = 94%) and low certainty of evidence for clinically relevant depression rates (odds ratio, 1.36 [95%CI, 1.05–1.76]; I^2^ = 95%) for total population. Increase in general depression symptoms was higher for male adolescents, whereas increase in clinically relevant depression rates was higher for females. Effect estimates were significantly higher when pandemic-related restrictions were more stringent or school closure occurred.

**Conclusion:**

An increase in depression symptoms occurred in a pre-pandemic vs. during-pandemic comparison within the COVID-19 pandemic, whereby pandemic-related restrictions (such as school closures) resulted in a considerable effect increase. Ensuring adequate supply of mental health recovery services and long-term monitoring is of high public health relevance.

**Supplementary Information:**

The online version contains supplementary material available at 10.1186/s13034-022-00546-y.

## Background

Childhood and adolescence represent sensitive periods in development. Therefore, children and adolescents are particularly vulnerable to external influences and long-term health consequences - especially those related to mental health-can be perpetuated [1–3]. Even prior to the onset of the COVID-19 pandemic, depression and anxiety represented the greatest burden of disease among young people in Europe [2, 4], and depression was second in the top five causes of overall disease burden for youth in Europe [2], with more than 50% of these remaining into adulthood With the onset of the pandemic, European countries implemented a broad range of public health and social measures (PHSM) [5] with varying intensities to minimize infections. Mainly, PHSM focused on reductions of social contacts with major implications for the environment of children and adolescents, such as school and leisure facilities closing, decreased peer interactions, changes in the family system due to home office and quarantine orders [1, 6]. These PHSM have the potential to influence the depression distribution in youth significantly [1] and contribute to a widespread public health mental crisis in European youth [7].

Therefore, the ascertainment of pandemic-induced changes in depression distribution is of high public health relevance and was designated as a research priority [8]. The number of studies conducted in Europe is constantly increasing, although the studies differ in their quality, measurement instruments and effect direction; so far, no study makes use of quasi-experimental designs to assess the variation in PHSM among European countries and their effects on youths’ depression. Moreover, a high-quality synthesis focusing on studies in Europe is still lacking. Thus, the aim of this systematic review is to identify, critically appraise, synthesize and assess the certainty of evidence regarding the impact of the COVID-19 pandemic on depression among children and adolescents in Europe compared to a pre-pandemic baseline and evaluate the relevance of the stringency of the measures.

## Methods

### Search strategy and selection criteria

This systematic review and meta-analysis was registered on the International Prospective Register of Systematic Reviews (PROSPERO; CRD42022303714), a review protocol was published [9], and the review adheres to the Preferred Reporting Items for Systematic Reviews and Meta-analysis (PRISMA) statement [10, 11] (Additional file 1: Table S1).

We searched in six electronic databases (MEDLINE, EMBASE, PsycINFO, Cochrane Central, Web of Science, WHO COVID-19 [including pre-prints]) for published articles until March 18, 2022. In addition, we conducted forward citation tracking of all included studies, related systematic reviews and meta-analyses, as well as screened conference abstracts and websites of key organizations till April 16, 2022; more information is provided in Additional file 1: Table S2.

The search strategy combined search terms from three domains: (1) depression, (2) children and adolescents, and (3) COVID-19 (Additional file 1: Table S3). Database-specific search strings were developed using validated or recommended search filters [12–14]. The search strategy was peer reviewed according to the evidence-based checklist Peer Review of Electronic Search Strategies (PRESS) [15].

Following the Population–Exposure–Comparison–Outcome framework [16] we defined the following inclusion criteria: (1) healthy children and adolescents ≤ 19 years, living in the WHO European region [17]; (2) outcome measured during COVID-19 pandemic; (3) reporting of a plausible pre-pandemic baseline; and (4) measurement of general depression symptoms or clinically relevant depression rates [39, 47]. We excluded studies of children and adolescents with preexisting psychiatric diagnoses. No limits regarding language and effect measures were imposed. Multiple publications drawing upon the same study population and providing the same measurement points during the pandemic were considered as one study. Studies conducted with the same study population with varying pandemic measurement points were considered individually.

Study selection followed a three-stage process: (1) import and automated deduplication of identified studies to EPPI reviewer software [18]; (2) screening of titles and abstracts; (3) screening of full texts; screenings were performed independently by two reviewers (HLW, ID) in which disagreements or uncertainty about eligibility were resolved through discussion.

### Data analysis

Two authors (HLW, ID) independently extracted data using piloted extraction forms. The following data were extracted from published reports and unpublished data requested from study authors: study information (first author, year of publication, country, study type), population and setting (sample size [% female], age), COVID-19 determinants (time point [month/year] of data measurement), pre-pandemic baseline (time point [month/year] of data measurement, link between population before and during pandemic), outcomes (type of outcome, diagnostic instrument, psychometric properties of the diagnostic instrument, symptom reporter). The primary outcomes were general depression symptoms and clinically relevant depression rates. General depression symptoms were defined as measurements of depression symptoms. This outcome summarizes various instruments measuring depressive symptoms in children and adolescents (e.g. Child Behavior Checklist, Patient Health Questionnaire, Hopkins Symptom Checklist), with no specific clinical cut-off. The data were usually reported as continuous measurement, data that were only available as dichotomous variables for general depression symptoms were transformed according to the recommended formula by Chinn [19]. Because measurement instruments varied considerably, effect estimates were unified to standardized mean difference (SMD) with a 95% confidence interval (CI), also recommend by the Cochrane Handbook for depression as an outcome measure [10]. Medical classifications (International Statistical Classification of Diseases and Related Health Problems reports) and measurement instruments with a clinical cut-off were summarized to clinically relevant depression rates which defined major depression; they were presented as a dichotomous effect estimate using odds ratios with a 95% CI. To allow comparison of the two effect measures (SMD and OR), we converted the total effect for general depression symptoms into OR using the Hasselblad and Hedges’ method [10]. For all studies, we used the Oxford COVID-19 Stringency Index [6] and the School Closure Index [6] as indicators for the COVID-19 PHSM impact. An Oxford Stringency Index > 60 was classified as ‘full lockdown’, 20–60 as ‘moderate lockdown’ and < 20 as ‘light restrictions’. A School Closure Index ≥ 2 was classified as ‘partial/full school closure’; further information is provided in Additional file 1: Methods.

We assessed risk of bias independently by three reviewers (HLW, LMP, ID) using the risk of bias instrument for non-randomized studies of exposures [20]. This instrument consists of seven items; we slightly adapted the tool by removing item number four (‘Bias due to departures from intended exposures’) due to lack of applicability to the question at hand. Detailed assessment criteria operationalizing the remaining six criteria are described in Additional file 1: Table S4. The assessment in each risk of bias item was summarized to an overall judgement for the whole study as low, moderate, serious or critical [20].

For meta-analysis, we grouped studies according to risk of bias assessment, aggregating low/moderate (= low) risk of bias studies and serious/critical (= high) risk of bias studies separately and in total to address substantial methodological heterogeneity and potential confounding. Pooled effect of the low-risk-of-bias studies was considered for further interpretation.

If multiple pre-pandemic time points exist, we used data at the latest possible time point to calculate effect estimates. Different subgroups were analysed: gender (female/male), age (0–5, 6–10, 11–15, 16–19 years), country, Oxford COVID-19 Stringency Index (> 60/ ≤ 60) [6], School Closure Index (≥ 2/ < 2) [6] and time of measurement (spring/summer 2020, autumn 2020, winter 2020/spring2021, summer 2021, autumn 2021). Effect estimates from combined scores of depression and anxiety were rejected from meta-analysis. When both parent and self-rated data were provided [21], we used the self-rated data. We conducted meta-analysis calculations in Review Manager 5.4.1 [22] and R Studio 4.2.1 [23] using the inverse-variance random-effects model with the ‘DerSimonian and Laird’ approach [10].

Heterogeneity was assessed visually and using Chi^2^ test and I^2^ index [24]. We assumed substantial heterogeneity if I^2^ > 50%. To explain substantial heterogeneity, sensitivity analyses and meta-regression analyses (if ≥ 10 studies per examined variable) were performed [10]. We investigated publication bias by visually interpreting funnel plots for signs of asymmetry [10] and statistically by calculating the Eggers’ test (if ≥ 10 studies) [25].

Certainty of evidence for each body of evidence was evaluated by using the Grading of Recommendations Assessment, Development and Evaluation (GRADE) approach [26]. Five domains for downgrading and three domains for upgrading certainty of evidence are considered in GRADE; applied criteria are listed in Additional file 1: Table S5. GRADE finally specifies four levels of certainty of evidence for a body of evidence for each outcome: high, moderate, low and very low [26].

## Results

Electronic search retrieved 7420 nonduplicate records and 2 grey literature publications. A total of 51 full-text articles were retrieved, and 22 studies [21, 27–47] with 26 effect estimates met full inclusion criteria (Additional file 1: Fig. S1; exclusion reasons after full-text screening are described in Additional file 1: Table S6).

### Study characteristics

Study characteristics are presented in the Table 1. Of the 22 studies, five were from Germany [29, 38, 41, 42, 46], four from Norway [30, 34, 40, 47] and the United Kingdom [21, 27, 37, 45], two from Italy [31, 33], Iceland [35, 44], Netherlands [36, 39], Switzerland [28, 32] and one from Israel [43]. The majority were conducted in spring/summer 2020 (17 effects), followed by winter 2020/spring 2021 (five effects) and autumn 2020 (four effects). From the included studies, 21 (95%) [21, 27–32, 34–47] provided data for children and adolescents over 11 years and seven (32%) for children and adolescents under 11 years [28, 31, 33, 38, 39, 41, 46]. In total, data were included from 868,634 participants pre-pandemic and 807,480 participants during pandemic. Outcome measures differentiated between general depression symptoms (63,744 pre-pandemic and 116,858 during pandemic) and clinically relevant depression rates (743,736 pre-pandemic and 751,776 during pandemic). In 15 studies, measurement time point was classified as ‘full lockdown’ (Oxford COVID-19 Stringency Index > 60) [21, 27–29, 31, 33, 36–39, 41–43, 45, 47], and in 11 studies, schools were partially or fully closed (School Closure Index ≥ 2) [21, 27, 29, 31, 33, 36, 37, 39, 42, 43, 45]. Additional unpublished data were provided by 16 studies [21, 27, 29–33, 36, 38–42, 45–47] (in particular, gender and age-stratified data). All effect estimates of the included studies are summarized in Additional file 1: Table S7. Risk of bias was moderate in 11 studies [29, 30, 32, 38–40, 42–44, 46, 47], serious in nine [21, 27, 33–37, 41, 45] and critical in two [28, 31]; detailed information is provided in Additional file 1: Table S8 and in Additional file 1: Fig. S2 (traffic light plots) and Additional file 1: Fig. S3 (weighted bar plots).

**Table 1:** Characteristics of included studies

Study information		Population		Exposure		Comparison		Outcome		Risk of bias
First author, year	Study type, name of the study	Sample size (% female)	Age of study population	Time point during COVID-19 pandemic	Policy indices [6], mean (min to max)	Time point of pre-pandemic baseline	Link between pre-pandemic and during pandemic population	Type of outcome	Diagnostic instrument, symptom reporter	
**Germany**										
Ravens-Sieberer, 2022 [42]	Cohort study, German COPSY study	PP: 994 (47.8) DP1: 1,018 (49.6) DP2: 1,073 (49.2) DP3: 1,173 (48.4)	PP: NI DP1: Mean (years) ± SD, 12.3 ± 3.3 DP2: Mean (years) ± SD, 12.7 ± 3.3 DP3: Mean (years) ± SD, 14.8 ± 2.3 Age range (years): 11 to 19	DP1: 5–6/2020 DP2: 12/2020–1/2021 DP3: 9–10/2021	DP1 *Stringency index:* 59.7 (57.4 to 59.7) *School closure index:* 2.0 (2.0 to 2.0) DP2 *Stringency index:* 83.1 (82.4 to 85.2) *School closure index:* 3.0 (3.0 to 3.0) DP3 *Stringency index:* 49.2 (37.0 to 60.2) *School closure index:* 1.3 (1.0 to 3.0)	2017 (nationwide, longitudinal, representative BELLA study)	Both study samples (BELLA and COPSY) are representative samples of German children and adolescents	General depression symptoms	Center for Epidemiological Studies Depression Scale (CES-DC), self-reported	Moderate
Witte, 2022 [46]	Cross-sectional study, medical record data from a health insurance company	PP: 533,701 (48.6) PP: 332,945 (48.6; stationary care) DP1: 545,626 (48.6) DP1: 339,361 (48.6; stationary care) DP2: 343,642 (48.5; stationary care)	Age range (years): 5 to 17	DP1: 2020 DP2: 2021	DP1: *Stringency index:* 51.8 (0 to 76.9) *School closure index:* 1.6 (0.0 to 3.0) DP2: *Stringency index:* 67.0 (46.3 to 85.2) *School closure index:* 1.9 (1.0 to 3.0)	2019	Cross-sectional population samples	Clinically relevant depression rates	Pediatric visit/ Hospitalization rate ICD-10: F32/33, pediatric reported	Moderate
Bujard, 2021 [29]	Cohort study, pairfam	PP: 854 (57.7) DP: 854 (57.7)	Age range (years): 16 to 19	5–7/2020	*Stringency index:* 60.4 (55.1 to 63.4) *School closure index:* 2.2 (1.0 to 3.0)	11/2018 to 7/2019	Same population	General depression symptoms, Clinically relevant depression rates	German adaption of State-Trait Depression Scale (STDS), self-reported	Moderate
Kostev, 2021 [38]	Cross-sectional study, medical record data from the Disease Analyzer database (IQVIA)	PP: 206,528 (39.2) DP: 203,742 (39.7)	PP: Mean (years) ± SD, 6.6 ± 4.9 DP: Mean (years) ± SD, 6.7 ± 5.0 Age range (years): 2 to 17	4/2020 to 12/2020	*Stringency index:* 62.2 (49.5 to 82.4) *School closure index:* 1.8 (1.0 to 3.0)	4/2019 to 12/2019	Cross-sectional population samples	Clinically relevant depression rates	Pediatric visit ICD-10: F32, F33, pediatric reported	Moderate
Rau, 2021 [41]	Cohort study	PP: 777 (53.3) DP: 777 (53.3)	Mean (years) ± SD, 12.9 ± 2.0 Age range (years): 9 to 17	6–7/2020	*Stringency index:* 59.7 (57.4 to 63.4) *School closure index:* 1.9 (1.0 to 3.0)	PP1: 10–11/2019 PP2: 1–2/2020	Same population	General depression symptoms, Clinically relevant depression rates	Revised Child Anxiety and Depression Scale (RCADS), depression subscale, self-reported	Serious
**Iceland**										
Thorisdottir, 2021 [44]	Longitudinal study, Youth in Iceland school surveys	PP1: 19,682 (50.1) PP2: 18,126 (50.4) DP: 15,725 (51.8)	Age range (years): 13 to 18	10/2020	*Stringency index:* 43.1 (38.0 to 52.8) *School closure index:* 1.0 (1.0 to 1.0)	PP1: 2016 PP2: 2018	Same population	General depression symptoms	Symptom Checklist‐Revised (SCL‐90), subscales of depressed mood and anger, self-reported	Moderate
Halldors-dottir, 2021 [35]	Cross-sectional study, LIFECOURSE (Longitudinal Investigation For Epidemiologic Causes and OUtcomes RiSing in Early Childhood and Adolescence)	PP: 504 (43.1) DP: 504 (43.1)	Age range (years): 16 to 17	10/2020–4/2021	*Stringency index:* 49.0 (40.7 to 65.7) *School closure index:* 1.2 (1.0 to 3.0)	2018	Cross-sectional measure	General depression symptoms	Symptom Checklist‐Revised (SCL‐90), subscales of depressed mood and anger, self-reported	Serious
**Israel**										
Shoshani, 2021 [43]	2-point survey	PP: 1537 (52) DP: 1,537 (52)	Mean (years) ± SD, 14.0 ± 2.0 Age range (years): 11 to 17	4/2020	*Stringency index:* 77.3 (75.0 to 84.3) *School closure index:* 2.1 (2.0 to 3.0)	9/2019	Same population	General depression symptoms	Brief Symptom Inventory 18 (BSI-18), subscale depression, self-reported	Moderate
**Italy**										
Frigerio, 2022 [33]	Longitudinal study, Effect of Depression on Infants (EDI)	PP1: 94 (46.8) PP2: 88 (46.6) DP: 59 (45.8)	PP1: Mean (months) ± SD, 13.7 ± 1.63 PP2: Mean (years) ± SD, 3.5 ± 0.3 DP: Mean (years) ± SD, 4.2 ± 0.6	4–6/2020	*Stringency index:* 77.4 (67.6 to 93.5) *School closure index:* 3.0 (3.0 to 3.0)	PP measures 1 and 2 (no detailed information)	Same population	General symptoms, subscale anxious/ depressed	Child Behavior Checklist (CBCL 1½-5), subscale anxious/depressed, parent-reported	Serious
Crescentini, 2020 [31]	Online survey	PP: 721 (48.4) DP: 721 (48.4)	Mean (years) ± SD, 10.1 ± 2.5 Age range (years): 6 to18	4–5/2020	*Stringency index:* 90.2 (75.0 to 93.5) *School closure index:* 3.0 (3.0 to 3.0)	Backward consideration (the last months of 2019)	Same population	General depression symptoms	Child Behavior Checklist (CBCL 6–18), subscale withdrawn/depressed, parent-reported	Critical
**Netherlands**										
Luijten, 2021 [39]	Cross-sectional study	PP: 1,318 (50.1) DP: 813 (54.6)	PP: Mean (years) ± SD, 13.1 ± 3.1 DP: Mean (years) ± SD, 13.4 ± 2.8 Age range (years): 8 to 18	4–5/2020	*Stringency index:* 78.7 (78.7 to 78.7) *School closure index:* 3.0 (3.0 to 3.0)	12/2017–7/2018 (2 studies)	PP: 2 representative studies DP: 1 representative study (not the same population)	General depression symptoms	Patient-Reported Outcome Measurement Information System (PROMIS), CAT V2.0-Depressive Symptoms, self-reported	Moderate
Janssen, 2020 [36]	Cohort study, RE-PAIR study: ‘Relations and Emotions in Parent-Adolescent Interaction Research’ and on the follow-up assessment ‘RE-PAIR during the COVID-19 pandemic	PP: 28 (64.3) DP: 20 (65.0)	PP: Mean (years) ± SD, 16 ± 1.2 DP: Mean (years) ± SD, 17.0 ± 1.0 Age range (years): 11 to 17	4/2020	*Stringency index:* 78.7 (78.7 to 78.7) *School closure index:* 3.0 (3.0 to 3.0)	9/2018- 11/2019	Same population	General depression symptoms	Patient Health Questionnaire (PHQ-9), self-reported	Serious
**Norway**										
Soest, 2022 [47]	Cross-sectional survey, Norwegian national youth survey (Ungdata)	PP1: 11,719 (49) PP2: 24,694 (50) PP3: 10,555 (50) PP4: 44,103 (51) PP5: 30,246 (50) PP6: 8,792 (50) PP7: 10,552 (50) DP: 86,597 (51)	PP1: Mean (years) ± SD, 15.1 ± 1.6 PP2: Mean (years) ± SD, 15.1 ± 1.5 PP3: Mean (years) ± SD, 15.0 ± 1.5 PP4: Mean (years) ± SD, 15.3 ± 1.6 PP5: Mean (years) ± SD, 15.5 ± 1.6 PP6: Mean (years) ± SD, 15.5 ± 1.6 PP7: Mean (years) ± SD, 15.7 ± 1.6 DP: Mean (years) ± SD, 15.3 ± 1.6 total: Mean (years) ± SD, 15.3 ± 1.6 Age range (years): 13 to 18	1–3/2021	*Stringency index:* 68.9 (56.0 to 73.2) *School closure index:* 1.7 (1.0 to 2.0)	PP1: 2014 PP2: 2015 PP3: 2016 PP4: 2017 PP5: 2018 PP6: 2019 PP7: 2020 (before 3/2020)	Cross-sectional survey	General depression symptoms	Hopkins Symptom Checklist (HSCL), self-reported	Moderate
Burdzovic, 2021 [30]	Cohort study, MyLife study	PP: 1,335 (60.0) DP: 741 (59.8)	School grades 10 and 11 (approx. 15 to 16 years)	10–12/2020	*Stringency index:* 48.1 (32.4 to 56.0) *School closure index:* 1.0 (1.0 to 1.0)	2018/2019	Same population	General depression symptoms, Clinically relevant depression rates	Patient Health Questionnaire (PHQ-9; adolescent version), self-reported	Moderate
Myhr, 2021 [40]	Cross-sectional survey, subsample of the Norwegian national youth survey (Ungdata) in Trøndelag County	PP: 2,126 (52.0) DP: 1,957 (50.7)	Age range (years): 13 to 16	5/2020	*Stringency index:* 58.3 (58.3 to 58.3) *School closure index:* 1.0 (1.0 to 1.0)	3/2020	PP: Subsample of a representative survey DP: cross-sectional data	General depression symptoms	Hopkins Symptom Checklist (HSCL), self-reported	Moderate
Hafstad, 2021 [34]	Representative longitudinal survey	PP: 3,572 (50.1) DP: 3,572 (50.1)	Mean (years) ± SD, 14.7 ± 4.1 Age range (years): 12 to 16	6/2020	*Stringency index:* 42.5 (40.7 to 58.3) *School closure index:* 1.0 (1.0 to 1.0)	2/2019	Same population	General depression symptoms	Hopkins Symptom Checklist (HSCL-10), self-reported	Serious
**Switzerland**										
Ertanir, 2021 [32]	Longitudinal study, ‘Overcoming Inequalities with Education’ project	PP: 359 (46.2) DP: 314 (43.0)	Mean (years) ± SD, 12.7 ± 0.7 Age range (years): 11 to 15	8–9/2020	*Stringency index:* 43.1 (43.1 to 43.1) *School closure index:* 0.0 (0.0 to 0.0)	9–10/2019	Same population	General depression symptoms	Hopkins Symptoms Checklist (HSCL-25), subscale depression, self-reported	Moderate
Borbás, 2021 [28]	Cohort study	PP: 26 (38.5) DP: 26 (38.5)	Mean (years) ± SD, 10.7 ± 2.5 Age range (years): 7 to 17	5/2020	*Stringency index:* 69.4 (69.4 to 69.4) *School closure index:* 0.0 (0.0 to 0.0)	3/2018 to 2/2020	Same population	General symptoms, subscale anxious/ depressed	Child Behavior Checklist (CBCL 6–18), subscale anxious/depressed, NI	Critical
**United Kingdom**										
Knowles, 2022 [37]	Cohort study, REACH (Resilience, Ethnicity, and AdolesCent Mental Health)	PP1: 955 (NI) PP2: 943 (NI) PP3: 836 (53.7) DP: 1,069 (54.5)	Age range (years): 12 to 18	5–8/2020	*Stringency index:* 71.1 (31.5 to 79.6) *School closure index:* 2.9 (2.0 to 3.0)	PP1: 2016–17 PP2: 2017–18 PP3: 2018–19	Same population	General depression symptoms	Short Mood and Feelings Questionnaire (SFMQ), depression as SMFQ score ≥ 12, self-reported	Serious
Widnall, 2022 [45]	Longitudinal 3-wave panel survey	PP: 589 (59.2) DP1: 587 (58.8) DP2: 587 (59.8)	Mean (years): 13.2 Age range (years): 13 to 15	DP1: 5/2020 DP2: 10/2020	DP1: *Stringency index:* 74.2 (69.4 to 79.6) *School closure index:* 3.0 (3.0 to 3.0) DP2: *Stringency index:* 67.9 (60.2 to 75.0) *School closure index:* 3.0 (3.0 to 3.0)	10/2019	PP and DP1: Same population DP2: Other population	General depression symptoms	Hospital Anxiety & Depression Scale (HADS), self-reported	Serious
Wright, 2021 [21]	Cohort study, population‐based birth cohort (Wirral Child Health and Development Study)	Self-rated: PP: 187 (46.5) DP: 163 (45.4) Mother-rated: PP: 226 (45.5) DP: 199 (54.8)	Mean (years) ± SD, 12.0 ± 0.4 Age range (years): 10 to 12	6–8/2020	*Stringency index:* 67.9 (64.4 to 73.2) *School closure index:* 2.8 (2.0 to 3.0)	12/2019-3/2020	Same population	General depression symptoms	Short Mood and Feelings Questionnaire (SMFQ), self-reported and mother-reported	Serious
Bignardi, 2020 [27]	Cohort study, Resilience in Education and Development (RED) study	School group: 114 (49.1) Lab group: 54 (63.0)	PP: School group: mean (years) ± SD, 8.7 ± 0.6 Lab group: mean (years) ± SD, 8.5 ± 0.7 DP: School group: mean (years) ± SD, 10.5 ± 0.7 Lab group: mean (years) ± SD, 9.4 ± 0.8 Age range (years): 7 to 11	4–6/2020	*Stringency index:* 74.9 (67.6 to 79.6) *School closure index:* 3.0 (3.0 to 3.0)	School group: 6/2018 to 3/2019 Laboratory group: 12/2018 to 9/2019	Same population	General depression symptoms	Revised Child Anxiety and Depression Scale (RCADS)-short form with anxiety and depression subscales, PP: self/parent-reported, DP: parent-reported	Serious

*DP* during pandemic, *M* mean, *NI* no information, *PP* pre-pandemic, *SD* standard deviation

### Meta-analysis of general depression symptoms

**Fig. 1:**
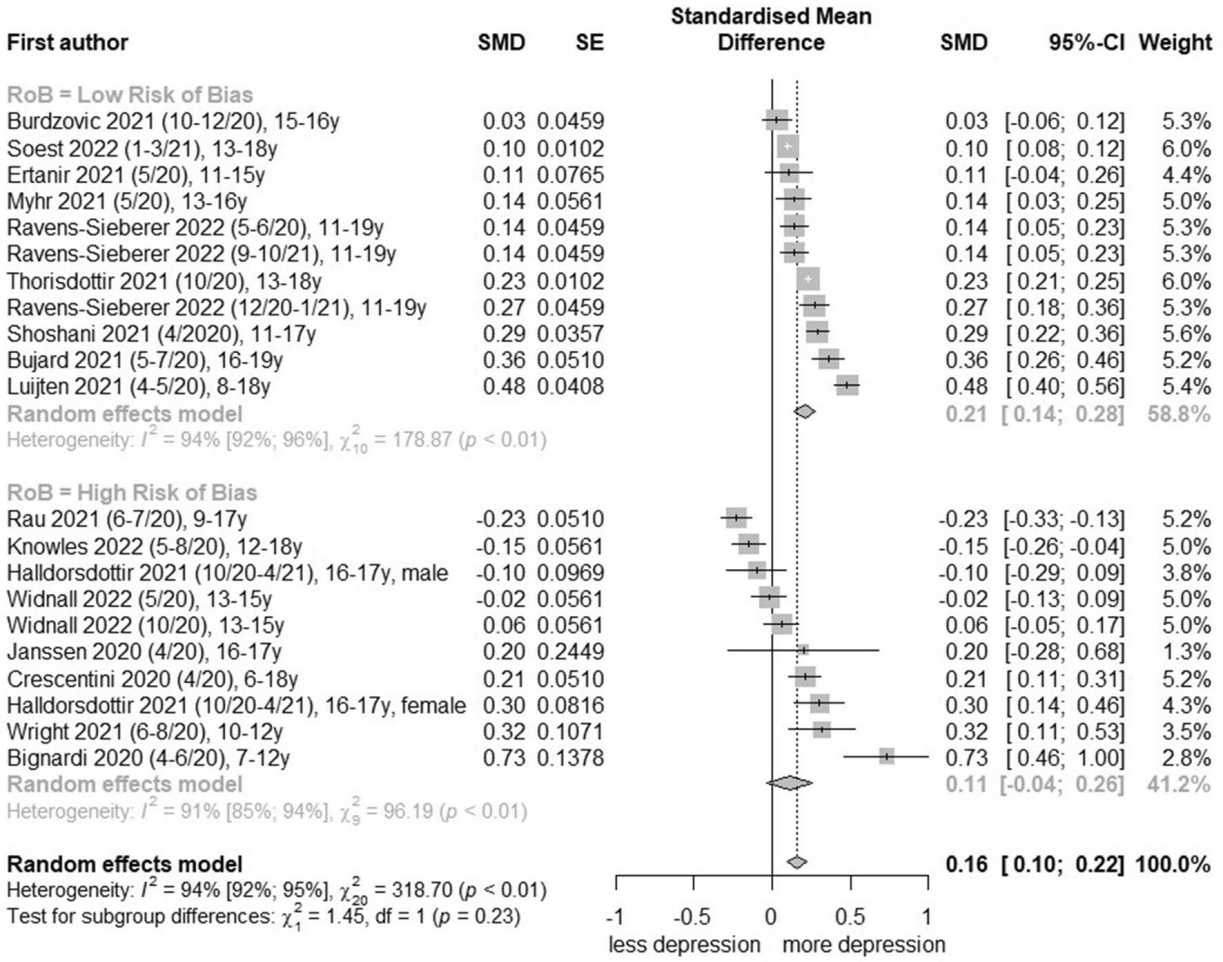
Forest plot of changes in youth general depression symptoms comparing before and during COVID-19 pandemic

For general depression symptoms, 17 studies [21, 27, 29–32, 35–37, 39–45, 47] were pooled and certainty of evidence was graded as ‘moderate’ (Table 2; further information in Additional file 1: Table S9). Total change effects of general depression symptoms before and during the COVID-19 pandemic revealed in statistical pooling of nine low-risk-of-bias studies [29, 30, 32, 39, 40, 42–44, 47] with 11 effect estimates a SMD of 0.21 (95% CI, 0.12 to 0.30, I^2^ = 94%; Fig. 1) respectively a converted OR of 1.46 (95% CI, 1.24 to 1.72; Table 2). Comparisons of gender-stratified analysis in seven low-risk-of-bias studies [29, 30, 32, 39, 40, 42, 47] with nine measurements yielded an increase for both females (SMD, 0.17 [95% CI, 0.04 to 0.31, I^2^ = 88%]; Additional file 1: Fig. S4) and males (SMD, 0.22 [95% CI, 0.06 to 0.37, I^2^ = 93%]; Additional file 1: Fig. S5). An age-stratified comparison of effect changes for general depression symptoms before and during the COVID-19 pandemic with low-risk-of-bias studies was possible only for the age categories 11–15 years and 16–19 years. The age category 11–15 years included six low-risk-of-bias studies [32, 39, 40, 42, 44, 47] with 8 effect estimates and yielded for the total population a SMD of 0.21 (95% CI, 0.16 to 0.27, I^2^ = 73%; Additional file 1: Fig. S6), effect estimates were also significant for both female and male children and adolescents in low-risk-of-bias studies (Additional file 1: Figs. S7 and S8). The age category 16–19 years contained six low-risk-of-bias studies [29, 30, 39, 42, 44, 47] with eight effect estimates and yielded a SMD of 0.17 (95% CI, 0.07 to 0.27, I^2^ = 96%; Additional file 1: Fig. S9) for the total population; also, effect estimate for males was significant (SMD, 0.27 [95% CI, 0.13 to 0.41], I^2^ = 86%; Additional file 1: Fig. S10), but not for females (Additional file 1: Fig. S11). The consideration of different pandemic restriction levels in the meta-analysis of nine low-risk-of-bias studies [29, 30, 32, 39, 40, 42–44, 47] showed for every restriction level (Oxford COVID-19 Stringency Index > 60 vs ≤ 60, School Closure Index ≥ 2 vs < 2) a significant increase (Fig. 3). A ‘full lockdown’ (Oxford COVID-19 Stringency Index > 60: SMD, 0.30 (95% CI, 0.12 to 0.47, I^2^ = 97%; Fig. 3) and ‘partial/full closed schools’ (School Closure Index ≥ 2 SMD, 0.31 (95% CI, 0.15 to 0.46, I^2^ = 88%; Fig. 3) resulted in higher effect estimates for total population and also for female and male children and adolescents. Thereby, effect estimates for male children and adolescents were higher (data not shown). An analysis of effects by time of occurrence showed after a strong increase at the beginning of the COVID-19 pandemic (spring/summer 2020) a flattening over time (Additional file 1: Fig. S12). In effect pooling for countries, the highest effect estimate was determined for the Netherlands (SMD, 0.44 [95% CI, 0.26 to 0.63], I^2^ = 21%; Additional file 1: Fig. S13) and the lowest for Norway (SMD, 0.09 [95% CI, 0.05 to 0.13], I^2^ = 28%; Additional file 1: Fig. S13). Pooling-effect estimates of Germany, Iceland and United Kingdom were not significant.

**Table 2:** Summary of findings

Outcome	Number of studies	Standardized mean difference, 95% CI	Odds Ratio, 95% CI	Summary of findings	Certainty of evidence (GRADE)
General depression symptoms	17 studies [21, 27, 29–32, 35–37, 39–45, 47]	Low-risk-of-bias studies: 0.21, 0.12 to 0.30 All studies: 0.16, 0.07 to 0.25	Low-risk-of-bias studies: 1.46, 1.24 to 1.72 (converted^a^) All studies: 1.34, 1.14 to 1.57 (converted^a^)	Low risk of bias studies predicted an increase of general depression symptoms in the total population, female and male children and adolescents with a dose response-relationship	⊕ ⊕ ⊕ ⊝ Moderate^b,c^^,d^
Clinically relevant depressive rates	5 studies [29, 30, 38, 41, 46]		Low-risk-of-bias studies: 1.36, 1.05 to 1.76 All studies: 1.19, 0.93 to 1.53	Low risk of bias studies predicted an increase of clinically relevant depressive symptoms in the total population and female children and adolescents; however, with partly moderate confidence intervals	⊕ ⊕ ⊝ ⊝ Low^c,e^

^a^Conversion in odds ratio with 95% confidence interval according to the Hasselblad and Hedges’ method [10]

^b^Downgraded − 1 for risk of bias due to some concerns about bias due to confounding (e.g. no appropriate controlling for important confounding domains) and bias due to missing data (e.g. no sufficient documented handling of missing data)

^c^Downgraded − 1 for inconsistency due to a significant chi^2^ test and a substantial high I^2^ test (> 50%), further analysis via subgroup analysis, sensitivity analysis and meta-regression analysis were conducted

^d^Upgraded + 1 because the dose–response relation shows significant higher effect estimates when the Stringency Index was > 60 or the School Closure Index ≥ 2

^e^Downgraded − 1 for indirectness due to moderate confidence intervals and overlap of the line of no effect of the 95% confidence interval in total effect estimate, although a large sample size

### Meta-analysis of clinically relevant depression rates

**Fig. 2:**
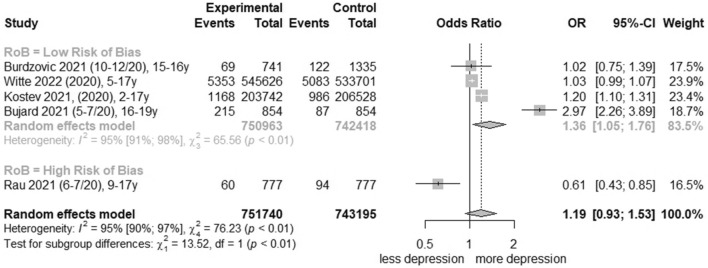
Forest plot of changes in youth clinically relevant depression symptoms comparing before and during COVID-19 pandemic

For the comparison of change effects before and during the COVID-19 pandemic regarding clinically relevant depression rates, five studies [29, 30, 38, 41, 46] were pooled and certainty of evidence was graded as ‘low’ (Table 2; further information in Additional file 1: Table S9). Total change yielded in four low-risk-of-bias studies [29, 30, 38, 46] an OR of 1.36 (95% CI, 1.05 to 1.76, I^2^ = 95%; Fig. 2). Clinically relevant depression rates increased in females in low-risk-of-bias studies significantly (OR, 1.46 [95% CI, 1.08 to 1.97], I^2^ = 95%; Additional file 1: Fig. S14), but not for males (Additional file 1: Fig. S15). Data from Witte et al. [46] also reported stationary care in 2021 among females of 10–14 years with an OR of 1.20 (95% CI, 1.03 to 1.39; Additional file 1: Table S7) and of 15–17 years with an OR of 1.43 (95% CI, 1.30 to 1.57; Additional file 1: Table S7). Further subgroup analyses were not possible.

### Heterogeneity, publication bias and sensitivity analysis

**Fig. 3:**
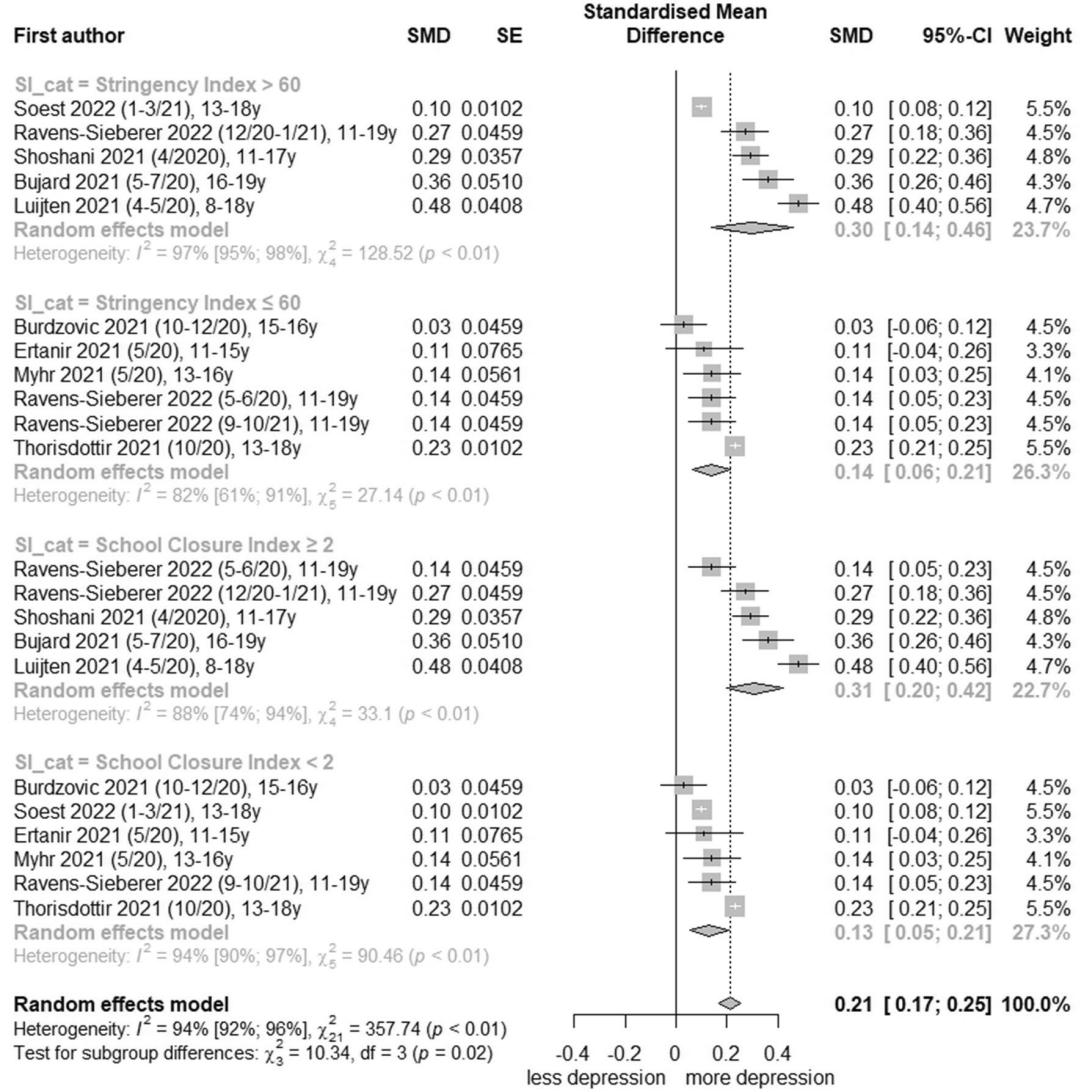
Forest plot of changes in youth general depression symptoms comparing policy indices

For each association, meta-analysis was stratified by low vs. high risk-of-bias studies (Figs. 1, 2, 3 and Additional file 1: Figs. S4–S11, S13–S15). Heterogeneity was in part substantial (I^2^ > 50%). In meta-regression analyses, the covariate ‘symptom reporter’ (b = 0.59; 95% CI, 0.08 to 1.10; p = 0.03) acts as a moderator in total population. For low-risk-of-bias studies, the meta-regression analysis revealed the covariates ‘month start data collection’ (b = − 0.01; 95% CI, − 0.02 to − 0.00; p = 0.04), ‘School Closure Index’ (b = 0.22; 95% CI, 0.10 to 0.34; p = 0.0002) and ‘country’ (Netherlands: b = 0.25; 95% CI, 0.08 to 0.42; p = 0.004; Norway: b = − 0.14; 95% CI, − 0.25 to − 0.02; p = 0.02). All moderator analyses are presented in the Additional file 1: Tables S10-S17. Sensitivity analyses were performed by comparison of (1) cohort vs. cross-sectional studies, (2) converted vs. unconverted effect estimates and (3) adjusted vs. unadjusted effect estimates for all studies and low-risk-of-bias studies. Except for a notable change in the comparison of adjusted and unadjusted values, no divergent results were observed (Additional file 1: Table S18). In total, the (contour-enhanced) funnel plots were not asymmetrical (Additional file 1: Figs. S16–S21). Also, Eggers’ test was not significant, neither for total population nor for female and male population in any age category (Additional file 1: Table S19).

## Discussion

To our knowledge, this is the first review that systematically reviewed the evidence regarding changes to depression among children and adolescents in Europe after the onset of the COVID-19 pandemic. The pooled effect estimates of low-risk-of-bias studies-comparing effects before and during the COVID-19 pandemic-revealed a significant increase of general depression symptoms for total, female and male children and adolescents; thereby, increase for male adolescents was higher, in particular for the age category 16–19 years. The comparison between countries revealed a dose–response-relationship in that estimates for general depression symptoms were significantly higher when PHSM were more stringent or school closure (partial) occurred. Certainty of evidence for general depression symptoms was graded as ‘moderate’. Furthermore, an increase in clinically relevant depression rates could be shown for total and female children and adolescents, graded as ‘low’ regarding certainty of evidence.

PHSM implemented in the European region have affected both the activities and the settings that are of major relevance to the youth mental health. Previously, children and adolescents have been found to be particularly vulnerable to the unintended effects of quarantine and isolation, especially with regards to depression symptoms [48, 49], which can last up to nine years after exposure [49]. Besides, almost 76 million school children in Europe [50] have been affected by full or partial school closures and other PHSM implemented in the school setting. This, lead to remarkable reduction in social contacts with peers, a disruption of daily routines, and an increase in loneliness, which appear to have major effects on the pathogenesis of depression in youths [1, 51, 52]. Our review supports previous findings by showing an increase for general depression symptoms in children and adolescents when PHSM were more rigorous and schools were closed or partially closed. Whereas the pandemic might have also been a stressor due to illness, loss of relatives and economic burdens, this paper proves the impact of social distancing policies on depression.

Our analyses indicates an increase of depression for total children and adolescents, as well as in gender and age-related subgroups (except for female adolescents of 16–19 years), which is in line with other high-quality systematic reviews [51, 53]. However, in our pooled analyses, the increase was higher among males and highest for male adolescents aged 16–19 years, which seem to contrast with previous studies [2, 51, 53]. Possible explanations for the different gender tendencies could be that the COVID-19 pandemic affects depression pathogenesis in varying gender-dependent ways related to contextual conditions: Male children and adolescents had, in comparison to female children and adolescents, lower pre-pandemic depression scores [2] and thus had the potential to experience higher increases, whilst females already had located higher depression levels [2, 53]. Whereas the increase in general depression symptoms for female children and adolescents is less, clinically relevant depression rates rose for them considerably. Also, a further later increase in female youth can be supposed as the report of Witte et al. [46] described a high rise in inpatient depression care for female adolescents aged 15–17 years in 2021. This is supported by other studies reporting a sharp decline in access to health care facilities at the beginning of the pandemic [54, 55].

While previous global analyses have shown an increase of depressive symptoms over the course of the pandemic [53], depression seemed to slightly flatten in the general European context for the youth population. However, for this association a country moderation can be assumed; the meta-regression analysis highlighted the countries of the Netherlands (positive estimate) and Norway (negative estimate) as potential covariates. Norway utilized lower PHSM and less frequent school closures [6] in the COVID-19 pandemic than other countries. Also, two Norwegian studies [30, 47] collected data at a later time point (winter 2020/spring 2021), and the pooled effect estimate for this time frame might be minimized by this.

As a consequence, to these different trends within countries-and our results on depression increases in specific age group-children and adolescents of the cohorts 2001–2010 (aged 11–19 in the years 2020–2021) should be monitored in particular for the next years. The monitoring should include in- and outpatient treatment by child and adolescent psychiatrists, child and adolescent psychotherapists, mental health services, and child protection services. Since these professionals were understaffed at the onset of the pandemic [1] and the demand for depression treatment is increasing during the COVID-19 pandemic, policy makers should considerably strengthen the resources [56]. Early screening and adequate diagnostic procedures are of utmost importance allowing to implement stepped care approaches. On an early intervention or indicated prevention level teachers and school social workers should be sensitized on increased depression risks and their characteristics. Thus, patients with urgently needed inpatient treatment e.g. suicidal patients could be discovered at an early stage and referred to protective treatment. Patients and families that need counseling can be reached with online therapies etc. Until now we lack studies on the effect of online outpatient treatment during phases of school closures. In many places in Europe these treatment alternatives have been quickly implemented [57]. But it was impossible to study the effects of these changes in the treatment setting in real head to head comparison studies. Although many care providers tried their best to maintain treatment relationships with online approaches we have to assume that these setting changes could be less effective than usual psychotherapy. With growing waiting lists in the services an increasing number of patients in need could not be served in a timely manner. Not addressing depressive symptoms at young age is strongly associated with recurrent depression in later life [58, 59] and other mental disorders, such as anxiety symptoms [60] and sleep disturbances [61], and an increased risk for suicide attempts and completion [58]. To mitigate depression effects during the COVID-19 pandemic in children and adolescents, studies have also highlighted some protective determinants, including a positive parent–child communication [62], robust family structures [63], social contacts to peers [64], physical activity [65] and green time [66]. These determinants need to be supported e.g. by family support and counseling services.

A research gap is evident for countries in Eastern Europe, children ≤ 11 years and socioeconomic subgroups (e.g. social status, education, financial resources). Future studies should use broad longitudinal population samples with pre-pandemic baselines and representativeness for their respective country if possible, validated instruments for depression measuring with a verified cut-off for clinically relevant symptoms, detailed statistical analysis with subgroup stratification for at least gender and age and an appropriate handling of confounders. Also, quasi-experimental designs should be used to highlight PHSM-related differences for depression development in children and adolescents.

### Strengths and limitations

This review largely adheres to the methodological recommendation of the Cochrane Handbook [10]. This include searches conducted in multiple databases, independent screening and risk of bias assessments, literature search (including pre-prints, grey literature and conference abstracts) with a peer-reviewed search strategy, retrieval of unpublished data, risk of bias assessment using a validated tool and using the GRADE approach.

This review has limitations. First, the majority of included studies fails to control for potential confounders, which is why we had to downgrade for risk of bias in GRADE. Second, instruments used for assessing depression varied greatly; we tried to limit the impact by calculating SMD and OR as standard effect estimates. Third, more subgroup analyses were not possible. Fourth, high heterogeneity within the meta-analyses (I^2^ > 50%) existed, a part that could be explained by meta-regression analyses. Fifth, the studies included in this review only covered a limited time frame. Sixth, the Oxford COVID-19 Stringency Index and School Closure Index represent proxies regarding PHSM and school closures that might be imprecise.

## Conclusions

This meta-analysis shows an increase in general depression symptoms and clinically relevant depression rates for European children and adolescents compared to pre-pandemic baselines, whereas the increase in general depression symptoms was higher for male adolescents and clinically relevant depression rates were higher for females. Also, rigorous PHSM and school closures resulted in a higher effect increase for general depression symptoms. Therefore, there is an urgent need for a long-term monitoring of depression and other internalizing symptoms among children and adolescents, particularly those cohorts affected by the COVID-19 pandemic, for the coming decades. As depression leads to participation deficits and increases the risk for suicidality, the long-term effects of the observed changes have to be (clinically) addressed by early intervention and indicated prevention measures. PHSM affected children and adolescents should be weighed with the most careful consideration and scientific expertise, as they can contribute to a worsening of child and adolescent mental health.

## Supplementary Information

**Additional file 1: Table S1.** PRISMA item checklist for systematic reviews (need modification after review). **Table S2.** Searched congresses and websites of key organizations. **Table S3.** Search Strategy. **Table S4.** Criteria for assessing Risk of Bias (RoB) using the RoB instrument for non-randomized studies of exposure. **Table S5.** Criteria for grading evidence according to Grading of Recommendations, Assessment, Development and Evaluations (GRADE). **Table S6.** Reasons for exclusion of studies from the systematic literature search, after screening for title and abstract. **Table S7.** Summary of effect estimates. **Table S8.** Summary of details on risk of bias (RoB) assessment in included studies. **Table S9.** Evidence profile for grading evidence according to Grading of Recommendations, Assessment, Development and Evaluations (GRADE). **Table S10.** Moderator analysis for total sample with categorical moderators. **Table S11.** Moderator analysis for total sample of low risk of bias studies with categorical moderators. **Table S12.** Moderator analysis for total sample with continuous moderators. **Table S13.** Moderator analysis for total sample of low risk of bias with continuous moderators. **Table S14.** Moderator analysis for female subsample with categorical moderators. **Table S15.** Moderator analysis for female subsample with continuous moderators. **Table S16.** Moderator analysis for male subsample with categorical moderators. **Table S17.** Moderator analysis for male subsample with continuous moderators. **Table S18.** Sensitivity analysis. **Table S19.** Eggers’ test. **Figure S1.** PRISMA Flow Chart. **Figure S2.** Traffic light plots of the domain-level judgements for each individual result. **Figure S3.** Weighted bar plots of the distribution of risk of bias judgements within each bias domain. **Figure S4.** Forest Plot of Changes in Female General Depression Symptoms Comparing Before and During COVID-19 Pandemic. **Figure S5.** Forest Plot of Changes in Male General Depression Symptoms Comparing Before and During COVID-19 Pandemic. **Figure S6.** Forest Plot of Changes in Total (11-15 years) General Depression Symptoms Comparing Before and During COVID-19 Pandemic. **Figure S7.** Forest Plot of Changes in Female (11-15 years) General Depression Symptoms Comparing Before and During COVID-19 Pandemic. **Figure S8.** Forest Plot of Changes in Male (11-15 years) General Depression Symptoms Comparing Before and During COVID-19 Pandemic. **Figure S9.** Forest Plot of Changes in Total (16-19 years) General Depression Symptoms Comparing Before and During COVID-19 Pandemic. **Figure S10.** Forest Plot of Changes in Male (16-19 years) General Depression Symptoms Comparing Before and During COVID-19 Pandemic. **Figure S11.** Forest Plot of Changes in Female (16-19 years) General Depression Symptoms Comparing Before and During COVID-19 Pandemic. **Figure S12.** Plot of change effects (standardized mean differences and 95% confidence interval) from low RoB studies on a time axis. **Figure S13.** Forest Plot of Country Changes in General Depression Symptoms Comparing Before and During COVID-19 Pandemic. **Figure S14.** Forest Plot of Changes in Female Clinically Relevant Depression Rates Comparing Before and During COVID-19 Pandemic. **Figure S15.** Forest Plot of Changes in Male Clinically Relevant Depression Rates Comparing Before and During COVID-19 Pandemic. **Figure S16.** Funnel Plot of Changes in Total General Depression Symptoms Comparing Before and During COVID-19 Pandemic. **Figure S17.** Funnel Plot of Changes in Female General Depression Symptoms Comparing Before and During COVID-19 Pandemic. **Figure S18.** Funnel Plot of Changes in Male General Depression Symptoms Comparing Before and During COVID-19 Pandemic. **Figure S19.** Funnel Plot of Changes in Total Clinically Relevant Depression Symptoms Comparing Before and During COVID-19 Pandemic. **Figure S20.** Funnel Plot of Changes in Female Clinically Relevant Depression Symptoms Comparing Before and During COVID-19 Pandemic. **Figure S21.** Funnel Plot of Changes in Male Clinically Relevant Depression Symptoms Comparing Before and During COVID-19 Pandemic. **Methods.** Oxford COVID-19 Stringency Index and the School Closure Index.

## Data Availability

All data are included in the manuscript and appendix.

## Abbreviations

CI: Confidence interval
GRADE: Grading of Recommendations Assessment, Development and Evaluation
PHSM: Public health and social measures
PRISMA: Preferred Reporting Items for Systematic Reviews and Meta-analysis
PROSPERO: International Prospective Register of Systematic Reviews
SMD: Standardized mean difference
