# Supplementary material for: Increase of depression among children and adolescents after the onset of the COVID-19 pandemic in Europe: a systematic review and meta-analysis
Source: Child Adolesc Psychiatry Ment Health. 2022 Dec 31;16:109. doi: 10.1186/s13034-022-00546-y (PMC9805372; doi:10.1186/s13034-022-00546-y)
Supplement: Supplementary file 1 — Additional file 1: Table S1. PRISMA item checklist for systematic reviews (need modification after review). Table S2. Searched congresses and websites of key organizations. Table S3. Search Strategy. Table S4. Criteria for assessing Risk of Bias (RoB) using the RoB instrument for non-randomized studies of exposure. Table S5. Criteria for grading evidence according to Grading of Recommendations, Assessment, Development and Evaluations (GRADE). Table S6. Reasons for exclusion of studies from the systematic literature search, after screening for title and abstract. Table S7. Summary of effect estimates. Table S8. Summary of details on risk of bias (RoB) assessment in included studies. Table S9. Evidence profile for grading evidence according to Grading of Recommendations, Assessment, Development and Evaluations (GRADE). Table S10. Moderator analysis for total sample with categorical moderators. Table S11. Moderator analysis for total sample of low risk of bias studies with categorical moderators. Table S12. Moderator analysis for total sample with continuous moderators. Table S13. Moderator analysis for total sample of low risk of bias with continuous moderators. Table S14. Moderator analysis for female subsample with categorical moderators. Table S15. Moderator analysis for female subsample with continuous moderators. Table S16. Moderator analysis for male subsample with categorical moderators. Table S17. Moderator analysis for male subsample with continuous moderators. Table S18. Sensitivity analysis. Table S19. Eggers’ test. Figure S1. PRISMA Flow Chart. Figure S2. Traffic light plots of the domain-level judgements for each individual result. Figure S3. Weighted bar plots of the distribution of risk of bias judgements within each bias domain. Figure S4. Forest Plot of Changes in Female General Depression Symptoms Comparing Before and During COVID-19 Pandemic. Figure S5. Forest Plot of Changes in Male General Depression Symptoms Comparing Before and During COVID-19 P [file 13034_2022_546_MOESM1_ESM.docx]

**Additional Information**

**Increase of depression among children and adolescents after the onset of the COVID-19 pandemic in Europe: A systematic review and meta-analysis**

Helena Ludwig-Walz, PhD; Indra DANNHEIM, MA; Lisa M. Pfadenhauer, PhD; Jörg M. FEGERT, MD; Martin BUJARD, PhD

Legend

[Table S1: PRISMA item checklist for systematic reviews 3](#_Toc123283929)

[Table S2: Searched congresses and websites of key organizations 6](#_Toc123283930)

[Table S3: Search Strategy 7](#_Toc123283931)

[Table S4: Criteria for assessing Risk of Bias (RoB) using the RoB instrument for non-randomized studies of exposures 13](#_Toc123283932)

[Table S5: Criteria for grading evidence according to Grading of Recommendations, Assessment, Development and Evaluations (GRADE) 16](#_Toc123283933)

[Table S6: Reasons for exclusion of studies from the systematic literature search, after screening for title and abstract 18](#_Toc123283934)

[Table S7. Summary of effect estimates 19](#_Toc123283935)

[Table S8: Summary of details on risk of bias (RoB) assessment in included studies 40](#_Toc123283936)

[Table S9. Evidence profile for grading evidence according to Grading of Recommendations, Assessment, Development and Evaluations (GRADE) 62](#_Toc123283937)

[Table S10. Moderator analysis for total sample with categorical moderators 63](#_Toc123283938)

[Table S11. Moderator analysis for total sample of low risk of bias studies with categorical moderators 63](#_Toc123283939)

[Table S12. Moderator analysis for total sample with continuous moderators 63](#_Toc123283940)

[Table S13. Moderator analysis for total sample of low risk of bias with continuous moderators 63](#_Toc123283941)

[Table S14. Moderator analysis for female subsample with categorical moderators 64](#_Toc123283942)

[Table S15. Moderator analysis for female subsample with continuous moderators 64](#_Toc123283943)

[Table S16. Moderator analysis for male subsample with categorical moderators 64](#_Toc123283944)

[Table S17. Moderator analysis for male subsample with continuous moderators 64](#_Toc123283945)

[Table S18. Sensitivity analysis 65](#_Toc123283946)

[Table S19. Eggers’ test 65](#_Toc123283947)

[Figure S1: PRISMA Flow Chart 66](#_Toc123283948)

[Figure S2: Traffic light plots of the domain-level judgements for each individual result 67](#_Toc123283949)

[Figure S3: Weighted bar plots of the distribution of risk of bias judgements within each bias domain 68](#_Toc123283950)

[Figure S4: Forest Plot of Changes in Female General Depression Symptoms Comparing Before and During COVID-19 Pandemic 68](#_Toc123283951)

[Figure S5: Forest Plot of Changes in Male General Depression Symptoms Comparing Before and During COVID-19 Pandemic 69](#_Toc123283952)

[Figure S6. Forest Plot of Changes in Total (11-15 years) General Depression Symptoms Comparing Before and During COVID-19 Pandemic 70](#_Toc123283953)

[Figure S7. Forest Plot of Changes in Female (11-15 years) General Depression Symptoms Comparing Before and During COVID-19 Pandemic 71](#_Toc123283954)

[Figure S8. Forest Plot of Changes in Male (11-15 years) General Depression Symptoms Comparing Before and During COVID-19 Pandemic 72](#_Toc123283955)

[Figure S9. Forest Plot of Changes in Total (16-19 years) General Depression Symptoms Comparing Before and During COVID-19 Pandemic 73](#_Toc123283956)

[Figure S10. Forest Plot of Changes in Male (16-19 years) General Depression Symptoms Comparing Before and During COVID-19 Pandemic 74](#_Toc123283957)

[Figure S11. Forest Plot of Changes in Female (16-19 years) General Depression Symptoms Comparing Before and During COVID-19 Pandemic 75](#_Toc123283958)

[Figure S12. Plot of change effects (standardized mean differences and 95% confidence interval) from low RoB studies on a time axis 76](#_Toc123283959)

[Figure S13. Forest Plot of Country Changes in General Depression Symptoms Comparing Before and During COVID-19 Pandemic 77](#_Toc123283960)

[Figure S14. Forest Plot of Changes in Female Clinically Relevant Depression Rates Comparing Before and During COVID-19 Pandemic 78](#_Toc123283961)

[Figure S15. Forest Plot of Changes in Male Clinically Relevant Depression Rates Comparing Before and During COVID-19 Pandemic 78](#_Toc123283962)

[Figure S16. Funnel Plot of Changes in Total General Depression Symptoms Comparing Before and During COVID-19 Pandemic 79](#_Toc123283963)

[Figure S17. Funnel Plot of Changes in Female General Depression Symptoms Comparing Before and During COVID-19 Pandemic 80](#_Toc123283964)

[Figure S18. Funnel Plot of Changes in Male General Depression Symptoms Comparing Before and During COVID-19 Pandemic 81](#_Toc123283965)

[Figure S19. Funnel Plot of Changes in Total Clinically Relevant Depression Symptoms Comparing Before and During COVID-19 Pandemic 82](#_Toc123283966)

[Figure S20. Funnel Plot of Changes in Female Clinically Relevant Depression Symptoms Comparing Before and During COVID-19 Pandemic 83](#_Toc123283967)

[Figure S21. Funnel Plot of Changes in Male Clinically Relevant Depression Symptoms Comparing Before and During COVID-19 Pandemic 84](#_Toc123283968)

[Methods: Oxford COVID-19 Stringency Index and the School Closure Index 85](#_Toc123283969)

[References 86](#_Toc123283970)

# Table S1: PRISMA item checklist for systematic reviews

| **Topic** | **No.** | **Item** | **Location where item is reported** |
| --- | --- | --- | --- |
| **TITLE** |  |  |  |
| **Title** | 1 | Identify the report as a systematic review. | Title |
| **ABSTRACT** |  |  |  |
| **Abstract** | 2 | See the PRISMA 2020 for Abstracts checklist | not applicable |
| **INTRODUCTION** |  |  |  |
| **Rationale** | 3 | Describe the rationale for the review in the context of existing knowledge. | Background |
| **Objectives** | 4 | Provide an explicit statement of the objective(s) or question(s) the review addresses. | Background |
| **METHODS** |  |  |  |
| **Eligibility criteria** | 5 | Specify the inclusion and exclusion criteria for the review and how studies were grouped for the syntheses. | Methods/Search strategy and selection criteria |
| **Information sources** | 6 | Specify all databases, registers, websites, organisations, reference lists and other sources searched or consulted to identify studies. Specify the date when each source was last searched or consulted. | Methods/Search strategy and selection criteria |
| **Search strategy** | 7 | Present the full search strategies for all databases, registers and websites, including any filters and limits used. | Table S3 |
| **Selection process** | 8 | Specify the methods used to decide whether a study met the inclusion criteria of the review, including how many reviewers screened each record and each report retrieved, whether they worked independently, and if applicable, details of automation tools used in the process. | Methods/Search strategy and selection criteria |
| **Data collection process** | 9 | Specify the methods used to collect data from reports, including how many reviewers collected data from each report, whether they worked independently, any processes for obtaining or confirming data from study investigators, and if applicable, details of automation tools used in the process. | Methods/Data analysis |
| **Data items** | 10a | List and define all outcomes for which data were sought. Specify whether all results that were compatible with each outcome domain in each study were sought (e.g. for all measures, time points, analyses), and if not, the methods used to decide which results to collect. | Methods/Data analysis |
|  | 10b | List and define all other variables for which data were sought (e.g. participant and intervention characteristics, funding sources). Describe any assumptions made about any missing or unclear information. | Methods/Data analysis |
| **Study risk of bias assessment** | 11 | Specify the methods used to assess risk of bias in the included studies, including details of the tool(s) used, how many reviewers assessed each study and whether they worked independently, and if applicable, details of automation tools used in the process. | Methods/Data analysis |
| **Effect measures** | 12 | Specify for each outcome the effect measure(s) (e.g. risk ratio, mean difference) used in the synthesis or presentation of results. | Methods/Data analysis |
| **Synthesis methods** | 13a | Describe the processes used to decide which studies were eligible for each synthesis (e.g. tabulating the study intervention characteristics and comparing against the planned groups for each synthesis (item 5)). | Methods/Data analysis |
|  | 13b | Describe any methods required to prepare the data for presentation or synthesis, such as handling of missing summary statistics, or data conversions. | Methods/Data analysis |
|  | 13c | Describe any methods used to tabulate or visually display results of individual studies and syntheses. | Methods/Data analysis |
|  | 13d | Describe any methods used to synthesize results and provide a rationale for the choice(s). If meta-analysis was performed, describe the model(s), method(s) to identify the presence and extent of statistical heterogeneity, and software package(s) used. | Methods/Data analysis |
|  | 13e | Describe any methods used to explore possible causes of heterogeneity among study results (e.g. subgroup analysis, meta-regression). | Methods/Data analysis |
|  | 13f | Describe any sensitivity analyses conducted to assess robustness of the synthesized results. | Methods/Data analysis |
| **Reporting bias assessment** | 14 | Describe any methods used to assess risk of bias due to missing results in a synthesis (arising from reporting biases). | Methods/Data analysis |
| **Certainty assessment** | 15 | Describe any methods used to assess certainty (or confidence) in the body of evidence for an outcome. | Methods/Data analysis |
| **RESULTS** |  |  |  |
| **Study selection** | 16a | Describe the results of the search and selection process, from the number of records identified in the search to the number of studies included in the review, ideally using a flow diagram. | Results, Figure S1 |
|  | 16b | Cite studies that might appear to meet the inclusion criteria, but which were excluded, and explain why they were excluded. | Table S7 |
| **Study characteristics** | 17 | Cite each included study and present its characteristics. | Table 1 |
| **Risk of bias in studies** | 18 | Present assessments of risk of bias for each included study. | Table S4, Table S8, Figure S2, FigureS3 |
| **Results of individual studies** | 19 | For all outcomes, present, for each study: (a) summary statistics for each group (where appropriate) and (b) an effect estimate and its precision (e.g. confidence/credible interval), ideally using structured tables or plots. | Table S7 |
| **Results of syntheses** | 20a | For each synthesis, briefly summarise the characteristics and risk of bias among contributing studies. | Results, Figure 1-3 |
|  | 20b | Present results of all statistical syntheses conducted. If meta-analysis was done, present for each the summary estimate and its precision (e.g. confidence/credible interval) and measures of statistical heterogeneity. If comparing groups, describe the direction of the effect. | Results,  Figure 1-3, Figure S4-15 |
|  | 20c | Present results of all investigations of possible causes of heterogeneity among study results. | Results, Figure S10-S17 |
|  | 20d | Present results of all sensitivity analyses conducted to assess the robustness of the synthesized results. | Results,  Table S18, Figure S16-21 |
| **Reporting biases** | 21 | Present assessments of risk of bias due to missing results (arising from reporting biases) for each synthesis assessed. | Results,  Table S19, Figure S16-21 |
| **Certainty of evidence** | 22 | Present assessments of certainty (or confidence) in the body of evidence for each outcome assessed. | Results, Table S5, Table S9 |
| **DISCUSSION** |  |  |  |
| **Discussion** | 23a | Provide a general interpretation of the results in the context of other evidence. | Discussion |
|  | 23b | Discuss any limitations of the evidence included in the review. | Discussion |
|  | 23c | Discuss any limitations of the review processes used. | Discussion |
|  | 23d | Discuss implications of the results for practice, policy, and future research. | Discussion |
| **OTHER INFORMATION** |  |  |  |
| **Registration and protocol** | 24a | Provide registration information for the review, including register name and registration number, or state that the review was not registered. | Methods/Search strategy and selection criteria |
|  | 24b | Indicate where the review protocol can be accessed, or state that a protocol was not prepared. | Methods/Search strategy and selection criteria |
|  | 24c | Describe and explain any amendments to information provided at registration or in the protocol. | Methods/Search strategy and selection criteria |
| **Support** | 25 | Describe sources of financial or non-financial support for the review, and the role of the funders or sponsors in the review. | not applicable |
| **Competing interests** | 26 | Declare any competing interests of review authors. | Competing interests |
| **Availability of data, code and other materials** | 27 | Report which of the following are publicly available and where they can be found: template data collection forms; data extracted from included studies; data used for all analyses; analytic code; any other materials used in the review. | Additional information (online) |

# Table S2: Searched congresses and websites of key organizations

| **Congresses:** | - European Public Health Conference - European Conference on Mental Health |
| --- | --- |
| **Websites of key organizations:** | - European Centre for Disease Prevention and Control - European Society for Child and Adolescent Psychiatry - European Union - Council of Europe - Save the Children - United Nations International Children's Emergency Fund (UNICEF) - World Health Organization (WHO) - Organization for Economic Co-operation and Development (OECD) |

# Table S3: Search Strategy

**MEDLINE (via Pubmed)**

| **Population** | Infan* OR toddler* OR minors OR minors* OR boy OR boys OR boyfriend OR boyhood OR girl* OR kid OR kids OR child OR child* OR children* OR schoolchild* OR schoolchild OR school child[tiab] OR school child*[tiab] OR adolescen* OR juvenil* OR youth* OR teen* OR under*age* OR pubescen* OR pediatrics[mh] OR pediatric* OR paediatric* OR peadiatric* OR school*[tiab] OR kindergarten*[tiab] OR kindergarden*[tiab] |
| --- | --- |
| **Intervention** | ("Covid-19"[Mesh] OR covid*[tiab] OR "SARS-CoV-2"[Mesh] OR “sars-2”[tiab] OR “sars2”[tiab] OR “sars 2”[tiab] OR “sars-cov-19”[tiab] OR “sars-cov19”[tiab] OR “sarscov-19”[tiab] OR “sarscov19”[tiab] OR “sars cov 19”[tiab] OR “sarscov 19”[tiab] OR “sars cov19”[tiab] OR "sars-cov-2"[tiab] OR “sarscov-2”[tiab] OR “sars-cov2”[tiab] OR “sarscov2”[tiab] OR "sars cov 2"[tiab] OR "sarscov 2"[tiab] OR "sars cov2"[tiab] OR "Severe Acute Respiratory Syndrome" [tiab] OR "Severe Acute Respiratory disease"[tiab] OR "coronavirus"[MeSH] OR Coronavirus*[tiab] OR corona-virus*[tiab] OR “corona virus*”[tiab] OR ncov*[tiab] OR n-cov*[tiab] OR “n cov”[tiab] OR novelcov*[tiab] OR novel-cov*[tiab] OR “novel cov*”[tiab]) AND (2019/11/01[PDAT] : 3000/12/31[PDAT]) |
| **Comparison** |  |
| **Outcome** | "Depression"[Mesh] OR "Depressive Disorder"[Mesh] OR depress*[tiab] OR “Anxiety”[Mesh] OR “Anxiety Disorders”[Mesh] OR anxi*[tiab] |
| **Species** | FinalResult NOT (animals[MeSH] NOT humans[MeSH]) |
| **Language** | No limit |

**MEDLINE/Embase (via Embase)**

| **Population** | Infan* OR toddler* OR minors* OR boy OR boys OR boyfriend OR boyhood OR girl* OR kid OR kids OR child* OR schoolchild* OR (‘school child’ OR ‘school child*’):ab,ti OR adolescen* OR juvenil* OR youth* OR teen* OR under*age* OR pubescen* OR 'pediatrics'/exp OR pediatric* OR paediatric* OR (‘peadiatric*’ OR ‘school’ OR ‘school*’):ab,ti OR (kindergarten* OR kindergarden*):ab,ti |
| --- | --- |
| **Intervention** | (covid*:ab,ti OR 'sars 2':ab,ti OR sars2:ab,ti OR 'sars-2':ab,ti OR 'sars-cov-19':ab,ti OR 'sars-cov19':ab,ti OR 'sarscov-19':ab,ti OR 'sarscov19':ab,ti OR 'sars cov 19':ab,ti OR 'sarscov 19':ab,ti OR 'sars cov19':ab,ti OR 'sars-cov-2':ab,ti OR 'sarscov-2':ab,ti OR 'sars-cov2':ab,ti OR 'sarscov2':ab,ti OR 'sars cov 2':ab,ti OR 'sarscov 2':ab,ti OR 'sars cov2':ab,ti OR 'severe acute respiratory syndrome':ab,ti OR 'severe acute respiratory disease':ab,ti OR coronavirus*:ab,ti OR 'corona virus*':ab,ti OR 'corona-virus*':ab,ti OR ncov*:ab,ti OR 'n-cov*':ab,ti OR 'n cov':ab,ti OR novelcov*:ab,ti OR 'novel cov*':ab,ti OR 'novel-cov*':ab,ti OR 'severe acute respiratory syndrome coronavirus 2'/exp OR 'coronavirinae'/exp OR 'coronavirus'/exp) AND [01-11-2019]/sd NOT [31-12-3000]/sd |
| **Comparison** |  |
| **Outcome** | 'depression'/exp OR depress*:ab,ti OR 'anxiety'/exp OR 'anxiety disorder'/exp OR 'anxi*':ab,ti |
| **Species** | FinalResult NOT ([animals]/lim NOT [humans]/lim) |
| **Language** | No limit |

**APA PsycInfo (via EBSCOhost)**

| **Population** | (TI (Infan* OR toddler* OR minors OR minors* OR boy OR boys OR boyfriend OR boyhood OR girl* OR kid OR kids OR child OR child* OR children* OR schoolchild* OR schoolchild OR ‘school child’ OR ‘school child*’ OR adolescen* OR juvenil* OR youth* OR teen* OR underage* OR pubescen* OR pediatrics OR pediatric* OR paediatric* OR ‘school*’ OR kindergarten* OR kindergarden*)) OR (AB (Infan* OR toddler* OR minors OR minors* OR boy OR boys OR boyfriend OR boyhood OR girl* OR kid OR kids OR child OR child* OR children* OR schoolchild* OR schoolchild OR ‘school child’ OR ‘school child*’ OR adolescen* OR juvenil* OR youth* OR teen* OR underage* OR pubescen* OR pediatrics OR pediatric* OR paediatric* OR ‘school*’ OR kindergarten* OR kindergarden*)) |
| --- | --- |
| **Intervention** | (DE "COVID-19" OR DE "Coronavirus") OR (TI covid* OR AB covid*) OR (TI covid* OR AB covid*) OR (TI "SARS-CoV-2" OR AB "SARS-CoV-2") OR (TI "sars-2*" OR AB "sars-2*") OR (TI "sars2*" OR AB "sars2*") OR (TI "sars 2" OR AB "sars 2") OR (TI “sars-cov-19” OR AB “sars-cov-19”) OR (TI “sars-cov19” OR AB “sars-cov19”) OR (TI “sarscov-19” OR AB “sarscov-19”) OR (TI “sarscov19” OR AB “sarscov19”) OR (TI “sars cov 19” OR AB “sars cov 19”) OR (TI “sarscov 19” OR AB “sarscov 19”) OR (TI “sars cov19” OR AB “sars cov19”) OR (TI “sarscov-2” OR AB “sarscov-2”) OR (TI “sars-cov-2” OR AB “sars-cov-2”) OR (TI “sarscov2” OR AB “sarscov2”) OR (TI “sars-cov2” OR AB “sars-cov2”) OR (TI “sarscov2” OR AB “sarscov2”) OR (TI “sars cov 2” OR AB “sars cov 2”) OR (TI “sarscov 2” OR AB “sarscov 2”) OR (TI “sars cov2” OR AB “sars cov2”) (TI “Severe Acute Respiratory Syndrome” OR AB “Severe Acute Respiratory Syndrome”) OR (TI “Severe Acute Respiratory disease” OR AB “Severe Acute Respiratory disease”) OR (TI “Coronavirus*” OR AB “Coronavirus*”) OR (TI “Corona-virus*” OR AB “Corona-virus*”) OR (TI “Corona virus*” OR AB “Corona virus*”) OR (TI “ncov*” OR AB “ncov*”) OR (TI “n-cov*” OR AB “n-cov*”) OR (TI “n cov*” OR AB “n cov*”) OR (TI “novelcov*” OR AB “novelcov*”) OR (TI “novel-cov*” OR AB “novel-cov*”) OR (TI “novel cov*” OR AB “novel cov*”) |
| **Comparison** |  |
| **Outcome** | (DE "Depression (Emotion)") OR (DE "Anxiety") OR (DE "Anxiety Disorders") OR (TI(depress* OR anxi*)) OR (AB(depress* OR anxi*)) |
| **Limits** | Filters: Humans *(screened manually)* |

**Cochrane Library**

| **Population** | #1 MeSH descriptor: [Infant] explode all trees  #2 MeSH descriptor: [Child] explode all trees  #3 MeSH descriptor: [Adolescent] explode all trees  #4 MeSH descriptor: [Pediatrics] explode all trees  #5 MeSH descriptor: [Schools] explode all trees  #6 (Infan* OR toddler* OR minors OR minors* OR boy OR boys OR boyfriend OR boyhood OR girl* OR kid OR kids OR child* OR schoolchild* OR schoolchild OR "school NEXT child*" OR adolescen* OR juvenil* OR youth* OR teen* OR under-age* OR pubescen* OR paediatric* OR peadiatric* OR school* OR kindergarten* OR kindergarden*):ti,kw,ab |
| --- | --- |
| **Intervention** | #7 MeSH descriptor: [COVID-19] explode all trees  #8 MeSH descriptor: [Coronavirus] explode all trees  #9 MeSH descriptor: [SARS-CoV-2] explode all trees  #10 (covid* OR sars-2 OR sars2 OR "sars NEXT 2" OR sars-cov-19 OR sars-cov19 OR sarscov-19 OR “sars NEXT cov NEXT 19” OR “sarscov NEXT 19” OR “sars NEXT cov19” OR sarscov19 OR SARS-CoV-2 OR sarscov-2 OR sars-cov2 OR sarscov2 OR "sars NEXT cov NEXT 2" OR "sarscov NEXT 2" OR "sars NEXT cov2" OR "Severe NEXT Acute NEXT Respiratory NEXT Syndrome" OR "Severe NEXT Acute NEXT Respiratory NEXT disease" OR Coronavirus* OR corona-virus* OR "corona NEXT virus*" OR ncov* OR n-cov* OR "n NEXT cov" OR novelcov* OR novel-cov* OR "novel NEXT cov*"):ti,kw,ab |
| **Comparison** |  |
| **Outcome** | #11 MeSH descriptor: [Depression] explode all trees 13588  #12 MeSH descriptor: [Anxiety] explode all trees 8823  #13 MeSH descriptor: [Anxiety Disorders] explode all trees 7539  #14 (depress* OR anxi*):ti,kw,ab |
| **Limits** | Filters: Humans *(screened manually)* |

**Web of Science Core Collection**

| **Population** | ts=(Infan* OR toddler* OR minors* OR boy OR boys OR boyfriend OR boyhood OR girl* OR kid OR kids OR child* OR children* OR schoolchild* OR schoolchild OR "school child" OR "school child" OR adolescen* OR juvenil* OR youth* OR teen* OR under*age* OR pubescen* OR kindergarten* OR kindergarden*) |
| --- | --- |
| **Intervention** | ts=(covid* OR "sars 2" OR sars2 OR sars-2 OR "sars cov 19" OR "sars cov19" OR "sarscov 19" OR sarscov19 OR "sars-cov-19" OR "sars-cov19" OR "sarscov-19" OR "sars-cov-2" OR "sarscov 2" OR "sars cov2" OR sarscov2 OR "sars cov 2" OR "sarscov-2" OR "sars-cov2" OR "severe acute respiratory syndrome" OR "severe acute respiratory disease" OR coronavirus* OR "corona virus*" OR "corona-virus*" OR ncov* OR "n-cov*" OR "n cov" OR novelcov* OR "novel cov*" OR "novel-cov*") |
| **Comparison** | NA |
| **Outcome** | ts=(depress* OR anxi*) |
| **Species** | Humans *(screened manually)* |
| **Language** | No limit |

**WHO COVID-19 database**

| **Population** | (Infan* toddler* OR minors* OR boy OR boys OR boyfriend OR boyhood OR girl* OR kid OR kids OR child* OR children* OR schoolchild* OR schoolchild OR "school child" OR "school child" OR adolescen* OR juvenil* OR youth* OR teen* OR under*age* OR pubescen* OR kindergarten* OR kindergarden*) |
| --- | --- |
| **Intervention** | (covid* OR "sars 2" OR sars2 OR sars-2 OR "sars cov 19" OR "sars cov19" OR "sarscov 19" OR sarscov19 OR "sars-cov-19" OR "sars-cov19" OR "sarscov-19" OR "sars-cov-2" OR "sarscov 2" OR "sars cov2" OR sarscov2 OR "sars cov 2" OR "sarscov-2" OR "sars-cov2" OR "severe acute respiratory syndrome" OR "severe acute respiratory disease" OR coronavirus* OR "corona virus*" OR "corona-virus*" OR ncov* OR "n-cov*" OR "n cov" OR novelcov* OR "novel cov*" OR "novel-cov*") |
| **Comparison** | NA |
| **Outcome** | (depress* OR anxi*) |
| **Species** | Humans *(screened manually)* |
| **Language** | No limit |

# Table S4: Criteria for assessing Risk of Bias (RoB) using the RoB instrument for non-randomized studies of exposures

| **Criteria** | **Definition of criteria for RoB assessment** | | | |
| --- | --- | --- | --- | --- |
| *Bias item* | *Critical RoB* | *Serious RoB* | *Moderate RoB* | *Low RoB* |
| 1. Bias due to confounding^[[1]](#footnote-1),^^[[2]](#footnote-2)^ | No use of an appropriate analysis method that controlled for all important confounding domains, e.g. stratification, regression, matching, standardization, and inverse probability.  No measurement of confounding domains validly and reliably. | Limited use of an appropriate analysis method that controlled for all important confounding domains, e.g. stratification, regression, matching, standardization, and inverse probability.  Limited measurement of confounding domains validly and reliably. | Reduced use of an appropriate analysis method that controlled for important confounding domains, e.g. stratification, regression, matching, standardization, and inverse probability.  Predominantly measurement of confounding domains validly and reliably. | Use of an appropriate analysis method that controlled for important confounding domains, e.g. stratification, regression, matching, standardization, and inverse probability.  Measurement of confounding domains validly and reliably. |
| 1. Bias in selection of participants into the study^[[3]](#footnote-3)^,^[[4]](#footnote-4)^ | Restricting the study sample to individuals, e.g. students, kindergarten children 🡪 may limit generalizability.  Unclear inclusion and exclusion criteria.  Participation rate of eligible persons < 50%.  Comparison of pre-pandemic baseline and during pandemic measure occurred in a similar population.  Follow-up rate to pre-pandemic baseline is < 50% or more (not applicable, if not the same population). | Restricting the study sample to individuals, e.g. students, kindergarten children 🡪 may limit generalizability.  Unclear inclusion and exclusion criteria.  Participation rate of eligible persons < 50%.  Comparison of pre-pandemic baseline and during pandemic measure occurred in a similar population.  Follow-up rate to pre-pandemic baseline is < 50% or more (not applicable, if not the same population). | Restricting the study sample to individuals, e.g. students, kindergarten children 🡪 may limit generalizability.  Description of clear inclusion and exclusion criteria prior to the recruitment (who, where, when).  Participation rate of eligible persons > 50%.  Comparison of pre-pandemic baseline and during pandemic measure occurred in a similar population.  Follow-up rate to pre-pandemic baseline is > 50% or more (not applicable, if not the same population). | Inclusion of general population.  Description of clear inclusion and exclusion criteria prior to the recruitment (who, where, when).  Clear definition for the exclusion of population samples.  Participation rate of eligible persons > 50%.  Comparison of pre-pandemic baseline and at-pandemic measure occurred in the same population.  Follow-up rate to pre-pandemic baseline is 80% or more. |
| 1. Bias in classification of exposures^[[5]](#footnote-5)^ | Poor description of the time point of data assessment at the COVID-19 pandemic (e.g., date, first lockdown).  Effect estimates compared to a pre-pandemic baseline similar to the population sample at COVID-19 pandemic.  Pre-pandemic assessment retrospective. | Solid description of the time point of data assessment at the COVID-19 pandemic (e.g., date, first lockdown).  Effect estimates compared to a pre-pandemic baseline similar to the population sample at COVID-19 pandemic.  Pre-pandemic assessment retrospective. | Solid description of the time point of data assessment at the COVID-19 pandemic (e.g., date, first lockdown).  Effect estimates compared to a pre-pandemic baseline similar to the population sample at COVID-19 pandemic.  Pre-pandemic assessment prospective. | Good description of the time point of data assessment at the COVID-19 pandemic (e.g., date, first lockdown).  Effect estimates compared to a pre-pandemic baseline of the same population sample at COVID-19 pandemic.  Pre-pandemic assessment prospective. |
| 1. Bias due to departures from intended exposures^[[6]](#footnote-6)^ | Not applicable. | Not applicable. | Not applicable. | Not applicable. |
| 1. Bias due to missing data | Availability of data from <70% of the participants.  No documentation of handling of missing data on variables needed for the analysis.  No evidence for robustness of the results in presence of missing data. | Availability of data from <75% of the participants.  Documented handling of missing data on variables needed for the analysis.  Limited evidence for robustness of the results in presence of missing data. | Availability of data from >75% of the participants.  Documented and comprehensible handling of missing data on variables needed for the analysis.  Evidence for robustness of the results in presence of missing data. | Availability of data from >80% of the participants.  Documented and comprehensible handling of missing data on variables needed for the analysis.  Evidence for robustness of the results in presence of missing data. |
| 1. Bias in measurements of outcomes | Use of different methods for the whole population sample  No use of standardized and validated measurements of outcome in accordance with the International Consortium for Health Outcomes Measurements.  No self-report of participants ≥ 11 years of age.  Possibility of any systematic errors in measuring, e.g. for age, sex, school visit. | Use of the similar methods for the whole population sample.  No use of standardized and validated measurements of outcome.  No self-report of participants ≥ 11 years of age.  Unlikely of any systematic errors in measuring, e.g. for age, sex, school visit. | Use of the same methods for the whole population sample.  Use of standardized and validated measurements of outcome in combination with non-standardized measurements, like self-assessments.  No self-report of participants ≥ 11 years of age. Physician-reported.  Unlikely of any systematic errors in measuring, e.g. for age, sex, school visit. | Use of the same methods for the whole population sample.  Use of standardized and validated measurements of outcome.  Self-report of participants ≥ 11 years of age and parent-proxy reports of participants < 11 years of age. Physician-reported.  Unlikely of any systematic errors in measuring, e.g. for age, sex, school visit. |

| 1. Bias in selection of reported results | Inconsistence of reporting of the results with a priori plan  Uncertainty if statistical methods reported in the methods section were used in the results or discussion section.  Probability that 3 of the 3 selective reporting bias emerged | Inconsistence of reporting of the results with a priori plan  Uncertainty if statistical methods reported in the methods section were used in the results or discussion section.  Probability that 2 of the 3 selective reporting bias emerged | Consistence of reporting of the results with a priori plan.  Statistical methods reported in the methods section were used and presented in the results or discussion section.  Probability that 1 of the 3 selective reporting bias emerged | Consistence of reporting of the results with a priori plan  Statistical methods reported in the methods section were used in the results or discussion section.  No selective outcome reporting (e.g. selective reporting of measurement made at one of a number of time points or based on one of multiple depression/anxiety scales).  No selective analysis reporting (e.g. reporting of effects estimated in multiple ways, analyses with adjustment for different sets of potential confounders).  No selective reporting (e.g. reporting of subgroup of participants, selected from a larger cohort, for which results are reported on the basis of a more interesting finding). |
| --- | --- | --- | --- | --- |
| **Overall RoB** | **Study is judged to be at critical risk of bias in at least one domain.** | **Study is judged to be at serious risk of bias in at least one domain, but not at critical risk of bias in any domain.** | **Study is judged to be at low or moderate risk of bias for all domains.** | **Study is judged to be at low risk of bias for all domains.** |

# Table S5: Criteria for grading evidence according to Grading of Recommendations, Assessment, Development and Evaluations (GRADE)

Developed under consideration of the criteria reported by Schünemann et al. 2013 [2], Schünemann et al. 2019 [3] and Morgan et al. 2019 [1].

| **Domain** | **Application in this review** | **Interpretation** | **GRADE assessment** |
| --- | --- | --- | --- |
| *Reasons for considering lowering certainty* | |  |  |
| Risk of Bias (RoB) | Based on the RoB assessment using the RoB instrument for non-randomized studies of exposures [1]. | 1. Most information is from results at low risk of bias. 2. Most information is from results at low risk of bias or with some concerns; potential limitations are unlikely to lower confidence in the estimate of effect. 3. Most information is from results at low risk of bias or with some concerns; potential limitations are likely to lower confidence in the estimate of effect. 4. The proportion of information from results at high risk of bias is sufficient to affect the interpretation of results; crucial limitation for one criterion, or some limitations for multiple criteria, sufficient to lower confidence in the estimate of effect. 5. The proportion of information from results at high risk of bias is sufficient to affect the interpretation of results; crucial limitation for one or more criteria sufficient to substantially lower confidence in the estimate of effect. | 1. No serious limitations, not downgrade. 2. No serious limitations, not downgrade. 3. Serious limitations, downgrade 1 level. 4. Serious limitations, downgrade 1 level. 5. Very serious limitations, downgrade 2 levels. |
| Inconsistency | Existence of heterogeneity, if   1. Wide variance of point estimates across studies 2. Minimal or no overlap of confidence intervals (CI) 3. Statistical criteria: chi^2^ test and I² statistic    - Significant p-value in chi² test 🡪 indication of heterogeneity    - I² statistic    - < 40% 🡪 heterogeneity may be low    - 30-60% 🡪 heterogeneity may be moderate    - 50-90% 🡪 heterogeneity may be substantial    - 75-100% 🡪 heterogeneity may be considerable | - - - 1. No wide variance of point estimates, minimal or no overlap of CI, chi² test not significant,  I² <60%.       2. Chi² test significant, I² test >50%, further analysis via subgroup analysis, sensitivity analysis, meta-regression analysis.       3. Chi² test significant, I² test >50%, no further analysis. | 1. No serious limitations, not downgrade. 2. Serious limitations, downgrade 1 level. 3. Very serious limitations, downgrade 2 levels. |
| Indirectness | Applicability of the evidence to the relevant research question. Fitting of the characteristics of the included studies to the predefined PECO scheme. | - - - 1. Studies address exactly the research question.       2. Studies indirectly (indirect comparisons) or imprecisely (restricted version of the main review question) address the research question, but an adequate transfer is possible.       3. Studies indirectly (indirect comparisons) or imprecisely (restricted version of the main review question) address the research question, but an adequate transfer is not possible. | 1. No serious limitations, not downgrade. 2. Serious limitations, downgrade 1 level. 3. Very serious limitations, downgrade 2 levels. |
| Imprecision | Risks of random errors because of small sample sizes, broad CIs or inclusion of the “no effect line” in 95% CI | - - - 1. Broad sample size with small to moderate confidence intervals and no overlap of the “no effect line” (mean/SMD=0, OR/RR=1) regarding the 95% CI of the pooled effect.       2. Moderate to broad sample size, with moderate confidence intervals and overlap of the line of no effect of the 95% CI.       3. Small sample size, with moderate to broad confidence intervals and overlap of the line of no effect of the 95% CI. | 1. No serious limitations, not downgrade. 2. Serious limitations, downgrade 1 level. 3. Very serious limitations, downgrade 2 levels. |
| Publication bias | Under-estimation or over-estimation of the underlying beneficial or harmful effect due to the selective publication of studies if   - - Asymmetrical funnel plot   - Significant Egger’s Test when at least 10 studies | 1. No funnel plot asymmetry and no significance in Egger’s Test. 2. Funnel plot asymmetry and/or significance in Egger’s Test. | 1. No serious limitations, not downgrade. 2. Serious limitations, downgrade 1 level. |
|  |  |  |  |
| *Reasons for considering upgrade certainty* | |  |  |
| Large effect | Rating up when effects in observational studies are sufficiently large, particularly if they occur over short periods of time. | 1. RR >2 or RR <0.5 2. RR >5 or RR <0.2   *Note from the GRADE Handbook [2]: these rules apply when effect measure is expressed as relative risk (RR) or hazard ratio (HR). They cannot always be applied when the effect measure is expressed as odds ratio (OR). We suggest converting OR to RR and only then assessing the magnitude of an effect.* | - - - 1. May upgrade 1 level.       2. May upgrade 2 levels. |
| Dose-response | Rating up for a dose-response gradient if effect estimates are higher when pandemic related restrictions are rigorous, measured by the proxy variables “Oxford COVID-19 Stringency Index” and the “School Closure Index”. | 1. No/small differences in pandemic-related restrictions and no substantial subgroup differences. 2. Effect estimates for severe pandemic-related restrictions higher with substantial subgroup differences to moderate/lead restrictions. | 1. No upgrade. 2. May upgrade 1 level. |
| All plausible confounding and bias | All plausible residual confounding from observational studies may be working to reduce the demonstrated effect or increase the effect, if no effect was observed. | 1. No underestimation of an apparent treatment effect. 2. Underestimate of an apparent treatment effect. | 1. No upgrade. 2. May upgrade 1 level. |
|  |  |  |  |
| **Certainty of evidence** | **High – Moderate – Low – Very low** | | |

# Table S6: Reasons for exclusion of studies from the systematic literature search, after screening for title and abstract

| **Study** | **Reason for exclusion** |
| --- | --- |
| Achterberg et al. 2021 | No data on depression or anxiety were reported. |
| Albrecht et al. 2022 | No pre-pandemic baseline was reported. |
| Allgaier et al. 2022 | No data on depression or anxiety were reported. |
| Alt et al. 2021 | Same study population as in Bujard et al. 2021 [4]. |
| Daniunaite et al. 2021 | No data on depression or anxiety were reported. |
| Dollberg et al. 2021 | No data on depression or anxiety were reported. |
| Dragun et al. 2020 | Inappropriate study population (>19 years of age). |
| Duttweiler et al. 2022 | Inappropriate study population (USA). |
| Dzielska et al. 2021 | No data on depression or anxiety were reported. |
| Elise et al. 2021 | No data on depression or anxiety were reported. |
| Essau et al. 2021 | No pre-pandemic baseline was reported. |
| Gonzalez-Valero et al. 2020 | No data on depression or anxiety were reported. |
| Hoffmann et al. 2021 | Inappropriate study population (>19 years of age). |
| Koenig et al. 2021 | Inappropriate study population (age range 12-20 years of age). |
| Larsen et al. 2021 | No pre-pandemic baseline was reported. |
| Mohler-Kuo et al. 2021 | No pre-pandemic baseline was reported. |
| Muzi et al. 2021 | No data on depression or anxiety were reported. |
| Naumann et al. 2021 | Same study population as in Bujard et al. 2021 [4]. |
| Newlove-Delgado et al. 2021 | No data on depression or anxiety were reported. |
| Ravens-Sieberer et al. 2021[5] | Same study population as in Ravens-Sieberer et al. 2021 [6]. |
| Ravens-Sieberer et al. 2021[7] | Same study population as in Ravens-Sieberer et al. 2021 [6]. |
| Ravens-Sieberer et al. 2021[8] | Same study population as in Ravens-Sieberer et al. 2021[6]. |
| Truskauskaite-Kuneviciene et al. 2021 | Inappropriate study population (>19 years of age). |
| Ünver et al. 2022 | Inappropriate study population (Syrian refugee). |
| van der Laan et al. 2021 | No data on depression or anxiety were reported. |
| Vogel et al. 2021 | No data on depression or anxiety were reported. |

# Table S7. Summary of effect estimates

| **STUDY INFO** | **Reported Study data** | | | **(converted) effect estimate*** | | | | **Risk of bias** |
| --- | --- | --- | --- | --- | --- | --- | --- | --- |
| **First author, year** | **Outcome measurement,**  **subgroups,**  **psychometric properties** | **Adjustment** | **Reported outcome** | **During pandemic estimate, n/N or mean (SD) N** | **Pre-pandemic estimate, n/N or mean (SD) N** | **Std. Mean difference  IV, Random (95% CI)** | **Odds ratio  IV, Random (95% CI)** |  |
| **Germany** |  |  |  |  |  |  |  |  |
| Ravens-Sieberer, 2022 [6] | Center for Epidemiological Studies Depression Scale (CES-DC)  Subgroups:   - Gender - Age   Psychometric properties [9] | No adjustment | General depression symptoms  *Additional data were provided by authors* | **Total (11-19 y):**  DP1: 11.36 (3.74) 1,018  DP2: 11.89 (4.20) 1,073  DP3: 11.37 (3.98) 1,173  **Female (11-19 y):**  DP1: 11.71 (3.97) 505  DP2: 12.26 (4.45) 528  DP3: 11.68 (4.42) 568  **Male (11-19 y):**  DP1: 11.00 (3.44) 513  DP2: 11.53 (3.90) 545  DP3: 11.07 (3.49) 597  **Total (11-14 y):**  DP1: 11.48 (3.79) 572  DP2: 11.57 (4.07) 536  DP3: 11.19 (3.89) 528  **Female (11-14 y):**  DP1: 11.78 (4.04) 291  DP2: 11.82 (4.30) 255  DP3: 11.52 (4.74) 260  **Male (11-14 y):**  DP1: 11.13 (3.44) 280  DP2: 11.33 (3.83) 281  DP3: 10.89 (3.22) 263 | **Total (11-19 y):**  10.85 (3.60) 994  **Female (11-19 y):**  11.71 (4.04) 477  **Male (11-19 y):**  10.05 (2.91) 516  **Total (11-14 y):**  10.59 (3.39) 641  **Female (11-14 y):**  11.13 (3.81) 306  **Male (11-14 y):**  10.10 (2.87) 335 | **Total (11-19 y):**  DP1: 0.14 (0.05 to 0.23)³  DP2: 0.27 (0.18 to 0.36)³  DP3: 0.14 (0.05 to 0.23)³  **Female (11-19 y):**  DP1: 0.00 (-0.13 to 0.13)³  DP2: 0.13 (0.01 to 0.25)³  DP3: -0.01 (-0.13 to 0.11)³  **Male (11-19 y):**  DP1: 0.30 (0.18 to 0.42)³  DP2: 0.43 (0.31 to 0.55)³  DP3: 0.32 (0.20 to 0.44)³  **Total (11-14 y):**  DP1: 0.25 (0.13 to 0.37)³  DP2: 0.26 (0.15 to 0.37)³  DP3: 0.17 (0.05 to 0.29)³  **Female (11-14 y):**  DP1: 0.17 (0.00 to 0.34)³  DP2: 0.17 (0.00 to 0.34)³  DP3: 0.09 (-0.07 to 0.25)³  **Male (11-14 y):**  DP1: 0.33 (0.17 to 0.49)³  DP2: 0.37 (0.21 to 0.53)³  DP3: 0.26 (0.10 to 0.42)³ |  | **Moderate** |
|  |  |  |  | **Total (15-19 y):**  DP1: 11.21 (3.69) 446  DP2: 12.22 (4.30) 536  DP3: 11.51 (4.06) 645  **Female (15-19 y):**  DP1: 11.60 (3.88) 213  DP2: 12.68 (4.56) 273  DP3: 11.81 (4.38) 308  **Male (15-19 y):**  DP1: 10.84 (3.46) 233  DP2: 11.74 (3.97) 263  DP3: 11.21 (3.68) 334 | **Total (15-19 y):**  11.32 (3.92) 353  **Female (15-19 y):**  12.75 (4.25) 171  **Male (15-19 y):**  9.96 (3.00) 182 | **Total (15-19 y):**  DP1: -0.03 (-0.17 to 0.11)³  DP2: 0.22 (0.08 to 0.36)³  DP3: 0.05 (-0.08 to 0.18)³  **Female (15-19 y):**  DP1: -0.28 (-0.49 to -0.07)³  DP2: -0.02(-0.21 to 0.17)³  DP3: -0.22 (-0.40 to -0.04)³  **Male (15-19 y):**  DP1: 0.27 (0.07 to 0.47)³  DP2: 0.49 (0.30 to 0.68)³  DP3: 0.36 (0.18 to 0.54)³ |  |  |
|  |  |  | Cut off ≥15 CES-DC score | **Total (11-19 y):**  DP1: 183/1,018  DP2: 261/1,073  DP3: 231/1,179  **Female (11-19 y):**  DP1: 111/505  DP2: 148/528  DP3: 128/568  **Male (11-19 y):**  DP1: 71/513  DP2: 112/545  DP3: 101/597  **Total (11-14 y):**  DP1: 108/572  DP2: 118/536  DP3: 94/528  **Female (11-14 y):**  DP1: 67/291  DP2: 60/255  DP3: 54/260  **Male (11-14 y):**  DP1: 40/280  DP2: 57/281  DP3: 40/263  **Total (15-19 y):**  DP1: 75/446  DP2: 143/536  DP3: 137/645  **Female (15-19 y):**  DP1: 44/213  DP2: 88/273  DP3: 74/308  **Male (15-19 y):**  DP1: 31/233  DP2: 55/263  DP3: 61/334 | **Total (11-19 y):**  149/994  **Female (11-19 y):**  96/477  **Male (11-19 y):**  53/516  **Total (11-14 y):**  81/641  **Female (11-14 y):**  49/306  **Male (11-14 y):**  32/335  **Total (15-19 y):**  68/353  **Female (15-19 y):**  47/171  **Male (15-19 y):**  21/182 |  | **Total (11-19 y):**  DP1: 1.24 (0.98 to 1.57)³  DP2: 1.82 (1.46 to 2.28)³  DP3: 1.39 (1.11 to 1.74)³  **Female (11-19 y):**  DP1: 1.12 (0.82 to 1.52)³  DP2: 1.55 (1.15 to 2.07)³  DP3: 1.15 (0.86 to 1.56)³  **Male (11-19 y):**  DP1: 1.40 (0.96 to 2.05)³  DP2: 2.26 (1.59 to 3.21)³  DP3: 1.78 (1.25 to 2.54)³  **Total (11-14 y):**  DP1: 1.61 (1.18 to 2.20)³  DP2: 1.95 (1.43 to 2.66)³  DP3: 1.50 (1.08 to 2.07)³  **Female (11-14 y):**  DP1: 1.57 (1.04 to 2.36)³  DP2: 1.61 (1.06 to 2.46)³  DP3: 1.37 (0.90 to 2.11)³  **Male (11-14 y):**  DP1: 1.58 (0.96 to 2.59)³  DP2: 2.41 (1.51 to 3.84)³  DP3: 1.70 (1.03 to 2.79)³  **Total (15-19 y):**  DP1: 0.85 (0.59 to 1.22)³  DP2: 1.53 (1.10 to 2.11)³  DP3: 1.13 (0.82 to 1.56)³  **Female (15-19 y):**  DP1: 0.69 (0.43 to 1.10)³  DP2: 1.25 (0.82 to 1.91)³  DP3: 0.83 (0.55 to 1.28)³  **Male (15-19 y):**  DP1: 1.18 (0.65 to 2.13)³  DP2: 2.03 (1.18 to 3.49)³  DP3: 1.71 (1.01 to 2.92)³ |  |
| Witte,  2022 [10] | Medical classification (depression [ICD-10: F32/33])  Subgroups:   - Gender - Age   NI for psychometric properties (International Statistical Classification of Diseases and Related Health Problems [ICD]) | No adjustment | Clinically relevant depression symptoms  *Additional data were provided by authors* | **Total (5-17 y), prevalence:**  8,580/545,626  **Total (5-17 y), incidence:**  5,353/545,626  **Female (5-17 y), prevalence:**  6,014/265,213  **Female (5-17 y), incidence:**  3,755/265,213  **Male (5-17 y), prevalence:**  2,566/280,413  **Male (5-17 y), incidence:**  1,598/280,413  **Total (5-9 y), prevalence:**  292/200,054  **Total (5-9 y), incidence:**  228/200,054  **Female (5-9 y), prevalence:**  133/97,523  **Female (5-9 y), incidence:**  103/97,523  **Male (5-9 y), prevalence:**  159/102,531  **Male (5-9 y), incidence:**  125/102,531 | **Total (5-17 y), prevalence:**  8,300/533,701  **Total (5-17 y), incidence:**  5,083/533,701  **Female (5-17 y), prevalence:**  5,642/259,455  **Female (5-17 y), incidence:**  3,367/259,455  **Male (5-17 y), prevalence:**  2,658/274,246  **Male (5-17 y), incidence:**  1,716/274,246  **Total (5-9 y), prevalence:**  332/194,120  **Total (5-9 y), incidence:**  250/194,120  **Female (5-9 y), prevalence:**  159/94,492  **Female (5-9 y), incidence:**  117/94,492  **Male (5-9 y), prevalence:**  173/99,628  **Male (5-9 y), incidence:**  133/99,628 |  | **Total (5-17 y),  prevalence:**  1.01 (0.98 to 1.04)³  **Total (5-17 y),  incidence:**  1.03 (0.99 to 1.07)³  **Female (5-17 y), prevalence:**  1.04 (1.01 to 1.08)³  **Female (5-17 y), incidence:**  1.09 (1.04 to 1.14)³  **Male (5-17 y),  prevalence:**  0.94 (0.89 to 1.00)³  **Male (5-17 y),  incidence:**  0.91 (0.85 to 0.97)³  **Total (5-9 y),  prevalence:**  0.85 (0.73 to 1.00)³  **Total (5-9 y),  incidence:**  0.88 (0.74 to 1.06)³  **Female (5-9 y), prevalence:**  0.81 (0.64 to 1.02)³  **Female (5-9 y),  incidence:**  0.85 (0.65 to 1.11)³  **Male (5-9 y),  prevalence:**  0.89 (0.72 to 1.11)³  **Male (5-9 y),  incidence:**  0.91 (0.72 to 1.17)³ | **Moderate** |
|  |  |  |  | **Total (10-14 y), prevalence:**  2,349/212,447  **Total (10-14 y), incidence:**  1,626/212,447  **Female (10-14 y), prevalence:**  1,497/102,980  **Female (10-14 y), incidence:**  1,086/102,980  **Male (10-14 y), prevalence:**  852/109,467  **Male (10-14 y), incidence:**  540/109,467  **Total (15-17 y), prevalence:**  5,939/133,125  **Total (15-17 y), incidence:**  3,499/133,125  **Female (15-17 y), prevalence:**  4,384/64,710  **Female (15-17 y), incidence:**  2,566/64,710  **Male (15-17 y), prevalence:**  1,555/68,415  **Male (15-17 y), incidence:**  933/68,415 | **Total (10-14 y), prevalence:**  2,343/208,420  **Total (10-14 y), incidence:**  1,639/208,420  **Female (10-14 y), prevalence:**  1,416/100,972  **Female (10-14 y), incidence:**  1,024/100,972  **Male (10-14 y), prevalence:**  927/107,448  **Male (10-14 y), incidence:**  615/107,448  **Total (15-17 y), prevalence:**  5,625/131,161  **Total (15-17 y), incidence:**  3,194/131,161  **Female (15-17 y), prevalence:**  4,067/63,991  **Female (15-17 y), incidence:**  2,226/63,991  **Male (15-17 y), prevalence:**  1,558/67,170  **Male (15-17 y), incidence:**  968/67,170 |  | **Total (10-14 y), prevalence:**  0.98 (0.93 to 1.04)³  **Total (10-14 y),  incidence:**  0.97 (0.91 to 1.04)³  **Female (10-14 y), prevalence:**  1.04 (0.96 to 1.12)³  **Female (10-14 y), incidence:**  1.04 (0.95 to 1.13)³  **Male (10-14 y), prevalence:**  0.90 (0.82 to 0.99)³  **Male (10-14 y),  incidence:**  0.86 (0.77 to 0.97)³  **Total (15-17 y), prevalence:**  1.04 (1.00 to 1.08)³  **Total (15-17 y),  incidence:**  1.08 (1.03 to 1.14)³  **Female (15-17 y), prevalence:**  1.07 (1.02 to 1.12)³  **Female (15-17 y), incidence:**  1.15 (1.08 to 1.21)³  **Male (15-17 y), prevalence:**  0.98 (0.91 to 1.05)³  **Male (15-17 y),  incidence:**  0.95 (0.86 to 1.04)³ |  |
|  |  |  |  | **Total (10-17 y), stationary care DP1:**  1,316/339,361  **Female (10-17 y), stationary care DP1:**  1,038/164,829  **Male (10-17 y), stationary care DP1:**  278/174,532  **Total (10-14 y), stationary care DP1:**  372/209,495  **Female (10-14 y), stationary care DP1:**  290/101,584  **Male (10-14 y), stationary care DP1:**  82/107,911  **Total (15-17 y), stationary care DP1:**  944/129,866  **Female (15-17 y), stationary care DP1:**  748/63,245  **Male (15-17 y), stationary care DP1:**  196/66,621  **Total (10-17 y), stationary care DP2:**  1,684/343,642  **Female (10-17 y), stationary care DP2:**  1,388/166,707  **Male (10-17 y), stationary care DP2:**  296/176,935  **Total (10-14 y), stationary care DP2:**  479/211,881  **Female (10-14 y), stationary care DP2:**  385/102,804  **Male (10-14 y), stationary care DP2:**  94/109,077  **Total (15-17 y), stationary care DP2:**  1,205/131,761  **Female (15-17 y), stationary care DP2:**  1,003/63,903  **Male (15-17 y), stationary care DP2:**  202/67,858 | **Total (10-17 y), stationary care:**  1,342/332,945  **Female (10-17 y), stationary care:**  1,000/161,957  **Male (10-17 y), stationary care:**  342/170,988  **Total (10-14 y), stationary care:**  397/205,399  **Female (10-14 y), stationary care:**  311/99,551  **Male (10-14 y), stationary care:**  86/105,848  **Total (15-17 y), stationary care:**  945/127,546  **Female (15-17 y), stationary care:**  689/62,406  **Male (15-17 y), stationary care:**  256/65,140  **Total (10-17 y), stationary care:**  1,342/332,945  **Female (10-17 y), stationary care:**  1,000/161,957  **Male (10-17 y), stationary care DP2**  342/170,988  **Total (10-14 y), stationary care:**  397/205,399  **Female (10-14 y), stationary care:**  311/99,551  **Male (10-14 y), stationary care:**  86/105,848  **Total (15-17 y), stationary care:**  945/127,546  **Female (15-17 y), stationary care:**  689/62,406  **Male (15-17 y), stationary care:**  256/65,140 |  | **Total (10-17 y), stationary care PP -> DP1:**  0.96 (0.89 to 1.04)³  **Female (10-17 y), stationary care PP -> DP1:**  1.02 (0.93 to 1.11)³  **Male (10-17 y), stationary care PP -> DP1:**  0.80 (0.68 to 0.93)³  **Total (10-14 y), stationary care PP -> DP1:**  0.92 (0.80 to 1.06)³  **Female (10-14 y), stationary care PP -> DP1:**  0.91 (0.78 to 1.07)³  **Male (10-14 y), stationary care PP -> DP1:**  0.94 (0.69 to 1.27)³  **Total (15-17 y), stationary care PP -> DP1:**  0.98 (0.90 to 1.07)³  **Female (15-17 y), stationary care PP -> DP1:**  1.07 (0.97 to 1.19)³  **Male (15-17 y), stationary care PP -> DP1:**  0.75 (0.62 to 0.90)³  **Total (10-17 y), stationary care PP -> DP2:**  1.22 (1.13 to 1.31)³  **Female (10-17 y), stationary care PP -> DP2:**  1.35 (1.25 to 1.47)³  **Male (10-17 y), stationary care PP -> DP2:**  0.84 (0.72 to 0.98)³  **Total (10-14 y), stationary care PP -> DP2:**  1.17 (1.02 to 1.34)³  **Female (10-14 y), stationary care PP -> DP2:**  1.20 (1.03 to 1.39)³  **Male (10-14 y), stationary care PP -> DP2:**  1.06 (0.79 to 1.42)³  **Total (15-17 y), stationary care PP -> DP2:**  1.24 (1.14 to 1.35)³  **Female (15-17 y), stationary care PP -> DP2:**  1.43 (1.30 to 1.57)³  **Male (15-17 y), stationary care PP -> DP2:**  0.76 (0.63 to 0.91)³ |  |
| Bujard, 2021 [4] | German adaption of State-Trait Depression Scale (STDS)  Subgroups:   - Gender - Age   Psychometric properties [11]; | No adjustment | General depression symptoms  *Additional data were provided by authors* | **Total (16-19y):**  0.25 (0.48) 854  **Female (16-19y):**  0.36 (0.53) 493  **Male (16-19y):**  0.15 (0.62) 361 | **Total (16-19y):**  0.10 (0.33) 854  **Female (16-19y):**  0.13 (0.38) 493  **Male (16-19y):**  0.07 (0.42) 361 | **Total (16-19y):**  0.36 (0.26 to 0.46)³  **Female (16-19y):**  0.48 (0.35 to 0.61)³  **Male (16-19y):**  0.15 (0.01 to 0.29)³ |  | **Mode-rate** |
|  |  |  | Clinically relevant depression symptoms,  clinical cut off ≥25 STDS score | **Total (16-19y):**  215/854  **Female (16-19y):**  175/493  **Male (16-19y):**  55/361 | **Total (16-19y):**  87/854  **Female (16-19y):**  66/493  **Male (16-19y):**  26/361 |  | **Total (16-19y):**  2.97 (2.26 to 3.89)^1^  **Female (16-19y):**  3.56 (2.59 to 4.89)^1^  **Male (16-19y):**  2.32 (1.42 to 3.79)^1^ |  |
|  | Subsamples:   - Gender - Migration background - Mother’s education - Employment - Residence - Mental Health Composite Scale - Physical Health Composite Scale | Accordingly adjusted for gender, migration background, mother’s education, employment, residence, mental health composite scale, physical health composite scale |  |  |  |  | **Female:**  2.95 (1.85 to 4.70)^2^  **Migration background:**  1.88 (1.18 to 3.00)^2^  **Mother’s education high:**   - 1. (0.64 to 1.50)^2^   **Vocational training:**   - 1. (0.49 to 2.31)^2^   **Schoolchildren:**  1.41 (0.69 to 2.87)^2^  **Students:**  1.55 (0.48 to 5.03)^2^  **Living with parents:**  0.32 (0.13 to 0.85)^2^  **Rural housing area:**  1.11 (0.72 to 1.71)^2^  **Residence East Germany:**  0.74 (0.41 to 1.33)^2^  **Mental Health Composite Scale:**  0.97 (0.95 to 1.00)^2^  **Physical Health Composite Scale:**  0.97 (0.95 to 1.00)^2^ |  |
| Kostev,  2021 [12] | Medical classification (depression [ICD-10: F32, F33])  Subgroups:   - Gender - Age   NI for psychometric properties (International Statistical Classification of Diseases and Related Health Problems [ICD]) | No adjustment | Clinically relevant depression symptoms  *Additional data were provided by authors* | **Total (2-17 y), prevalence:**  1,596/203,742  **Total (2-17 y), incidence:**  1,168/203,742  **Female (2-17 y), prevalence:**  1,041/80,964  **Female (2-17 y), incidence:**  754/80,964  **Male (2-17 y), prevalence:**  555/122,778  **Male (2-17 y), incidence:**  414/122,778  **Total (2-5 y), prevalence:**  16/107,629  **Total (2-5 y), incidence:**  16/107,629  **Female (2-5 y), prevalence:**  5/46,024  **Female (2-5 y), incidence:**  5/46,024  **Male (2-5 y), prevalence:**  11/61,605  **Male (2-5 y), incidence:**  11/61,605 | **Total (2-17 y), prevalence:**  1,426/206,528  **Total (2-17 y), incidence:**  986/206,528  **Female (2-17 y), prevalence:**  878/80,934  **Female (2-17 y), incidence:**  584/80,934  **Male (2-17 y), prevalence:**  548/125,594  **Male (2-17 y), incidence:**  402/125,594  **Total (2-5 y), prevalence:**  12/108,204  **Total (2-5 y), incidence:**  11/108,204  **Female (2-5 y), prevalence:**  3/46,313  **Female (2-5 y), incidence:**  3/46,313  **Male (2-5 y), prevalence:**  9/61,891  **Male (2-5 y), incidence:**  8/61,891 |  | **Total (2-17 y),  prevalence:**  1.14 (1.06 to 1.22)³  **Total (2-17 y),  incidence:**  1.20 (1.10 to 1.31)³  **Female (2-17 y), prevalence:**  1.19 (1.09 to 1.30)³  **Female (2-17 y), incidence:**  1.29 (1.16 to 1.44)³  **Male (2-17 y),  prevalence:**  1.04 (0.92 to 1.17)³  **Male (2-17 y),  incidence:**  1.05 (0.92 to 1.21)³  **Total (2-5 y),  prevalence:**  1.34 (0.63 to 2.83)³  **Total (2-5 y),  incidence:**  1.46 (0.68 to 3.15)³  **Female (2-5 y), prevalence:**  1.68 (0.40 to 7.02)³  **Female (2-5 y),  incidence:**  1.68 (0.40 to 7.02)³  **Male (2-5 y),  prevalence:**  1.23 (0.51 to 2.96)³  **Male (2-5 y),  incidence:**  1.38 (0.56 to 3.43)³ | **Mode-rate** |
|  |  |  |  | **Total (6-9 y), prevalence:**  102/44,545  **Total (6-9 y), incidence:**  89/44,545  **Female (6-9 y), prevalence:**  39/15,378  **Female (6-9 y), incidence:**  36/15,378  **Male (6-9 y), prevalence:**  63/29,167  **Male (6-9 y), incidence:**  53/29,167  **Total (10-12 y), prevalence:**  273/23,986  **Total (10-12 y), incidence:**  227/23,986  **Female (10-12 y), prevalence:**  151/7,908  **Female (10-12 y), incidence:**  126/7,908  **Male (10-12 y), prevalence:**  122/16,078  **Male (10-12 y), incidence:**  101/16,078 | **Total (6-9 y), prevalence:**  102/45,570  **Total (6-9 y), incidence:**  88/45,570  **Female (6-9 y), prevalence:**  39/15,659  **Female (6-9 y), incidence:**  37/15,378  **Male (6-9 y), prevalence:**  63/29,911  **Male (6-9 y), incidence:**  51/29,911  **Total (10-12 y), prevalence:**  212/24,797  **Total (10-12 y), incidence:**  168/24,797  **Female (10-12 y), prevalence:**  109/8,046  **Female (10-12 y), incidence:**  87/8,046  **Male (10-12 y), prevalence:**  103/16,751  **Male (10-12 y), incidence:**  81/16,751 |  | **Total (6-9 y),  prevalence:**  1.02 (0.78 to 1.35)³  **Total (6-9 y),  incidence:**  1.03 (0.77 to 1.39)³  **Female (6-9 y), prevalence:**  1.02 (0.65 to 1.59)³  **Female (6-9 y),  incidence:**  0.99(0.63 to 1.57)³  **Male (6-9 y),  prevalence:**  1.03 (0.72 to 1.45)³  **Male (6-9 y),  incidence:**  1.07 (0.73 to 1.57)³  **Total (10-12 y), prevalence:**  1.34 (1.11 to 1.60)³  **Total (10-12 y),  incidence:**  1.40 (1.15 to 1.71)³  **Female (10-12 y), prevalence:**  1.42 (1.11 to 1.82)³  **Female (10-12 y), incidence:**  1.48 (1.13 to 1.95)³  **Male (10-12 y), prevalence:**  1.24 (0.95 to 1.61)³  **Male (10-12 y),  incidence:**  1.30 (0.97 to 1.74)³ |  |
|  |  |  |  | **Total (13-17 y), prevalence:**  1,205/27,582  **Total (13-17 y), incidence:**  836/27,582  **Female (13-17 y), prevalence:**  846/11,654  **Female (13-17 y), incidence:**  587/11,654  **Male (13-17 y), prevalence:**  359/15,928  **Male (13-17 y), incidence:**  249/15,928 | **Total (13-17 y), prevalence:**  1,100/27,957  **Total (13-17 y), incidence:**  719/27,957  **Female (13-17 y), prevalence:**  727/10,916  **Female (13-17 y), incidence:**  457/10,916  **Male (13-17 y), prevalence:**  373/17,041  **Male (13-17 y), incidence:**  262/17,041 |  | **Total (13-17 y), prevalence:**  1.12 (1.03 to 1.21)³  **Total (13-17 y),  incidence:**  1.18 (1.07 to 1.31)³  **Female (13-17 y), prevalence:**  1.10 (0.99 to 1.22)³  **Female (13-17 y), incidence:**  1.21 (1.07 to 1.38)³  **Male (13-17 y), prevalence:**  1.03 (0.89 to 1.19)³  **Male (13-17 y),  incidence:**  1.02 (0.85 to 1.21)³ |  |
| Rau, 2021 [13] | Revised Child Anxiety and Depression Scale (RCADS)  Subgroups:   - Gender   Psychometric properties [14]; | No adjustment | General depression symptoms  *Additional data were provided by authors* | **Total (9-17 y):**  5.41 (5.35) 777  **Female (9-17 y):**  7.06 (5.91) 414  **Male (9-17 y):**  3.52 (3.84) 363 | **Total (9-17 y):**  PP1:  6.27 (5.30) 777  PP2:  6.70 (5.93) 777  **Female (9-17 y):**  PP1: 7.80 (5.91) 414  PP2: 8.36 (6.46) 414  **Male (9-17 y):**  PP1: 4.52 (3.82) 363  PP2: 4.81 (4.59) 363 | **Total (9-17 y):**  PP1 -> DP:  -0.16 (-0.26 to -0.06)^1^  PP2 -> DP:  -0.23 (-0.33 to -0.13)^1^  **Female (9-17 y):**  PP1 -> DP:  -0.13 (-0.26 to 0.01)³  PP2 -> DP:  -0.21 (-0.35 to -0.07)³  **Male (9-17 y):**  PP1 -> DP:  -0.26 (-0.41 to -0.11)^3^  PP2 -> DP:  -0.30 (-0.45 to -0.15)^3^ |  | **Serious** |
|  |  |  | Clinically relevant depression symptoms,  clinical cut-off:  T-Score>65 | **Total (9-17 y):**  60/777  **Female (9-17 y):**  51/414  **Male (9-17 y):**  9/363 | **Total (9-17 y):**  PP1: 71/777  PP2: 94/777  **Female (9-17 y):**  PP1: 59/414  PP2: 73/414  **Male (9-17 y):**  PP1: 12/363  PP2: 21/363 |  | **Total (9-17 y):**  PP1 -> DP:  0.83 (0.58 to 1.19)³  PP2 -> DP:  0.61 (0.43 to 0.85)³  **Female (9-17 y):**  PP1 -> DP:  0.85 (0.57 to 1.26)³  PP2 -> DP:  0.66 (0.45 to 0.97)³  **Male (9-17 y):**  PP1 -> DP:  0.74 (0.31 to 1.79)^3^  PP2 -> DP:  0.41 (0.19 to 0.92)³ |  |

| **Iceland** |  |  |  |  |  |  |  |  |
| --- | --- | --- | --- | --- | --- | --- | --- | --- |
| Thorisdottir, 2021 [15] | Symptom Checklist‐Revised (SCL‐90)  Subgroups:   - Age   Psychometric properties [16]; | No adjustment  Adjustment for gender and household status | General depression symptoms  *Additional data were requested from the authors; no data were provided* | **Total (13-18 y):**  20.30 (7.98) 15,725  **Total (13-15 y):**  19.0 (7.52) 8,170  **Total (16-18 y):**  21.77 (8.23) 7,555 | **Total (13-18 y):**  PP1: 17.96 (7.53) 19,682  PP2:  18.54 (7.52) 18,126  **Total (13-15 y):**  PP1: 17.21 (7.44) 9,714  PP2:  17.53 (7.29) 9,077  **Total (16-18 y):**  PP1: 18.67 (7.54) 9,968  PP2:  19.45 (7.6) 9,049 | **Total (13-18 y):**  PP1 -> DP: 0.30 (0.28 to 0.32)^1^  PP2 -> DP: 0.23 (0.21 to 0.25)^1^  **Total (13-15 y):**  PP1 -> DP: 0.24 (0.21 to 0.27)^1^  PP2 -> DP: 0.20 (0.17 to 0.23)^1^  **Total (16-18 y):**  PP1 -> DP: 0.40 (0.36 to 0.43)^1^ PP2 -> DP: 0.29 (0.26 to 0.32)^1^ |  | **Moderate** |
| Halldors-dottir, 2021 [17] | Symptom Checklist‐Revised (SCL‐90)  Subgroups:   - Gender   Psychometric properties [16]; | No adjustment | General depression symptoms  *Additional data were requested from the authors; no data were provided* | **Female (16-17 y):**  13.49 (8.91) 287  **Male (16-17 y):**  6.46 (7.03) 217 | **Female (16-17 y):**  10.99 (7.70) 287  **Male (16-17 y):**  7.16 (6.91) 217 | **Female (16-17 y):**  0.30 (0.14 to 0.46)^1^  **Male (16-17 y):**  -0.10 (-0.29 to 0.09)^1^ |  | **Serious** |
| **Israel** |  |  |  |  |  |  |  |  |
| Shoshani, 2021 [18] | Brief Symptom Inventory 18 (BSI-18), subscale depression  NI for psychometric properties; | No adjustment | General depression symptoms  *Additional data were requested from the authors; no data were provided* | **Total (11-17 y):**  7.59 (5.25) 1,537 | **Total (11-17 y):**  6.14 (4.73) 1,537 | **Total (11-17 y):**  0.29 (0.22 to 0.36)^1^ |  | **Moderate** |

| **Italy** |  |  |  |  |  |  |  |  |
| --- | --- | --- | --- | --- | --- | --- | --- | --- |
| Frigerio, 2022 [19] | Child Behavior Checklist (CBCL 1½-5), subscale anxious/ depressed  Subgroups:   - Gender   Psychometric properties[20] | No adjustment | General depression symptoms  *Additional data were not usable* | **Total (1-5 y):**  2.85 (2.22) 59  **Female (1-5 y):**  2.33 (1.96) 27  **Male (1-5 y):**  3.28 (2.36) 32 | **Total (1-5 y):**  PP1:  1.83 (1.60) 94  PP2:  1.89 (1.74) 88  **Female (1-5 y):**  PP1:  1.75 (1.70) 44  PP2:  1.54 (1.85) 41  **Male (1-5 y):**  PP1:  1.90 (1.51) 50  PP2:  2.19 (1.61) 47 | **Total (1-5 y):**  PP1 -> DP:  0.54 (0.21 to 0.88)^1^  PP2 -> DP:  0.49 (0.16 to 0.83)^1^  **Female (1-5 y):**  PP1 -> DP:  0.32 (-0.16 to 0.80)^1^  PP2 -> DP:  0.41 (-0.08 to 0.90)^1^  **Male (1-5 y):**  PP1 -> DP:  0.73 (0.27 to 1.18)^1^  PP2 -> DP:  0.55 (0.10 to 1.01)^1^ |  | **Serious** |

| Crescentini, 2020 [21] | Child Behavior Checklist (CBCL 6-18), subscale withdrawn/ depressed  Subgroups:   - Gender - Age   Psychometric properties [22] | No adjustment | General depression symptoms  *Additional data were provided by authors* | **Total (6-18 y):**  2.81 (2.84) 721  **Female (6-18 y):**  2.69 (2.99) 349  **Male (6-18 y):**  2.93 (2.68) 372  **Total (6-10y):**  2.48 (2.57) 419  **Female (6-10y):**  2.31 (2.53) 197  **Male (6-10y):**  2.63 (2.59) 222  **Total (11-15y):**  3.27 (3.18) 288  **Female (11-15y):**  3.20 (3.47) 148  **Male (11-15y):**  3.35 (2.80) 140  **Total (16-18y):**  3.36 (1.99) 14  **Female (16-18y):**  2.75 (1.48) 4  **Male (16-18y):**  3.60 (2.11) 10 | **Total (6-18 y):**  2.23 (2.62) 721  **Female (6-18 y):**  2.13 (2.71) 349  **Male (6-18 y):**  2.33 (2.52) 372  **Total (6-10y):**  1.83 (2.20) 419  **Female (6-10y):**  1.75 (2.29) 197  **Male (6-10y):**  1.90 (2.11) 222  **Total (11-15y):**  2.80 (3.07) 288  **Female (11-15y):**  2.64 (3.15) 148  **Male (11-15y):**  2.97 (2.96) 140  **Total (16-18y):**  2.50 (2.03) 14  **Female (16-18y):**  1.50 (0.50) 4  **Male (16-18y):**  2.90 (2.26) 10 | **Total (6-18 y):**  0.21 (0.11 to 0.31)^1^  **Female (6-18 y):**  0.20 (0.05 to 0.35)³  **Male (6-18 y):**  0.23 (0.09 to 0.37)³  **Total (6-10y):**  0.27 (0.14 to 0.41)^3^  **Female (6-10y):**  0.23 (0.03 to 0.43)^3^  **Male (6-10y):**  0.31 (0.12 to 0.50)^3^  **Total (11-15y):**  0.15 (-0.01 to 0.31)^3^  **Female (11-15y):**  0.17 (-0.06 to 0.40)^3^  **Male (11-15y):**  0.13 (-0.10 to 0.37)^3^  **Total (16-18y):**  0.42 (-0.33 to 1.17)^3^  **Female (16-18y):**  0.98 (-0.56 to 2.52)^3^  **Male (16-18y):**  0.31 (-0.58 to 1.20)^3^ |  | **Critical** |
| --- | --- | --- | --- | --- | --- | --- | --- | --- |

| **Netherlands** | |  |  |  |  |  |  |  |
| --- | --- | --- | --- | --- | --- | --- | --- | --- |
| Luijten, 2021 [23] | Patient-Reported Outcome Measurement Information System (PROMIS), CAT V2.0-Depressive Symptoms  Subgroups:   - Gender - Age   Psychometric properties [24] | No adjustment | General depression symptoms  *Additional data were provided by authors* | **Total (8-18 y):**  49.4 (8.0) 813  **Female (8-18 y):**  49.7 (8.1) 444  **Male (8-18 y):**  49.1 (8.1) 400  **Total (8-10 y):**  50.3 (8.2) 195  **Female (8-10 y):**  50.5 (8.4) 111  **Male (8-10 y):**  50.1 (8.0) 84  **Total (11-15 y):**  49.1 (8.1) 431  **Female (11-15 y):**  49.2 (7.9) 224  **Male (11-15 y):**  49.0 (8.2) 207  **Total (16-18 y):**  49.0 (7.8) 189  **Female (16-18 y):**  50.1 (7.9) 99  **Male (16-18 y):**  47.8 (7.5) 90 | **Total (8-18 y):**  44.7 (10.6) 1,318  **Female (8-18 y):**  45.7 (11.0) 667  **Male (8-18 y):**  43.8 (10.1) 652  **Total (8-10 y):**  44.7 (9.8) 394  **Female (8-10 y):**  44.2 (9.9) 204  **Male (8-10 y):**  45.2 (9.7) 190  **Total (11-15 y):**  44.5 (10.6) 618  **Female (11-15 y):**  45.3 (11.0) 289  **Male (11-15 y):**  43.8 (10.3) 329  **Total (16-18 y):**  45.3 (11.6) 307  **Female (16-18 y):**  48.0 (12.1) 174  **Male (16-18 y):**  41.8 (9.9) 133 | **Total (8-18 y):**  0.48 (0.40 to 0.56)^1^  **Female (8-18 y):**  0.40 (0.28 to 0.52)^3^  **Male (8-18 y):**  0.56 (0.44 to 0.68)^3^  **Total (8-10 y):**  0.60 (0.43 to 0.78)^3^  **Female (8-10 y):**  0.67 (0.43 to 0.91)^3^  **Male (8-10 y):**  0.53 (0.27 to 0.79)^3^  **Total (11-15 y):**  0.48 (0.35 to 0.60)^3^  **Female (11-15 y):**  0.40 (0.22 to 0.58)^3^  **Male (11-15 y):**  0.54 (0.37 to 0.71)^3^  **Total (16-18 y):**  0.36 (0.18 to 0.54)^3^  **Female (16-18 y):**  0.19 (-0.05 to 0.43)^3^  **Male (16-18 y):**  0.66 (0.39 to 0.93)^3^ |  | **Moderate** |
|  |  |  | Cut off > 1.5 SD on PROMIS domains | **Participants with poor functioning or severe symptoms** 58/813 | **Participants with poor functioning or severe symptoms** 108/1,318 |  | **Participants with poor functioning or severe symptoms** 0.86 (0.62 to 1.20)^1^ |  |
| Janssen, 2020 [25] | Patient health questionnaire (PHQ-9)  Subgroups:   - Gender - Age   Psychometric properties [26] | No adjustment | General depression symptoms  *Additional data were provided by authors* | **Total (11-17 y):**  4.82 (3.42) 34  **Total/Female/Male  (11-15 y):**  No data  **Total (16-17 y):**  5.35(3.50) 20  **Female (16-17 y):**  6.54 (3.64) 13  **Male (16-17y):**  3.14 (1.86) 7 | **Total (11-17 y):**  4.21 (2.54) 34  **Total (11-15 y):**  4.00 (2.74) 5  **Female (11-15y):**  3.67 (2.89) 3  **Male (11-15y):**  4.50 (3.54) 2  **Total (16-17 y):**  4.09(2.57) 23  **Female (16-17 y):**  4.31 (2.63) 15  **Male (16-17y):**  3.13 (2.7) 8 | **Total (11-17 y):**  0.20 (-0.28 to 0.68)^1^  **Total (16-17 y):**  0.41 (-0.20 to 1.02)^3^  **Female (16-17 y):**  0.69 (-0.08 to 1.46)^3^  **Male (16-17 y):**  0.00 (-1.01 to 1.0)^3^ |  | **Serious** |

| **Norway** |  |  |  |  |  |  |  |  |
| --- | --- | --- | --- | --- | --- | --- | --- | --- |
| Soest, 2022 [27] | Kandel and Davies’s six-item Depressive Mood Inventory  Subgroups:   - Gender - Age   NI for psychometric properties | No adjustment | General depression symptoms  *Additional data were provided by authors* | **Total (13-18 y):**  37.97 (26.68) 83,670  **Female (13-18 y):**  46.51 (26.35) 42,993  **Male (13-18 y):**  28.94 (23.92) 40,667  **Total (13-15 y):**  36.19 (26.83) 45,923  **Female (13-15 y):**  45.26 (26.92) 23,030  **Male (13-15 y):**  27.07 (23.42) 22,893  **Total (16-18 y):**  40.13 (26.35) 37,747  **Female (16-18 y):**  47.95 (25.60) 19,963  **Male (16-18 y):**  31.35 (24.34) 17,784  **Family poverty high (13-18y):**  59.23 (27.61) 3,353  **Family poverty low (13-18y):**  37.08 (26.27) 80,317  **Family poverty high (13-15y):**  59.14 (28.56) 1,443  **Family poverty low (13-15y):**  35.45 (26.44) 44,480  **Family poverty high (16-18y):**  59.30 (26.88) 1,910  **Family poverty low (16-18y):**  39.11 (25.92) 35,837 | PP7  **Total (13-18 y):**  35.39 (25.71) 10,151  **Female (13-18 y):**  43.08 (25.56) 5,192  **Male (13-18 y):**  27.34 (23.27) 4,959  **Total (13-15 y):**  32.02 (25.48) 4,075  **Female (13-15 y):**  39.84 (26.05) 2,032  **Male (13-15 y):**  24.25 (22.34) 2,043  **Total (16-18 y):**  37.65 (25.61) 6,076  **Female (16-18 y):**  45.17 (25.03) 3,160  **Male (16-18 y):**  29.50 (23.67) 2,916  **Family poverty high (13-18y):**  54.52 (26.24) 547  **Family poverty low (13-18y):**  34.30 (25.24) 9,604  **Family poverty high (13-15y):**  49.60 (25.83) 154  **Family poverty low (13-15y):**  31.33 (25.22) 3,921  **Family poverty high (16-18y):**  56.44 (26.18) 393  **Family poverty low (16-18y):**  36.35 (25.06) 5,683 | PP7 -> DP:  **Total (13-18 y):**  0.10 (0.08 to 0.12)^1^  **Female (13-18 y):**  0.13 (0.10 to 0.16)^3^  **Male (13-18 y):**  0.07 (0.04 to 0.10)^3^  **Total (13-15 y):**  0.16 (0.12 to 0.20)^3^  **Female (13-15 y):**  0.20 (0.16 to 0.24)^3^  **Male (13-15 y):**  0.12 (0.08 to 0.16)^3^  **Total (16-18 y):**  0.09 (0.07 to 0.11)^3^  **Female (16-18 y):**  0.11 (0.07 to 0.15)^3^  **Male (16-18 y):**  0.08 (0.04 to 0.12)^3^  **Family poverty high  (13-18y):**  0.17 (0.08 to 0.26)^3^  **Family poverty low  (13-18y):**  0.11 (0.09 to 0.13)^3^  **Family poverty high  (13-15y):**  0.34 (0.17 to 0.50)^3^  **Family poverty low  (13-15y):**  0.16 (0.12 to 0.19)^3^  **Family poverty high  (16-18y):**  0.11 (-0.00 to 0.22)^3^  **Family poverty low  (16-18y):**  0.11 (0.08 to 0.13)^3^ |  | **Mode-rate** |
| Burdzovic, 2021 [28] | 9-item Patient Health Questionnaire (PHQ-9; adolescent version)  Subgroups:   - Gender   Psychometric properties [26] | Adjusted for adolescent sex, paren-tal cohabit-tation, subjective social status, immigrant background, grade 11 Negative Life Events Index and grade 10 outcomes | General depression symptoms  *Additional data were provided by authors* | **Total (15-16y):**  8.29 (5.44) 741  **Female (15-16y):**  9.77 (4.42) 443  **Male (15-16y):**  6.07 (3.97) 298  **Total (15-16y), high pandemic anxiety:**  9.5 (5.1) 158 | **Total (15-16y):**  8.14 (4.39) 1,335  **Female (15-16y):**  9.62 (3.40) 801  **Male (15-16y):**  5.92 (3.93) 534  **Total (15-16y), high pandemic anxiety:**  8.13 (4.72) 1,621 | **Total (15-16y):**  0.03 (-0.06 to 0.12)³  **Female (15-16y):**  0.04 (-0.08 to 0.16)³  **Male (15-16y):**  0.04 (-0.10 to 0.18)³  **Total (15-16y), high pandemic anxiety:**  0.29 (0.12 to 0.45)^1^ |  | **Mode-rate** |
|  |  |  | Clinically relevant depression symptoms,  clinical cut ≥15 PHQ-9 | **Total (15-16y):**  69/741  **Female (15-16y):**  67/443  **Male (15-16y):**  13/298 | **Total (15-16y):**  122/1,335  **Female (15-16y):**  119/801  **Male (15-16y):**  23/534 |  | **Total (15-16y):**  1.02 (0.75 to 1.39)³  **Female (15-16y):**  1.02 (0.74 to 1.41)^3^  **Male (15-16y):**  1.01 (0.51 to 2.03)^3^ |  |
|  |  | Subdivision into high pandemic anxiety (HPA) and low pandemic anxiety (LPA) |  | **Total (15-16y), high pandemic anxiety & clinical cut:**  29/160 | **Total (15-16y), high pandemic anxiety & clinical range:**  151/1,621 |  | **Total (15-16y), high pandemic anxiety & clinical cut:**  2.16 (1.39 to 3.33)^1^ |  |
| Myhr, 2021 [29] | Depressive symptoms in the past week (HSCL)  Subgroups:   - Gender   NI for psychometric properties | Adjusted for school grade level and socio-economic position | General depression symptoms (cut off ≥3 HSCL)  *Additional data were provided by authors* | **Total (13-16 y):**  275/1,957  **Female (13-16 y):**  201/993  **Male (13-16 y):**  74/964 | **Total (13-16 y):**  258/2,126  **Female (13-16 y):**  190/1,106  **Male (13-16 y):**  68/1,020 | ***Total (13-16 y):***  *0.14 (0.03 to 0.25)^3*^*  ***Female (13-16 y):***  *0.14 (0.02 to 0.26)^3^*  ***Male (13-16 y):***  *0.11 (-0.08 to 0.30)^3^*  **CI end was adapted* | **Total (13-16 y):**  1.28 (1.06 to 1.28)^2^  **Female (13-16 y):**  1.29 (1.03 to 1.62)²  **Male (13-16 y):**  1.23 (0.87 to 1.75)^2^ | **Mode-rate** |
| Hafstad, 2021 [30] | HSCL-10, anxiety/depression symptoms  Psychometric properties [31] | No adjustment | General depression symptoms  *Additional data were requested from the authors; no data were provided* | **Total (12-16y):**  0.57 (0.64) 3,572 (estimated)  **Total (12-16y), clinical cut:**  196/3,572 | **Total (12-16y):**  0.51 (0.62) 3,572*  (estimated)  **Total (12-16y), clinical cut:**  225/3,572*  **inconsistent reporting in original study* | **Total (12-16y):**  0.10 (0.05 to 0.14)^1^ | **Total (12-16y), clinical cut:**  0.86 (0.71 to 1.05)^1^ | **Serious** |
| **Switzerland** |  |  |  |  |  |  |  |  |
| Ertanir, 2021 [32] | Hopkins Symptoms Checklist (HSCL-25), subscale depression  Subgroups:   - Gender   NI for psychometric properties | No adjustment | General depression symptoms  *Additional data were provided by authors* | **Total (11-15y):**  1.87 (0.75) 314³  **Female (11-15y):**  2.14 (0.82) 135³  **Male (11-15y):**  1.63 (0.61) 161³ | **Total (11-15y):**  1.79 (0.67) 359³  **Female (11-15y):**  1.89 (0.75) 166³  **Male (11-15y):**  1.68 (0.58) 187³ | **Total (11-15y):**  0.11 (-0.04 to 0.26)³  **Female (11-15y):**  0.32 (0.09 to 0.55)³  **Male (11-15y):**  -0.08 (-0.29 to 0.13)³ |  | **Mode-rate** |
| Borbás, 2021 [33] | Child Behavior Checklist (CBCL 6-18), subscales anxious/ depressed  Psychometric properties[34] | No adjustment | General depression symptoms  *No further data were requested.* | **Total (7-17 y):**  55 (8.32) 26 | **Total (7-17 y):**  56.73 (8.49) 26 | **Total (7-17y):**  -0.20 (-0.75 to 0.34)^1^ |  | **Critical** |

| **United Kingdom** | |  |  |  |  |  |  |  |
| --- | --- | --- | --- | --- | --- | --- | --- | --- |
| Knowles,  2022 [35] | Depression symptoms in the past 2 weeks; Short Mood and Feelings Questionnaire (SFMQ)  Subsamples:   - Gender   Psychometric properties [36] | No adjustment | General depression symptoms (Cut off ≥12 SFMQ)  *Additional data were requested from the authors; no data were provided* | **Total (12-18 y):**  242/1,069 (weighted)  **Female (12-18y):**  175/583 (weighted)  **Male (12-18y):**  67/486 (weighted) | **Total (12-18 y):**  PP3: 232/836 (weighted)  **Female (12-18y):**  PP3: 169/449 (weighted)  **Male (12-18y):**  PP3: 63/387 (weighted) | ***Total (12-18 y):****  *PP3 -> DP: -0.15 (-0.26 to -0.04)³*  ***Female (12-18y):***  *PP3 -> DP:*  *-0.19 (-0.33 to -0.05)³*  ***Male (12-18y):***  *PP3 -> DP:*  *-0.11 (-0.31 to 0.09)³* | **Total (12-18 y):**  PP3 -> DP: 0.76 (0.62 to 0.94)^1^  **Female (12-18y):**  PP3 -> DP:  0.71 (0.55 to 0.94)^1^  **Male (12-18y):**  PP3 -> DP:  0.82 (0.57 to 1.19)^1^ | **Serious** |
| Widnall, 2022 [37] | Hospital Anxiety & Depression Scale (HADS)  Subgroups:   - Gender   Psychometric properties [38] | No adjustment | General depression symptoms  *Additional data were provided by authors* | **Total (13-15 y):**  DP1: 4.64 (3.28) 587  DP2: 4.92 (3.85) 589  **Female (13-15 y):**  DP1: 5.11 (3.48) 345  DP2: 5.60 (4.11) 352  **Male (13-15 y):**  DP1: 3.92 (2.81) 237  DP2: 3.90 (3.18) 232 | **Total (13-15 y):**  4.70 (3.34) 589  **Female (13-15 y):**  5.03 (3.49) 349  **Male (13-15 y):**  4.18 (3.06) 236 | **Total (13-15 y):**  PP -> DP1: -0.02 (-0.13 to 0.09)^3^  PP -> DP2: 0.06 (-0.05 to 0.17)^3^  **Female (13-15 y):**  PP -> DP1: 0.02 (-0.13 to 0.17)^3^  PP -> DP2: 0.15 (0.00 to 0.30)^3^  **Male (13-15 y):**  PP -> DP1: -0.09 (-0.27 to 0.09)^3^  PP -> DP2: -0.09 (-0.27 to 0.09)^3^ |  | **Serious** |
| Wright, 2021 [39] | Depression symptoms in the past 2 weeks; Short Mood and Feelings Questionnaire (SFMQ)  Subgroups:   - Gender   Psychometric properties [40] | No adjustment | General depression symptoms  *Additional data were provided by authors* | **Total (10-12 y),  self-rated:**  6.58 (6.02) 163  **Female (10-12 y),  self-rated:**  4.85 (4.71) 74  **Male (10-12 y),  self-rated:**  8.01 (6.61) 89  **Total (10-12 y), mother-rated:**  3.76 (4.27) 199  **Female (10-12 y),  mother-rated:**  3.86 (4.39) 109  **Male (10-12 y),  mother-rated:**  3.63 (4.14) 90 | **Total (10-12 y),  self-rated:**  4.80 (5.15) 187  **Female (10-12 y),  self-rated:**  5.73 (5.69) 87  **Male (10-12 y),  self-rated:**  3.74 (4.24) 100  **Total (10-12 y), mother-rated:**  2.00 (3.33) 226  **Female (10-12 y),  mother-rated:**  2.07 (3.39) 103  **Male (10-12 y),  mother-rated:**  1.94 (3.30) 123 | **Total (10-12 y),  self-rated:**  0.32 (0.11 to 0.53)^3^  **Female (10-12 y),  self-rated:**  -0.17 (-0.48 to 0.14)^3^  **Male (10-12 y),  self-rated:**  0.78 (0.48 to 1.08)^3^  **Total (10-12 y),  mother-rated:**  0.46 (0.27 to 0.66)^3^  **Female (10-12 y),  mother-rated:**  0.45 (0.18 to 0.73)^3^  **Male (10-12 y),  mother-rated:**  0.46 (0.18 to 0.73)^3^ |  | **Serious** |
| Bignardi, 2020 [41] | Revised Child Anxiety and Depression Scale (RCADS)-short form (Lab cohort)  Subgroups:   - Gender   Psychometric properties [14] | No adjustment  Adjustment for responder, SES, lockdown* SES | General depression symptoms  *Additional data were provided by authors* | **Total (7-12y), parent-reported:**  -0.14 (0.94) 54  **Female (7-12y), parent-reported:**  -0.25 (1.03) 32  **Male (7-12y), parent-reported:**  -0.03 (0.83) 22 | **Total (7-12y), parent-reported:**  -0.90 (0.79) 51  **Female (7-12y), parent-reported:**  -0.95 (0.72) 31  **Male (7-12y), parent-reported:**  -0.82 (0.89) 20 | **Total (7-12y), parent-reported:**  0.87 (0.47 to 1.27)³  **Female (7-12y), parent-reported:**  0.78 (0.26 to 1.30)³  **Male (7-12y), parent-reported:**  0.90 (0.26 to 1.54)³  **Total (7-12y):**  0.73 (0.46 to 1.00)² |  | **Serious** |

* Effect estimates in italics have been converted using appropriate conversion factors in accordance to Chinn [42].

^1^ Effect estimates were self-calculated based on the reported data.

² Effect estimates were so reported in the publication.

³ Additional data for calculation requested.

A, Anxiety; D, Depression; DP, during pandemic; ED, Emergency departments; M, mean; n, events; N, total; NI, no information; PP, pre-pandemic; SD, Standard deviation; y, years of age

# Table S8: Summary of details on risk of bias (RoB) assessment in included studies

**Germany**

| **Study: Bujard et al. 2021*** | |  |  |
| --- | --- | --- | --- |
| *Bias items* | *Risk of Bias (RoB)* | *Direction of bias* | *Rationale* |
| 1) Bias due to confounding | Moderate RoB | Overestimation of the correct estimate | No adjustment in the main analysis but stratification for gender, adjustment in the subgroup analysis, measuring of confounding variables appear to be reliable. |
| 2) Bias in selection of participants into the study | Moderate RoB | Overestimation of the correct estimate | Representative study population. Inclusion/exclusion criteria are clear described. Participation of eligible persons 29% for PP. Pre-pandemic baseline is the same population. Follow-up rate 35%. |
| 3) Bias in classification of exposures | Low RoB | Towards null | Good description of the time point of data assessment during the COVID-19 pandemic, pre-pandemic baseline of the same population. |
| 4) Bias due to deviations from intended exposures | Not applicable |  |  |
| 5) Bias due to missing data | Moderate RoB | Unknown | Data availability PP=29% and DP=35%. Use of calibrated design weight for aligning the distribution of the study sample and controlling for differences in willingness to participate. No evidence robustness prove. |
| 6) Bias in measurement of the outcome | Low RoB | Towards null | Use of same methods for the whole population sample, use of reliable and validated measurements, self-report of participants. |
| 7) Bias in selection of the reported results | Moderate RoB | Unknown | A priori analysis plan not reported. Consistence reporting, numerous subgroup results have been reported. |
| **Overall RoB** | **Moderate RoB** |  |  |

* Rating with inclusion of the publication of Naumann et al. [43].

| **Study: Kostev et al. 2021** | |  |  |
| --- | --- | --- | --- |
| *Bias items* | *Risk of Bias (RoB)* | *Direction of bias* | *Rationale* |
| 1) Bias due to confounding | Moderate RoB | Overestimation of the correct estimate | No adjustment, stratification for age and gender. Broad study population. No analysis with confounding domains. |
| 2) Bias in selection of participants into the study | Moderate RoB | Underestimation of the correct estimate | Cross-sectional study, representative for the sociodemographic situation in Germany. Inclusion of general population. Inclusion/exclusion are clear described. Participation rate NA. Pre-pandemic baseline is not the same population. Follow-up rate not applicable. Misclassification or underestimation of individual diagnoses It can be assumed that some cases could not documented (barrier physician). |
| 3) Bias in classification of exposures | Moderate RoB | Unknown | Description of the time point of data assessment during the COVID-19 pandemic. Cross-sectional study design. The database used has a coverage of approximately 3.5% for pediatric practices. |
| 4) Bias due to deviations from intended exposures | Not applicable |  |  |
| 5) Bias due to missing data | Moderate RoB | Unknown | Data availability=3.5% of German pediatric practices. No information on handling with missing variables. No evidence robustness prove. |
| 6) Bias in measurement of the outcome | Moderate RoB | Underestimation of the correct estimate | Use of same methods for the whole population sample, use of ICD-classification, physician-reported. Misclassification or underestimation of individual diagnoses could occur due to the use of the ICD-10 coding system. |
| 7) Bias in selection of the reported results | Moderate RoB | Unknown | A priori analysis plan not reported. Consistence reporting. |
| **Overall RoB** | **Moderate RoB** |  |  |

| **Study: Rau et al. 2021** | |  |  |
| --- | --- | --- | --- |
| *Bias items* | *Risk of Bias (RoB)* | *Direction of bias* | *Rationale* |
| 1) Bias due to confounding | Moderate RoB | Overestimation of the correct estimate | No stepwise modeling, further age- and gender-stratified data were provided. No analysis with confounding domains. |
| 2) Bias in selection of participants into the study | Serious RoB | Underestimation of the correct estimator | Participants were recruited at three German schools of diverse educational levels. Pre-pandemic baseline is the same population. No clear inclusion/exclusion criteria. Participation rate of eligible persons <50% (38.5%). Follow-up rate=63.3%. |
| 3) Bias in classification of exposures | Low RoB | Towards null | Description of the time point of data assessment during the COVID-19 pandemic. Pre-pandemic baseline of the same population. |
| 4) Bias due to deviations from intended exposures | Not applicable |  |  |
| 5) Bias due to missing data | Moderate RoB | Unknown | 99% and 94% complete the questionnaire at PP1 and PP2. Partial documented handling of missing data, no evidence of robustness. |
| 6) Bias in measurement of the outcome | Low RoB | Towards null | Use of same methods for the whole population sample, use of a reliable and validate measurement, self-report. |
| 7) Bias in selection of the reported results | Moderate RoB | Unknown | A priori analysis plan not reported. Consistence reporting. |
| **Overall RoB** | **Serious RoB** |  |  |

| **Study: Ravens-Sieberer et al. 2021** | |  |  |
| --- | --- | --- | --- |
| *Bias items* | *Risk of Bias (RoB)* | *Direction of bias* | *Rationale* |
| 1) Bias due to confounding | Moderate RoB | Overestimation of the correct estimate | No adjustment, no stratification. Broad study population. No analysis with confounding domains. Further age- and gender-stratified data were provided. |
| 2) Bias in selection of participants into the study | Moderate RoB | Towards null | Representative study population. Pre-pandemic baseline is not the same population, but both are representative. Participant rate=45.8% in DP1. Follow-up rate DP1->DP2 > 80% (81.2% + 337 new participants). Responder versus non-responder analysis. |
| 3) Bias in classification of exposures | Moderate RoB | Unknown | Description of the time point of data assessment during the COVID-19 pandemic, pre-pandemic baseline not of the same population. |
| 4) Bias due to deviations from intended exposures | Not applicable |  |  |
| 5) Bias due to missing data | Moderate RoB | Towards null | In a responder versus non-responder analysis no significant differences were found in sociodemographic or mental health-related variables. No evidence of robustness |
| 6) Bias in measurement of the outcome | Low RoB | Towards null | Use of same methods for the whole population sample, use of a reliable and validate measurement, self-report (11-17 years) and parent-report (7-17 years). |
| 7) Bias in selection of the reported results | Moderate RoB | Unknown | A priori analysis plan not reported. Consistence reporting. |
| **Overall RoB** | **Moderate RoB** |  |  |

| **Study: Witte et al. 2022** | |  |  |
| --- | --- | --- | --- |
| *Bias items* | *Risk of Bias (RoB)* | *Direction of bias* | *Rationale* |
| 1) Bias due to confounding | Moderate RoB | Overestimation of the correct estimate | No adjustment, stratification for age and gender. Broad study population. No analysis with confounding domains. Further age- and gender-stratified data were provided. |
| 2) Bias in selection of participants into the study | Moderate RoB | Underestimation of the correct estimator | Cross-sectional study. Inclusion of general population. Inclusion/exclusion are clear described. Participation rate NA. Pre-pandemic baseline is not the same population. Follow-up rate not applicable. It can be assumed that some cases could not documented (barrier physician). |
| 3) Bias in classification of exposures | Moderate RoB | Towards null | Description of the time point of data assessment during the COVID-19 pandemic. |
| 4) Bias due to deviations from intended exposures | Not applicable |  |  |
| 5) Bias due to missing data | Moderate RoB | Underestimation of the correct estimate | Data availability documented: report is based on data from 5.7% of all children and adolescents in Germany. No information on handling with missing variables. No evidence robustness prove. |
| 6) Bias in measurement of the outcome | Low RoB | Towards null | Use of same methods for the whole population sample, use of ICD-classification, physician-reported. Misclassification or underestimation of individual diagnoses could occur due to the use of the ICD-10 coding system. |
| 7) Bias in selection of the reported results | Moderate RoB | Unknown | A priori analysis plan not reported. Consistence reporting. |
| **Overall RoB** | **Moderate RoB** |  |  |

**Iceland**

| **Study: Halldorsdottir et al. 2021** | |  |  |
| --- | --- | --- | --- |
| *Bias items* | *Risk of Bias (RoB)* | *Direction of bias* | *Rationale* |
| 1) Bias due to confounding | Moderate RoB | Overestimation of the correct estimate | No adjustment, stratification for gender. Broad study population. No analysis with confounding domains. |
| 2) Bias in selection of participants into the study | Moderate RoB | Unknown | Cross-sectional study. All participants from the LIFECOURSE study were invited to participate. Inclusion/exclusion criteria are clear described. Participation of eligible persons=22%. Pre-pandemic baseline is the same population. Follow-up rate not applicable. |
| 3) Bias in classification of exposures | Low RoB | Towards null | Description of the time point of data assessment during the COVID-19 pandemic. Pre-pandemic baseline is not the same population. |
| 4) Bias due to deviations from intended exposures | Not applicable |  |  |
| 5) Bias due to missing data | Serious RoB | Unknown | Data availability=22%. No information on handling with missing variables. No evidence robustness proves. |
| 6) Bias in measurement of the outcome | Low RoB | Towards null | Use of same measurement methods for the whole population sample with satisfying psychometric prosperities, self-reported. |
| 7) Bias in selection of the reported results | Moderate RoB | Unknown | A priori analysis plan not reported. Consistence reporting. |
| **Overall RoB** | **Serious RoB** |  |  |

| **Study: Thorisdottir et al. 2021** | |  |  |
| --- | --- | --- | --- |
| *Bias items* | *Risk of Bias (RoB)* | *Direction of bias* | *Rationale* |
| 1) Bias due to confounding | Moderate RoB | Unknown | No adjustment, stratification for age. Broad study population. Linear mixed effects models. |
| 2) Bias in selection of participants into the study | Low RoB | Towards null | Longitudinal, population-based study. Inclusion/exclusion criteria are clear described Response rates of eligible participants ranged from 63–86%. Pre-pandemic baseline is the same population. Follow-up rate=87%. |
| 3) Bias in classification of exposures | Low RoB | Towards null | Description of the time point of data assessment during the COVID-19 pandemic. Pre-pandemic baseline is the same population. |
| 4) Bias due to deviations from intended exposures | Not applicable |  |  |
| 5) Bias due to missing data | Moderate RoB | Towards null | Data availability 63-86%. Multiple imputation was used to address missing data. No evidence robustness proves. |
| 6) Bias in measurement of the outcome | Low RoB | Towards null | Use of same measurement methods for the whole population sample with satisfying psychometric prosperities, self-reported. |
| 7) Bias in selection of the reported results | Moderate RoB | Unknown | A priori analysis plan not reported. Consistence reporting. |
| **Overall RoB** | **Moderate RoB** |  |  |

**Israel**

| **Study: Shoshani et al. 2021** | |  |  |
| --- | --- | --- | --- |
| *Bias items* | *Risk of Bias (RoB)* | *Direction of bias* | *Rationale* |
| 1) Bias due to confounding | Moderate RoB | Overestimation of the correct estimate | No adjustment, no stratification. No analysis with confounding domains. |
| 2) Bias in selection of participants into the study | Moderate RoB | Overestimation of the correct estimate | Selective study population (6 of 38 schools), schools were representative of schools nationwide in terms of regional distribution, school type, and socioeconomic status (SES), according to data available from the Israeli Ministry of Education. Inclusion/exclusion criteria are not clear described. Participation of eligible persons > 50%. Pre-pandemic baseline is the same population. Follow-up rate NA. |
| 3) Bias in classification of exposures | Low RoB | Towards null | Description of the time point of data assessment during the COVID-19 pandemic. Pre-pandemic baseline is the same population. |
| 4) Bias due to deviations from intended exposures | Not applicable |  |  |
| 5) Bias due to missing data | Moderate RoB | Towards null | Data availability >90%. Less than 3% of data were missing per item for both measurement points, and missing values were imputed using expectation maximization (EM) procedures. No evidence robustness proves. |
| 6) Bias in measurement of the outcome | Moderate RoB | Unknown | Use of same methods for the whole population sample, no information on psychometric prosperities, self-reported. |
| 7) Bias in selection of the reported results | Moderate RoB | Unknown | A priori analysis plan not reported. Consistence reporting. |
| **Overall RoB** | **Moderate RoB** |  |  |

**Italy**

| **Study: Crescentini et al. 2020** | |  |  |
| --- | --- | --- | --- |
| *Bias items* | *Risk of Bias (RoB)* | *Direction of bias* | *Rationale* |
| 1) Bias due to confounding | Moderate RoB | Overestimation of the correct estimate | No stepwise modeling, further age- and gender-stratified data were provided. No analysis with confounding domains. Further data were provided by the authors. |
| 2) Bias in selection of participants into the study | Critical RoB | Overestimation of the correct estimate | Selective recruitment of study population (recruited using word-of-mouth and through contacting school leaders and school teachers). Inclusion/exclusion criteria are not clear described. Participation of eligible persons NA. Pre-pandemic baseline is the same population. Parents were asked to fill the questionnaires twice: once referring to the current health emergency and once recalling how they and their children felt the months before COVID-19 outbreak. Follow-up rate NA. |
| 3) Bias in classification of exposures | Serious RoB | Overestimation of the correct estimate | Description of the time point of data assessment during the COVID-19 pandemic. Pre-pandemic baseline is the same population; however, pre-pandemic assessment was filled in retrospectively. |
| 4) Bias due to deviations from intended exposures | Not applicable |  |  |
| 5) Bias due to missing data | Serious RoB | Overestimation of the correct estimate | Data availability unclear. No information on handling with missing variables. No evidence robustness prove. |
| 6) Bias in measurement of the outcome | Moderate RoB | Towards null | Use of same methods for the whole population sample with satisfying psychometric prosperities; parent-reported. |
| 7) Bias in selection of the reported results | Moderate RoB | Unknown | A priori analysis plan not reported. Consistence reporting. |
| **Overall RoB** | **Critical RoB** |  |  |

| **Study: Friggerio et al. 2021** | |  |  |
| --- | --- | --- | --- |
| *Bias items* | *Risk of Bias (RoB)* | *Direction of bias* | *Rationale* |
| 1) Bias due to confounding | Moderate RoB | Unknown | No adjustment or stepwise modeling regarding the association of the COVID-19 pandemic and child’s CBCL, stratification on gender. No analysis with confounding domains. |
| 2) Bias in selection of participants into the study | Serious RoB | Unknown | No inclusion/exclusion criteria are clear described. Participation of eligible persons NA. Pre-pandemic baseline is the same population. Follow-up rate  PP1->DP=63%, PP2->DP=67%. |
| 3) Bias in classification of exposures | Moderate RoB | Unknown | Description of the time point of data assessment during the COVID-19 pandemic. No description of the pre-pandemic baseline. Same study population. |
| 4) Bias due to deviations from intended exposures | Not applicable |  |  |
| 5) Bias due to missing data | Serious RoB | Overestimation of the correct estimate | Data availability unclear. No information on handling with missing variables. No evidence robustness proves. |
| 6) Bias in measurement of the outcome | Serious RoB | Towards null | Use of same methods for the whole population sample with satisfying psychometric prosperities, no stratified reporting of depression and anxiety data, parent-reported. |
| 7) Bias in selection of the reported results | Moderate RoB | Unknown | A priori analysis plan not reported. Consistence reporting. |
| **Overall RoB** | **Serious RoB** |  |  |

**Netherlands**

| **Study: Janssen et al. 2021** | |  |  |
| --- | --- | --- | --- |
| *Bias items* | *Risk of Bias (RoB)* | *Direction of bias* | *Rationale* |
| 1) Bias due to confounding | Moderate RoB | Overestimation of the correct estimate | No adjustment or stepwise modeling regarding the association of the COVID-19 pandemic and adolescent’s PHQ, stratification on age and gender requested. Additional data were provided by authors. |
| 2) Bias in selection of participants into the study | Serious RoB | Overestimation of the correct estimate | No general population (recruitment of the participants was done via social media, advertisements, and flyers, with a specific focus on the inclusion of both parents). Gender-stratified data were provided by the authors. Inclusion/exclusion criteria are clearly described. Participation of eligible persons NA. Pre-pandemic baseline is the same population. Follow-up rate <80%. |
| 3) Bias in classification of exposures | Low RoB | Overestimation of the correct estimate | Description of the time point of data assessment during the COVID-19 pandemic. Same study population. |
| 4) Bias due to deviations from intended exposures | Not applicable |  |  |
| 5) Bias due to missing data | Serious RoB | Overestimation of the correct estimate | Data availability >95%. No information on handling with missing variables. No evidence robustness proves. |
| 6) Bias in measurement of the outcome | Low RoB | Towards null | Use of same methods for the whole population sample with satisfying psychometric prosperities self-reported. |
| 7) Bias in selection of the reported results | Moderate RoB | Towards null | A priori analysis plan not reported. Consistence reporting. |
| **Overall RoB** | **Serious RoB** |  |  |

| **Study: Luijten et al. 2021** | |  |  |
| --- | --- | --- | --- |
| *Bias items* | *Risk of Bias (RoB)* | *Direction of bias* | *Rationale* |
| 1) Bias due to confounding | Moderate RoB | Overestimation of the correct estimate | No adjustment or stepwise modeling regarding the association of the COVID-19 pandemic and adolescent’s PROMIS domains. Additional data were provided by authors. |
| 2) Bias in selection of participants into the study | Moderate RoB | Unknown | Cross-sectional study. Pre-pandemic population representative of the Dutch general population within 2.5% on most key demographics (age, gender, ethnicity, region, and educational level) compared to population numbers in 2017. During pandemic population represent a representative sample of approximately 1,000 children with similar characteristics (within 2.5% of the previously mentioned key demographics) as the before COVID-19 sample. Inclusion/exclusion criteria are not described. Participation of eligible persons not reported. Pre-pandemic baseline is not the same population. Follow-up rate not applicable. |
| 3) Bias in classification of exposures | Low RoB | Towards null | Description of the time point of data assessment during the COVID-19 pandemic. Pre-pandemic baseline is not the same population (cross-sectional study). |
| 4) Bias due to deviations from intended exposures | Not applicable |  |  |
| 5) Bias due to missing data | Moderate RoB | Unknown | Data availability >95%. No information on handling with missing variables (but missing data reported in tables). No evidence robustness proves. |
| 6) Bias in measurement of the outcome | Moderate RoB | Unknown | Use of same methods for the whole population sample with satisfying psychometric prosperities, not clear self-reported. |
| 7) Bias in selection of the reported results | Moderate RoB | Unknown | A priori analysis plan not reported. Consistence reporting. |
| **Overall RoB** | **Moderate RoB** |  |  |

**Norway**

| **Study: Burdzovic et al. 2021** | |  |  |
| --- | --- | --- | --- |
| *Bias items* | *Risk of Bias (RoB)* | *Direction of bias* | *Rationale* |
| 1) Bias due to confounding | Moderate RoB | Unknown | Stepwise modeling regarding the association of the COVID-19 pandemic and adolescent’s PHQ-9. Authors provided further data on gender. |
| 2) Bias in selection of participants into the study | Moderate RoB | Overestimation of the correct estimate | No representative sample. Inclusion/exclusion criteria are not clearly described. Participation of eligible persons >50%. Pre-pandemic baseline is the same population. Follow-up rate=56%. |
| 3) Bias in classification of exposures | Moderate RoB | Unknown | Description of the time point of data assessment during the COVID-19 pandemic. Pre-pandemic baseline is the same population. |
| 4) Bias due to deviations from intended exposures | Not applicable |  |  |
| 5) Bias due to missing data | Moderate RoB | Unknown | Data availability >95%. Missing values on all covariates were classified into the dummy unknown category and were included as such in all models. No evidence robustness proves. |
| 6) Bias in measurement of the outcome | Low RoB | Towards null | Use of same methods for the whole population sample with satisfying psychometric prosperities, self-reported. |
| 7) Bias in selection of the reported results | Moderate RoB | Towards null | A priori analysis plan not reported. Consistence reporting. |
| **Overall RoB** | **Moderate RoB** |  |  |

| **Study: Hafstad et al. 2021** | |  |  |
| --- | --- | --- | --- |
| *Bias items* | *Risk of Bias (RoB)* | *Direction of bias* | *Rationale* |
| 1) Bias due to confounding | Moderate RoB | Overestimation of the correct estimate | No stepwise modeling regarding the association of the COVID-19 pandemic and adolescent’s HSCL-10, stratification on gender and further social determinants. |
| 2) Bias in selection of participants into the study | Moderate RoB | Overestimation of the correct estimate | Representative longitudinal survey of Norwegian youths. Inclusion/exclusion criteria are not clearly described. Only 50% of the requested schools responded. Pre-pandemic baseline is the same population. Follow-up rate <80%. |
| 3) Bias in classification of exposures | Low RoB | Towards null | Description of the time point of data assessment during the COVID-19 pandemic. Pre-pandemic baseline is the same population. |
| 4) Bias due to deviations from intended exposures | Not applicable |  |  |
| 5) Bias due to missing data | Serious RoB | Unknown | Data availability ~90%. Data on population do not agree with data in Table 2. Available information on handling with missing variables. Evidence for robustness not reported. |
| 6) Bias in measurement of the outcome | Moderate RoB | Towards null | Use of same methods for the whole population sample with satisfying psychometric prosperities, self-reported. Depression and anxiety were measured in one scale. |
| 7) Bias in selection of the reported results | Moderate RoB | Unknown | A priori analysis plan not reported. Consistence reporting. Only 50% of the schools responded. |
| **Overall RoB** | **Serious RoB** |  |  |

| **Study: Myhr et al. 2021** | |  |  |
| --- | --- | --- | --- |
| *Bias items* | *Risk of Bias (RoB)* | *Direction of bias* | *Rationale* |
| 1) Bias due to confounding | Moderate RoB | Unknown | Adjustment regarding the association of the COVID-19 pandemic and adolescent’s HSCL-10, stratification on gender. |
| 2) Bias in selection of participants into the study | Moderate RoB | Overestimation of the correct estimate | Cross-sectional study. Inclusion/exclusion criteria are not clearly described. Participation rate of eligible persons=87% (during pandemic) and =97% (pre-pandemic). Pre-pandemic baseline is not the same population. Follow-up rate not applicable. |
| 3) Bias in classification of exposures | Moderate RoB | Overestimation of the correct estimate | Description of the time point of data assessment during the COVID-19 pandemic. Pre-pandemic baseline is the not same population (cross-sectional study). |
| 4) Bias due to deviations from intended exposures | Not applicable |  |  |
| 5) Bias due to missing data | Moderate RoB | Overestimation of the correct estimate | Data availability >90%. Available information on handling with missing variables (but not well described). No evidence robustness proves. |
| 6) Bias in measurement of the outcome | Moderate RoB | Towards null | Use of same methods for the whole population sample with satisfying psychometric properities, self-reported. |
| 7) Bias in selection of the reported results | Moderate RoB | Unknown | A priori analysis plan not reported. Consistence reporting. |
| **Overall RoB** | **Moderate RoB** |  |  |

| **Study: Soest et al. 2021** | |  |  |
| --- | --- | --- | --- |
| *Bias items* | *Risk of Bias (RoB)* | *Direction of bias* | *Rationale* |
| 1) Bias due to confounding | Moderate RoB | Overestimation of the correct estimate | Controlling for gender, age and parental education. |
| 2) Bias in selection of participants into the study | Moderate RoB | Overestimation of the correct estimate | Cross-sectional study Repeated cross-sectional survey data, collected from 2014 to 2021 nationwide, representative sample of Norwegian adolescents. Inclusion/exclusion criteria are clear described. Response rates varied from 77% to 85% between 2014 and 2021, except in 2020, when the response rate was 65%. Pre-pandemic baseline is not the same population. Follow-up rate not applicable. |
| 3) Bias in classification of exposures | Moderate RoB | Overestimation of the correct estimate | Description of the time point of data assessment during the COVID-19 pandemic. Pre-pandemic baseline is not the same population (cross-sectional study). |
| 4) Bias due to deviations from intended exposures | Not applicable |  |  |
| 5) Bias due to missing data | Moderate RoB | Overestimation of the correct estimate | Data availability=65% to 85%. Available information on handling with missing variables. No evidence robustness proves. |
| 6) Bias in measurement of the outcome | Moderate RoB | Towards null | Use of same methods for the whole population sample with satisfying psychometric properities, self-reported. |
| 7) Bias in selection of the reported results | Moderate RoB | Unknown | A priori analysis plan not reported. Consistence reporting. |
| **Overall RoB** | **Moderate RoB** |  |  |

**Switzerland**

| **Study: Borbas et al. 2020** | |  |  |
| --- | --- | --- | --- |
| *Bias items* | *Risk of Bias (RoB)* | *Direction of bias* | *Rationale* |
| 1) Bias due to confounding | Serious RoB | Overestimation of the correct estimate | No stepwise modeling, no stratification. |
| 2) Bias in selection of participants into the study | Critical RoB | Unpredictable | Selective and wide age-ranged study population (26 persons, aged 7-17). No inclusion/exclusion criteria are clear described. Participation of eligible persons = 68% (26 of 38 persons). Follow-up rate NA. |
| 3) Bias in classification of exposures | Moderate RoB | Towards null | Description of the time point of data assessment during the COVID-19 pandemic. Pre-pandemic baseline is the same population. |
| 4) Bias due to deviations from intended exposures | Not applicable |  |  |
| 5) Bias due to missing data | Moderate RoB | Overestimation of the correct estimate | Available data >95%. Limited information on handling with missing variables. No evidence robustness proves. |
| 6) Bias in measurement of the outcome | Moderate RoB | Unknown | Use of same methods for the whole population sample with satisfying psychometric prosperities, self-reported. |
| 7) Bias in selection of the reported results | Moderate RoB | Unknown | A priori analysis plan not reported. Consistence reporting. |
| **Overall RoB** | **Critical RoB** |  |  |

| **Study: Ertanir et al. 2021** | |  |  |
| --- | --- | --- | --- |
| *Bias items* | *Risk of Bias (RoB)* | *Direction of bias* | *Rationale* |
| 1) Bias due to confounding | Moderate RoB | Overestimation of the correct estimate | No stepwise modeling, multilevel models. Authors provided further data on gender. |
| 2) Bias in selection of participants into the study | Moderate RoB | Unpredictable | Selective study population (20 participating secondary schools were recruited in 2019 in three Germanspeaking Swiss cantons). No inclusion/exclusion criteria are described. No information on participation of eligible persons. Follow-up rate=85%. |
| 3) Bias in classification of exposures | Moderate RoB | Towards null | Description of the time point of data assessment during the COVID-19 pandemic. Pre-pandemic baseline is the same population. |
| 4) Bias due to deviations from intended exposures | Not applicable |  |  |
| 5) Bias due to missing data | Moderate RoB | Unknown | Limited information on handling with missing variables. No evidence robustness proves. |
| 6) Bias in measurement of the outcome | Moderate RoB | Unknown | Use of same methods for the whole population sample with satisfying psychometric prosperities, self-reported. |
| 7) Bias in selection of the reported results | Moderate RoB | Unknown | A priori analysis plan not reported. Consistence reporting. |
| **Overall RoB** | **Moderate RoB** |  |  |

**United Kingdom**

| **Study: Bignardi et al. 2020** | |  |  |
| --- | --- | --- | --- |
| *Bias items* | *Risk of Bias (RoB)* | *Direction of bias* | *Rationale* |
| 1) Bias due to confounding | Moderate RoB | Overestimation of the correct estimate | No stepwise modeling. Adjustment for responder, SES, Lockdown*SES, stratification upon request. Additional data were provided by authors. |
| 2) Bias in selection of participants into the study | Serious RoB | Unpredictable | Selective study population (convenience samples). No inclusion/exclusion criteria are clear described. Participation of eligible persons unclear. Pre-pandemic school-children and caregivers completed the assessment, during pandemic only caregivers completed the assessments. Follow-up rate NA. |
| 3) Bias in classification of exposures | Serious RoB | Towards null | Description of the time point of data assessment during the COVID-19 pandemic. Pre-pandemic school-children and caregivers completed the assessment, during pandemic only caregivers completed the assessments. |
| 4) Bias due to deviations from intended exposures | Not applicable |  |  |
| 5) Bias due to missing data | Serious RoB | Overestimation of the correct estimate | No information on handling with missing variables. No evidence robustness proves. |
| 6) Bias in measurement of the outcome | Serious RoB | Unknown | Use of same methods for the whole population sample, use of Revised Child Anxiety and Depression Scale (RCADS)-short form with anxiety and depression subscales. Self and caregiver reported, PP: self-reported, DP: caregiver-reported |
| 7) Bias in selection of the reported results | Moderate RoB | Towards null | A priori analysis plan not reported. Consistence reporting. |
| **Overall RoB** | **Serious RoB** |  |  |

| **Study: Knowles et al. 2022** | |  |  |
| --- | --- | --- | --- |
| *Bias items* | *Risk of Bias (RoB)* | *Direction of bias* | *Rationale* |
| 1) Bias due to confounding | Moderate RoB | Overestimation of the correct estimate | No adjustment for depression/anxiety analyses, stratification for gender. |
| 2) Bias in selection of participants into the study | Moderate RoB | Unpredictable | Selective study population (cohorts were recruited from twelve local secondary schools and are representative of secondary school pupils in the two boroughs). Inclusion/exclusion criteria are not clear described. Participation of eligible persons unclear. Same study population completed assessment pre and during pandemic. Follow-up rate=78%. |
| 3) Bias in classification of exposures | Moderate RoB | Towards null | Description of the time point of data assessment during the COVID-19 pandemic. Same study population completed assessment pre and during pandemic. |
| 4) Bias due to deviations from intended exposures | Not applicable |  |  |
| 5) Bias due to missing data | Moderate RoB | Overestimation of the correct estimate | Available data > 90%. Documented and comprehensible handling of missing data. No evidence for robustness. |
| 6) Bias in measurement of the outcome | Serious RoB | Unknown | Use of same methods for the whole population sample (“At T4, we revised the questionnaire, retaining core validated and widely used schedules on mental health and social circumstances.”). Self-reported. Likelihood of any systematic errors in measuring described as high by the authors. |
| 7) Bias in selection of the reported results | Moderate RoB | Unpredictable | A priori analysis plan not reported. Consistence reporting. |
| **Overall RoB** | **Serious** **RoB** |  |  |

| **Study: Widnall et al. 2022** | |  |  |
| --- | --- | --- | --- |
| *Bias items* | *Risk of Bias (RoB)* | *Direction of bias* | *Rationale* |
| 1) Bias due to confounding | Moderate RoB | Overestimation of the correct estimate | Multilevel models only for association between lockdown and return to school. Changes in anxiety and depression presented in a figure without adjustment. Further data were provided by the authors. |
| 2) Bias in selection of participants into the study | Serious RoB | Overestimation of the correct estimate | Selective study population (schools were invited). Unclear inclusion/exclusion criteria. Baseline mean response rate=77%. Same study population completed assessment pre and during pandemic. Follow-up rate 1=41% (1,039/2,549), follow-up rate 2=24% (603/2,549). |
| 3) Bias in classification of exposures | Moderate RoB | Unpredictable | Description of the time point of data assessment during the COVID-19 pandemic. Same study population completed assessment pre and during pandemic for DP1. For DP2 another study population complete the assessment. |
| 4) Bias due to deviations from intended exposures | Not applicable |  |  |
| 5) Bias due to missing data | Moderate RoB | Overestimation of the correct estimate | Available data > 90%. Documentation of missing data. No evidence for robustness. |
| 6) Bias in measurement of the outcome | Moderate RoB | Unknown | Use of same methods for the whole population sample with satisfying psychometric prosperities, self-reported. |
| 7) Bias in selection of the reported results | Moderate RoB | Unknown | A priori analysis plan not reported. Consistence reporting. |
| **Overall RoB** | **Serious RoB** |  |  |

| **Study: Wright et al. 2022** | |  |  |
| --- | --- | --- | --- |
| *Bias items* | *Risk of Bias (RoB)* | *Direction of bias* | *Rationale* |
| 1) Bias due to confounding | Moderate RoB | Overestimation of the correct estimate | Only mother-rated scores were adjusted for raters own PHQ depression score at time of rating, no adjustment for child-rated depression/anxiety scores. Additional data were provided by the authors. |
| 2) Bias in selection of participants into the study | Moderate RoB | Unpredictable | Selective study population. Inclusion/exclusion criteria are clear described. Participation of eligible persons unclear. Same study population completed assessment pre and during pandemic. Follow-up rate=89%. |
| 3) Bias in classification of exposures | Moderate RoB | Towards null | Moderately description of the time point of data assessment during the COVID-19 pandemic. Same study population completed assessment pre and during pandemic. |
| 4) Bias due to deviations from intended exposures | Not applicable |  |  |
| 5) Bias due to missing data | Serious RoB | Overestimation of the correct estimate | Data availability unclear. No documentation of missing data. No evidence for robustness. |
| 6) Bias in measurement of the outcome | Moderate RoB | Unknown | Use of same methods for the whole population sample, self-reported. |
| 7) Bias in selection of the reported results | Low RoB | Towards null | A priori plan exists. Consistence reporting. |
| **Overall RoB** | **Serious RoB** |  |  |

# Table S9. Evidence profile for grading evidence according to Grading of Recommendations, Assessment, Development and Evaluations (GRADE)

# Table S10. Moderator analysis for total sample with categorical moderators

| **Categorical moderators** | **k** | **Estimate (95% CI)** | **Test of moderators** | **Test for residual heterogeneity** |
| --- | --- | --- | --- | --- |
| Risk of Bias | 21 | -0·12 (-0·31 to 0·06) | p=0·18 | p<0·0001 |
| Symptom reporter | 21 | 0·59 (0·08 to 1·10) | **p=0·03** | p<0·0001 |
| Country | 21 | -0·00 (-0·04 to 0·03) | p=0·82 | p<0·0001 |
| Stringency Index | 21 | 0·00 (-0·00 to 0·01) | p=0·23 | p<0·0001 |
| School Closure Index | 21 | 0·05 (-0·06 to 0·15) | p=0·35 | p<0·0001 |
| Study design | 21 | 0·04 (-0·18 to 0·26) | p=0·71 | p<0·0001 |

# Table S11. Moderator analysis for total sample of low risk of bias studies with categorical moderators

| **Categorical moderators** | **k** | **Estimate (95% CI)** | **Test of moderators** | **Test for residual heterogeneity** |
| --- | --- | --- | --- | --- |
| Risk of Bias | 11 | not applicable |  |  |
| Symptom reporter | 11 | not applicable |  |  |
| Country   - - Netherlands   - Norway | 11 | 0·25 (0·08 to 0·42)  -0·14 (-0·25 to -0·02) | **p=0·0006** | p<0·0001 |
| Stringency Index | 11 | 0·16 (-0·01 to 0·34) | p=0·07 | p<0·0001 |
| School Closure Index | 11 | 0·22 (0·10 to 0·34) | **p=0·0002** | p<0·0001 |
| Study design | 11 | 0·04 (-0·13 to 0·21) | p=0·66 | p<0·0001 |

# Table S12. Moderator analysis for total sample with continuous moderators

| **Continuous moderators** | **k** | **Estimate (95% CI)** | **Test of moderators** | **Test for residual heterogeneity** |
| --- | --- | --- | --- | --- |
| Age | 21 | -0·02 (-0·07 to 0·03) | p=0·40 | p<0·0001 |
| Gender (% females) | 21 | 0·00 (-0·00 to 0·01) | p=0·22 | p<0·0001 |
| Time of measurement during pandemic | 21 | -0·02 (-0·11 to 0·07) | p=0·67 | p<0·0001 |
| Publication year | 21 | -0·13 (-0·27 to 0·01) | p=0·08 | p<0·0001 |
| Stringency Index | 21 | 0·00 (-0·00 to 0·01) | p=0·23 | p<0·0001 |
| School Closure Index | 21 | 0·05 (-0·06 to 0·15) | p=0·35 | p<0·0001 |
| Sample size | 21 | -0·00 (-0·00 to 0·00) | p=0·79 | p<0·0001 |

# Table S13. Moderator analysis for total sample of low risk of bias with continuous moderators

| **Continuous moderators** | **k** | **Estimate (95% CI)** | **Test of moderators** | **Test for residual heterogeneity** |
| --- | --- | --- | --- | --- |
| Age | 11 | 0·00 (-0·05 to 0·05) | p=0·97 | p<0·0001 |
| Gender (% females) | 11 | 0·01 (-0·01 to 0·02) | p=0·40 | p<0·0001 |
| Month start of data collection | 11 | -0·01 (-0·02 to -0·00) | **p=0·04** | p<0·0001 |
| Time of measurement during pandemic | 11 | -0·06 (-0·11 to 0·00) | p=0·05 | p<0·0001 |
| Publication year | 11 | -0·08 (-0·22 to 0·05) | p=0·24 | p<0·0001 |
| Stringency Index | 11 | 0·01 (-0·00 to 0·01) | p=0·10 | p<0·0001 |
| School Closure Index | 11 | 0·11 (0·02 to 0·20) | **p=0·02** | p<0·0001 |
| Sample size | 11 | -0·00 (-0·00 to 0·00) | p=0·24 | p<0·0001 |

# Table S14. Moderator analysis for female subsample with categorical moderators

| **Categorical moderators** | **k** | **Estimate (95% CI)** | **Test of moderators** | **Test for residual heterogeneity** |
| --- | --- | --- | --- | --- |
| Risk of Bias | 18 | 0·11 (-0·12 to 0·34) | p=0·33 | p<0·0001 |
| Age | 18 | 0·09 (-0·20 to 0·38) | p=0·49 | p<0·0001 |
| Symptom reporter | 18 | 0·66 (-0·10 to 1·44) | p=0·09 | p<0·0001 |
| Country | 18 | -0·00 (-0·04 to 0·04) | p=0·94 | p<0·0001 |
| Stringency Index | 18 | 0·00 (-0·01 to 0·01) | p=0·69 | p<0·0001 |
| School Closure Index | 18 | 0·00 (-0·13 to 0·13) | p=0·99 | p<0·0001 |
| Study design | 18 | 0·14 (-0·12 to 0·41) | p=0·27 | p<0·0001 |

# Table S15. Moderator analysis for female subsample with continuous moderators

| **Continuous moderators** | **k** | **Estimate (95% CI)** | **Test of moderators** | **Test for residual heterogeneity** |
| --- | --- | --- | --- | --- |
| Time of measurement during pandemic | 18 | -0·01 (-0·13 to 0·11) | p=0·88 | p<0·0001 |
| Publication year | 18 | -0·16 (-0·33 to 0·00) | p=0·05 | p<0·0001 |
| Stringency Index | 18 | 0·11 (-0·12 to 0·34) | p=0·34 | p<0·0001 |
| School Closure Index | 18 | 0·11 (-0·12 to 0·34) | p=0·34 | p<0·0001 |
| Sample size | 18 | -0·00 (-0·00 to 0·00) | p=0·96 | p<0·0001 |

# Table S16. Moderator analysis for male subsample with categorical moderators

| **Categorical moderators** | **k** | **Estimate (95% CI)** | **Test of moderators** | **Test for residual heterogeneity** |
| --- | --- | --- | --- | --- |
| Risk of Bias | 18 | 0·15 (-0·14 to 0·44) | p=0·29 | p<0·0001 |
| Age | 18 | 0·20 (-0·16 to 0·55) | p=0·26 | p<0·0001 |
| Symptom reporter | 18 | 0·77 (-0·23 to 1·76) | p=0·12 | p<0·0001 |
| Country | 18 | -0·01 (-0·05 to 0·05) | p=0·84 | p<0·0001 |
| Stringency Index | 18 | 0·18 (-0·11 to 0·47) | p=0·20 | p<0·0001 |
| School Closure Index | 18 | 0·20 (-0·09 to 0·48) | p=0·16 | p<0·0001 |
| Study design | 18 | 0·02 (-0·34 to 0·37) | p=0·93 | p<0·0001 |

# Table S17. Moderator analysis for male subsample with continuous moderators

| **Continuous moderators** | **k** | **Estimate (95% CI)** | **Test of moderators** | **Test for residual heterogeneity** |
| --- | --- | --- | --- | --- |
| Age | 18 | -0·05 (-0·13 to 0·02) | p=0·14 | p<0·0001 |
| Time of measurement during pandemic | 18 | -0·01 (-0·16 to 0·14) | p=0·85 | p<0·0001 |
| Publication year | 18 | -0·07 (-0·31 to 0·16) | p=0·51 | p<0·0001 |
| Stringency Index | 18 | 0·01 (-0·00 to 0·02) | p=0·15 | p<0·0001 |
| School Closure Index | 18 | 0·11 (-0·05 to 0·26) | p=0·17 | p<0·0001 |
| Sample size | 18 | -0·00 (-0·00 to 0·00) | p=0·76 | p<0·0001 |

# Table S18. Sensitivity analysis

| **Population/ Subpopulation** | **Comparison (number of studies per comparison group in parentheses)** | **Effect estimates  for low RoB studies**  **SMD (95% CI); I²** | **Effect estimates  for all studies**  **SMD (95% CI)** |
| --- | --- | --- | --- |
| Total | Cohort studies (13) vs  Cross sectional studies (4) | 0·20 (0·14 to 0·26); 82%  0·24 (-0·01 to 0·49); 98% | 0·15 (0·07 to 0·23); 92%  0·19 (0·01 to 0·37); 96% |
| Total | Adjusted studies (2) vs  unadjusted studies (15) | 0·08 (-0·03 to 0·19), 57%  0·24 (0·16 to 0·31); 95% | 0·08 (-0·03 to 0·19), 57%  0·17 (0·10 to 0·23); 94% |
| Total | Converted studies (2) vs  unconverted studies (15) | 0·14 (0·03 to 0·25), **only 1 study**  0·22 (0·15 to 0·29); 95% | -0·15 (-0·26 to -0·04), **only 1 study**  0·18 (0·11 to 0·24); 94% |

# Table S19. Eggers’ test

| **Population** | **p** |
| --- | --- |
| Total population | 0·93 |
| Females | 0·82 |
| Males | 0·42 |
| Total population, 11-15y | 0·55 |
| Females, 11-15y | 0·37 |
| Males, 11-15y | 0·36 |
| Total population, 16-19y | 0·69 |
| Females, 16-19y | 0·91 |
| Males, 16-19y | 0·14 |

# Figure S1: PRISMA Flow Chart


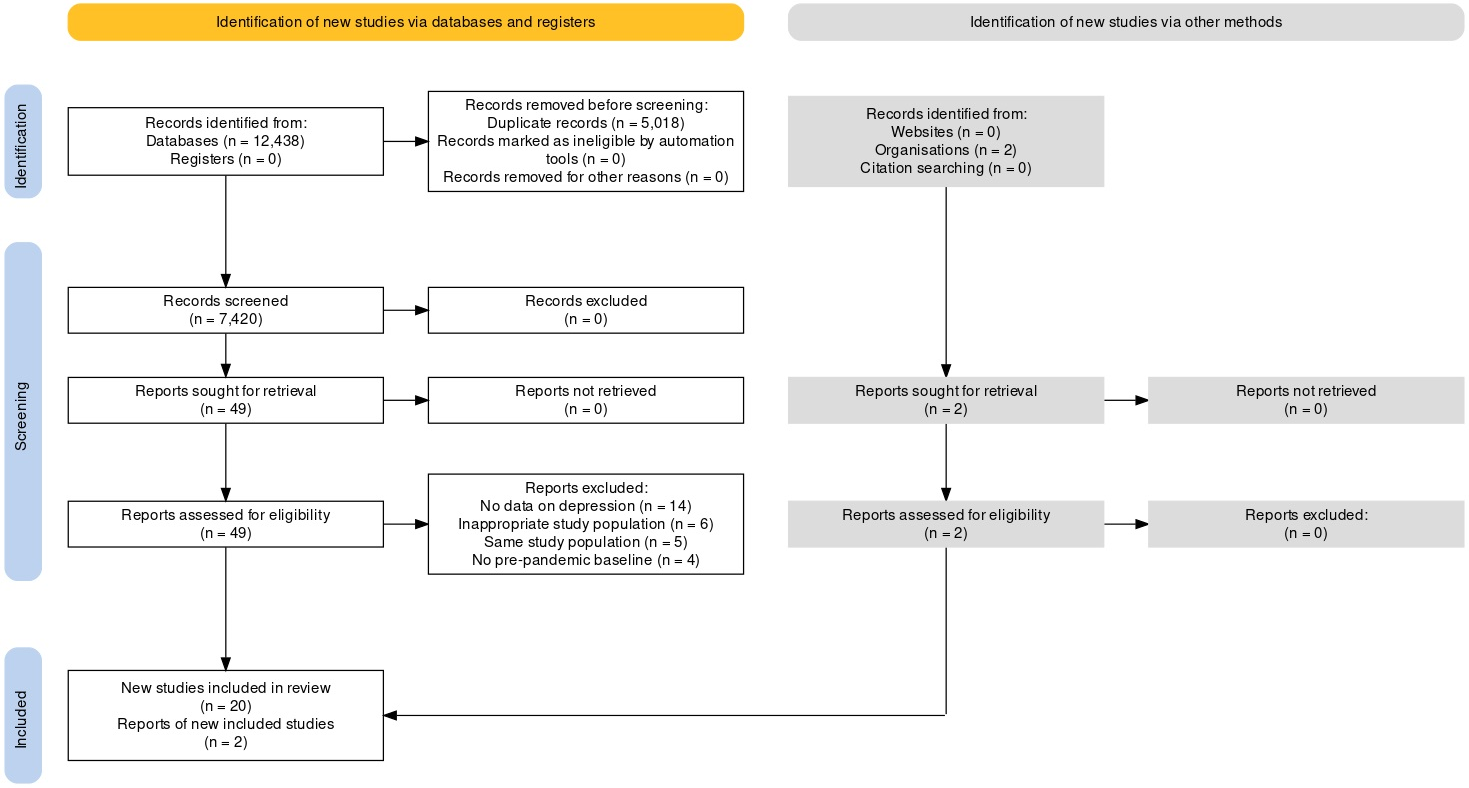


# Figure S2: Traffic light plots of the domain-level judgements for each individual result


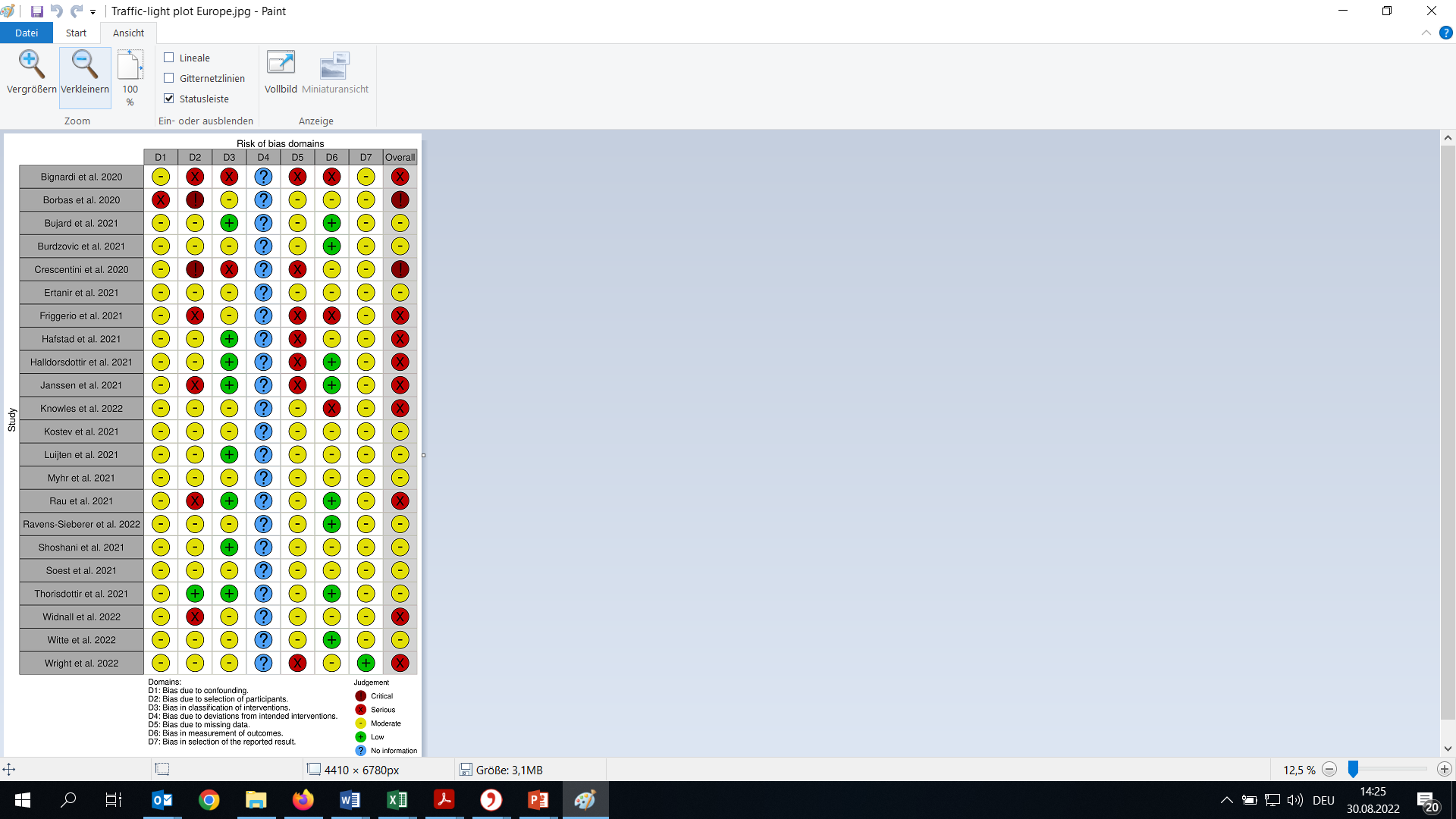


**Domains:** **Judgement**


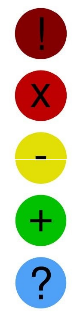
D1: Bias due to confounding

Critical

Serious

Moderate

Low

Not applicable

D2: Bias in selection of participants into the study

D3: Bias in classification of exposures

D4: Bias due to departures from intended exposures

D5: Bias due to missing data

D6: Bias in measurements of outcomes

D7: Bias in selection of reported results

Figure was created using the tool *robvis* [44].

# Figure S3: Weighted bar plots of the distribution of risk of bias judgements within each bias domain


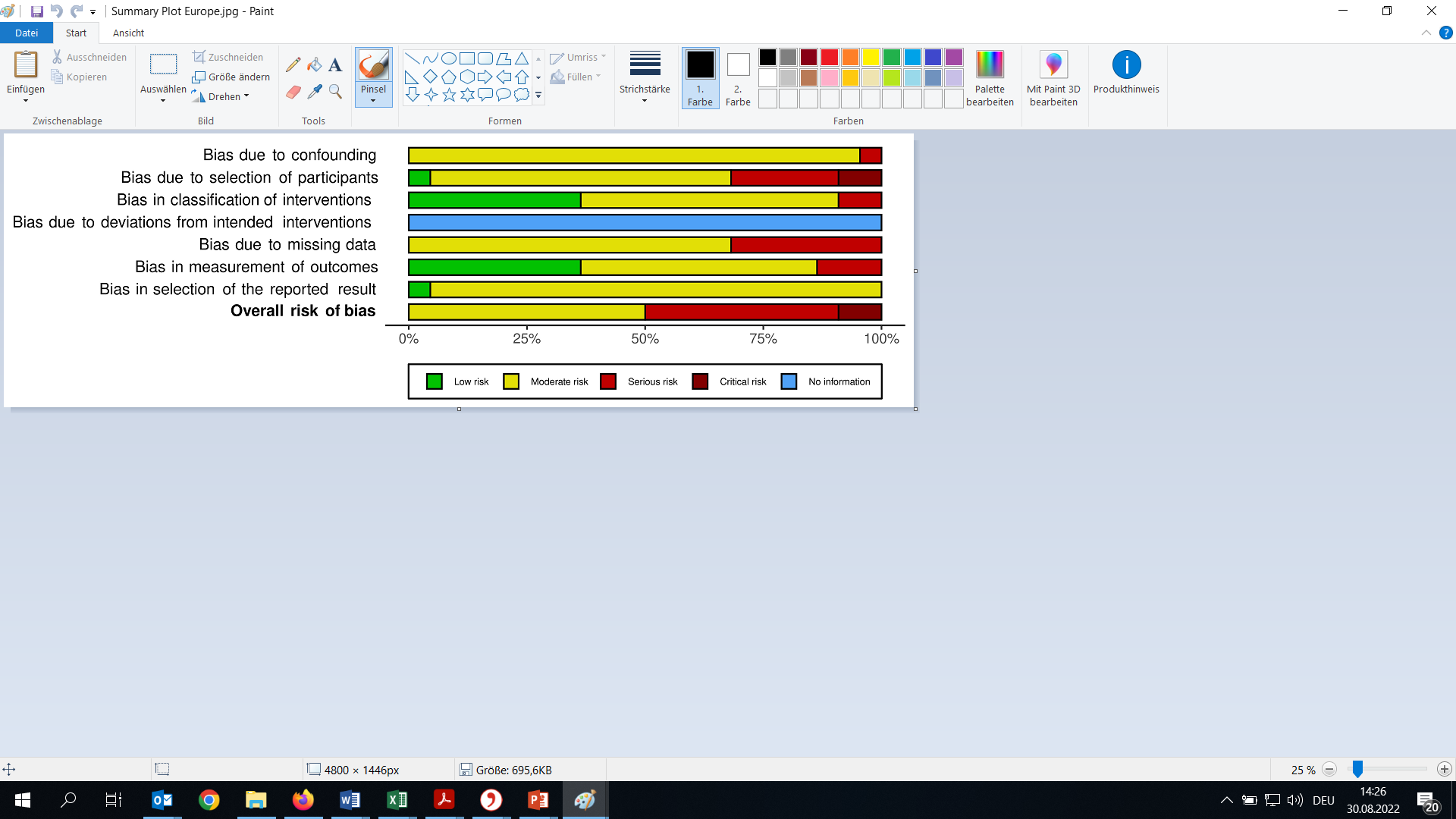


Figure was created using the tool *robvis* [44].

# Figure S4: Forest Plot of Changes in Female General Depression Symptoms Comparing Before and During COVID-19 Pandemic


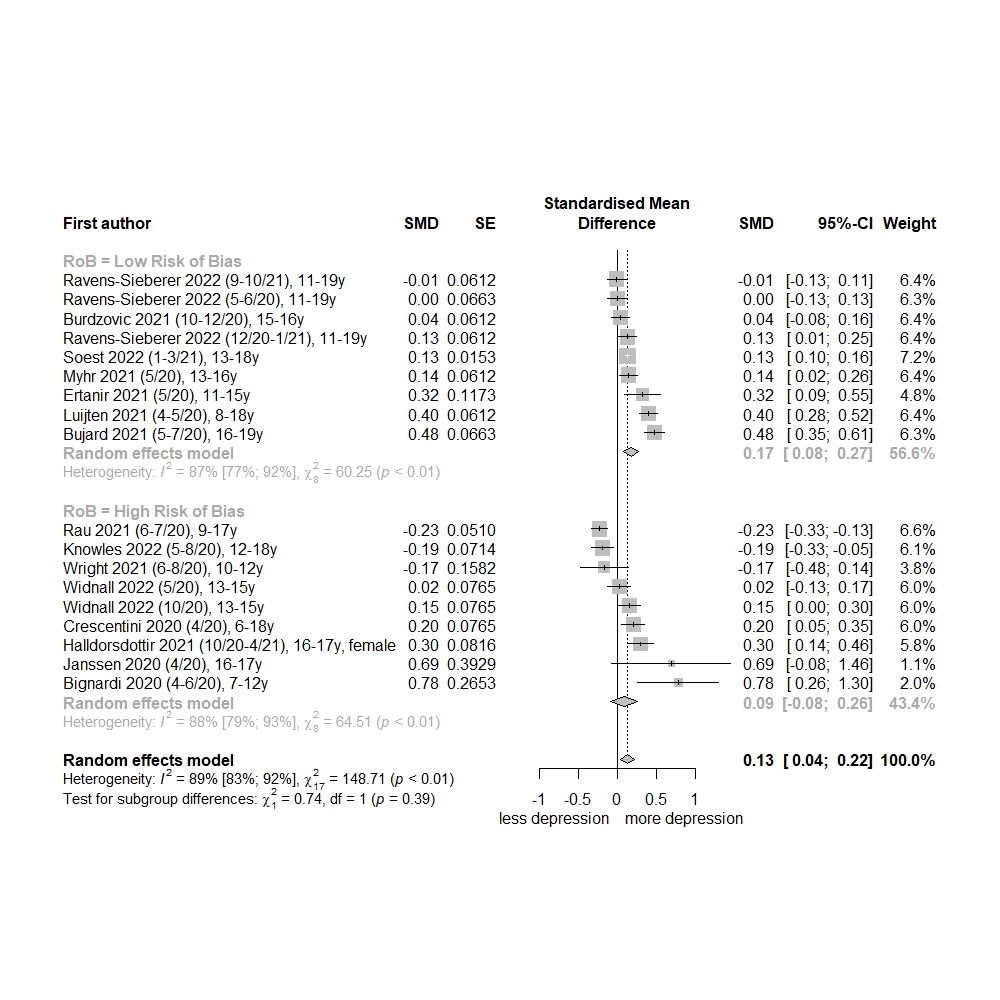


# Figure S5: Forest Plot of Changes in Male General Depression Symptoms Comparing Before and During COVID-19 Pandemic


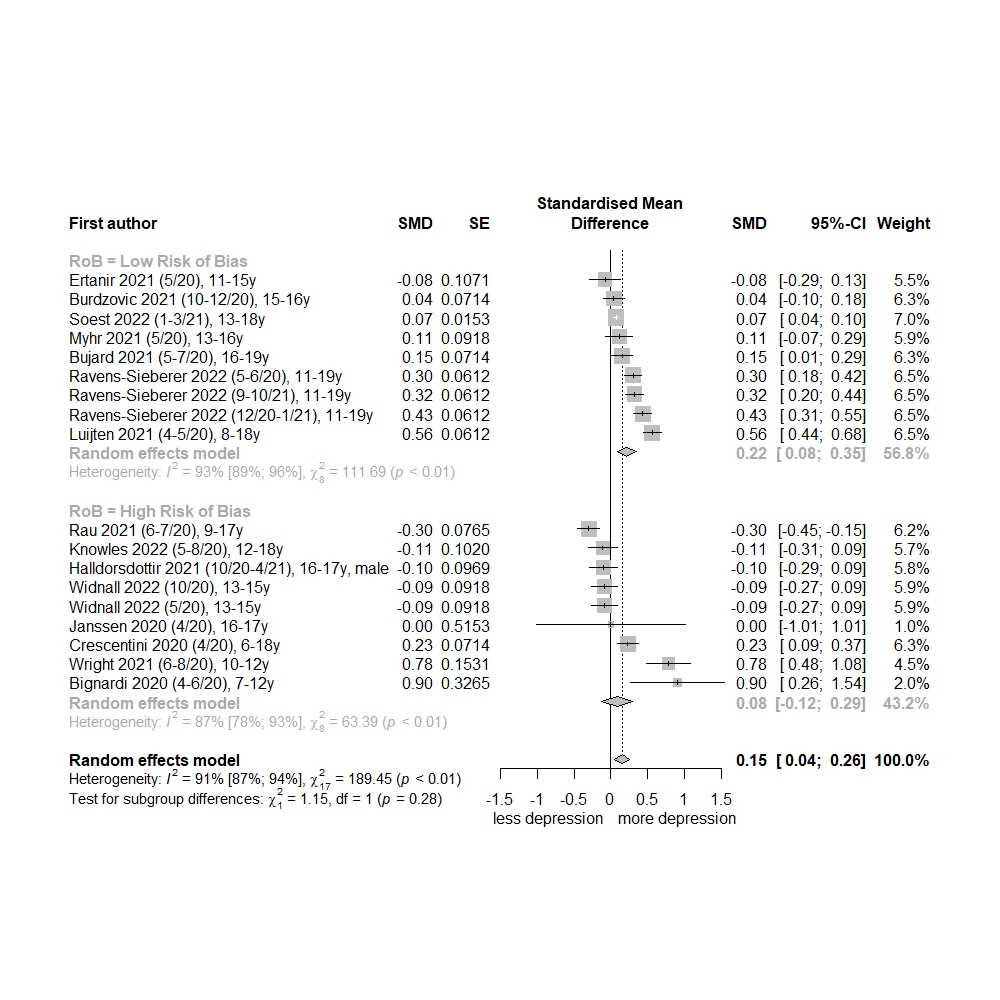


# Figure S6. Forest Plot of Changes in Total (11-15 years) General Depression Symptoms Comparing Before and During COVID-19 Pandemic


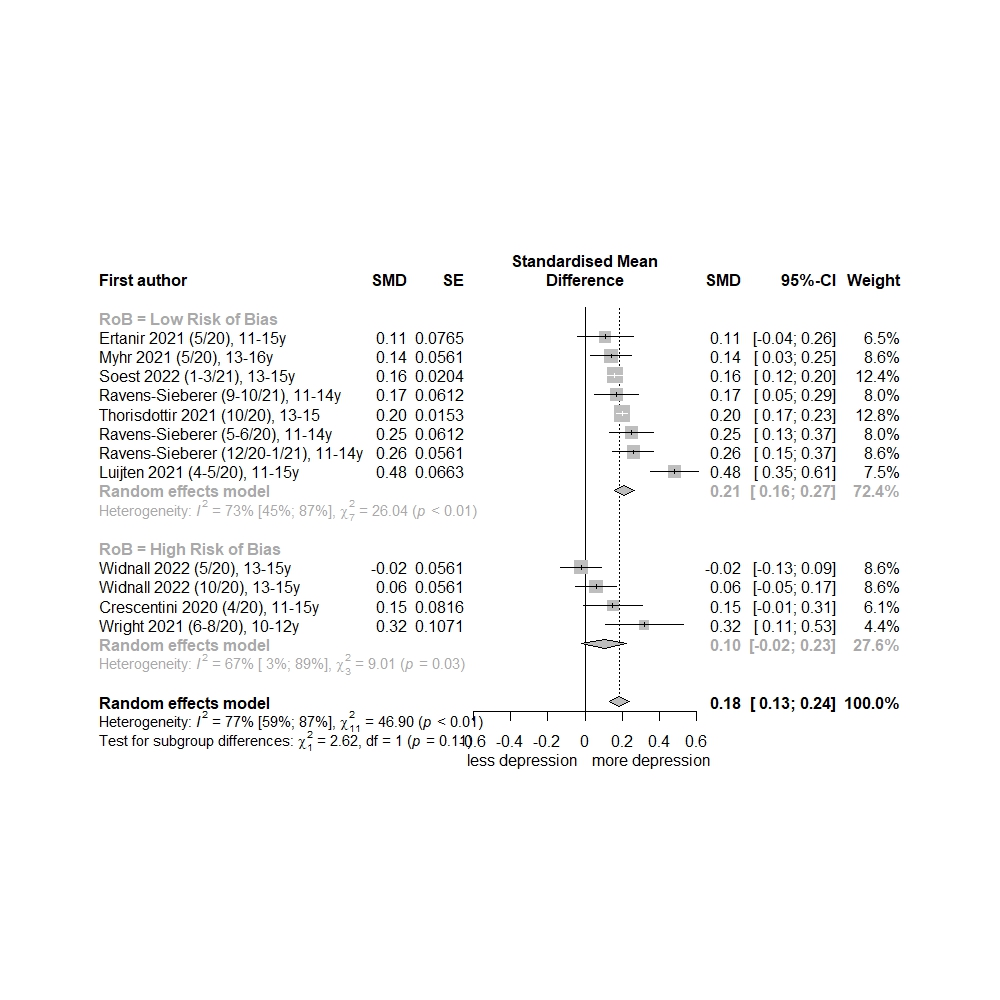


# Figure S7. Forest Plot of Changes in Female (11-15 years) General Depression Symptoms Comparing Before and During COVID-19 Pandemic


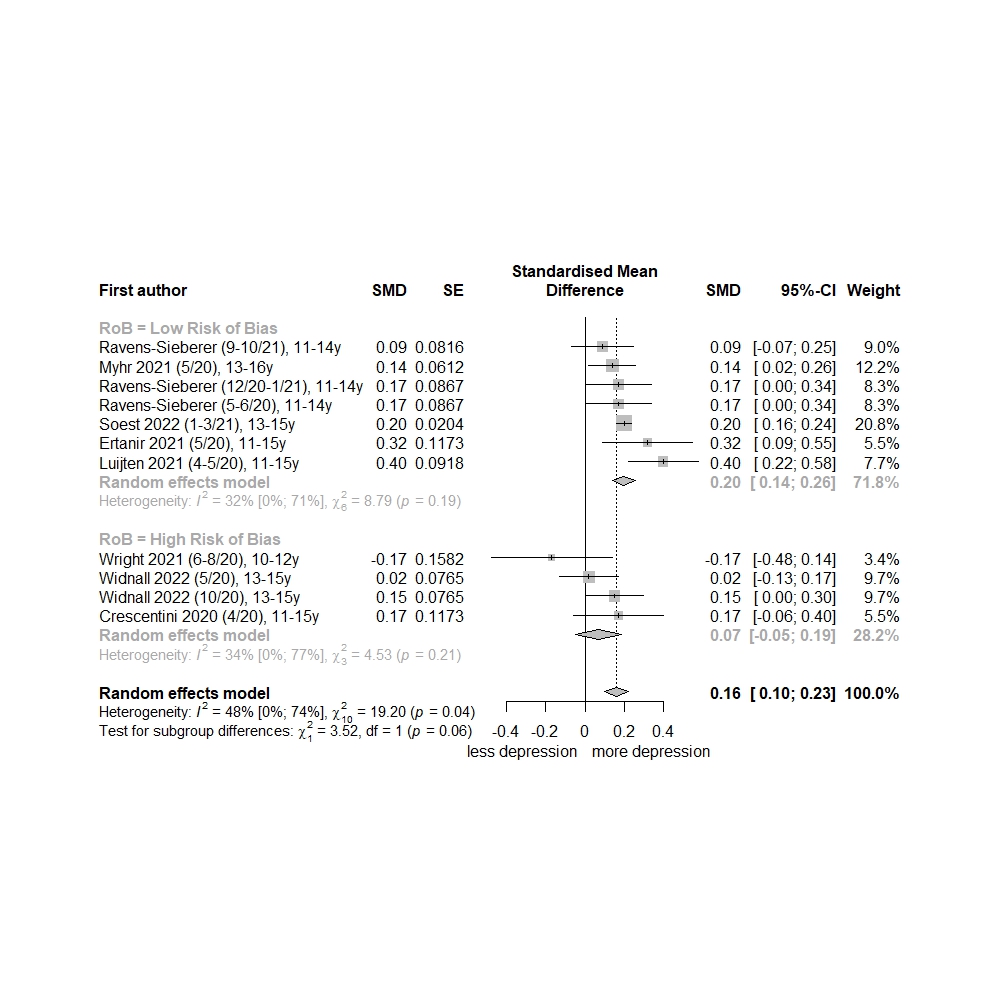


# Figure S8. Forest Plot of Changes in Male (11-15 years) General Depression Symptoms Comparing Before and During COVID-19 Pandemic


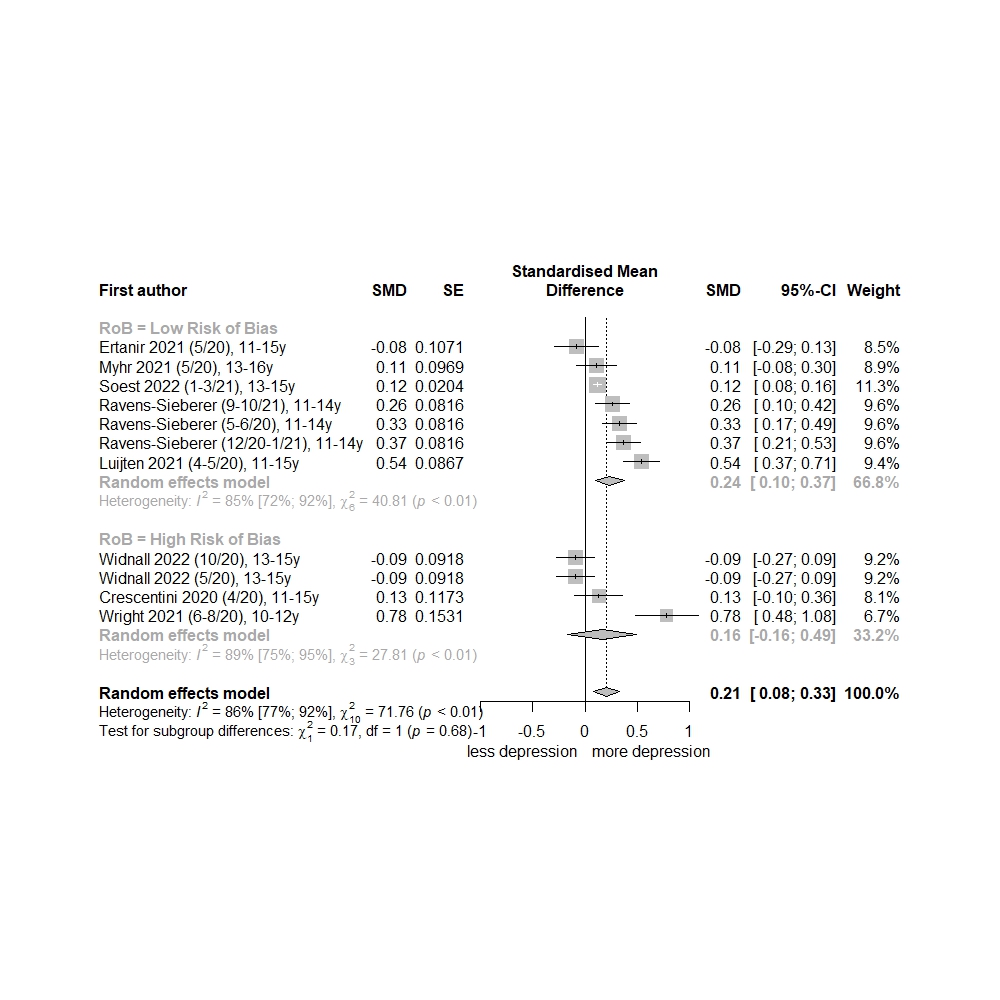


# Figure S9. Forest Plot of Changes in Total (16-19 years) General Depression Symptoms Comparing Before and During COVID-19 Pandemic


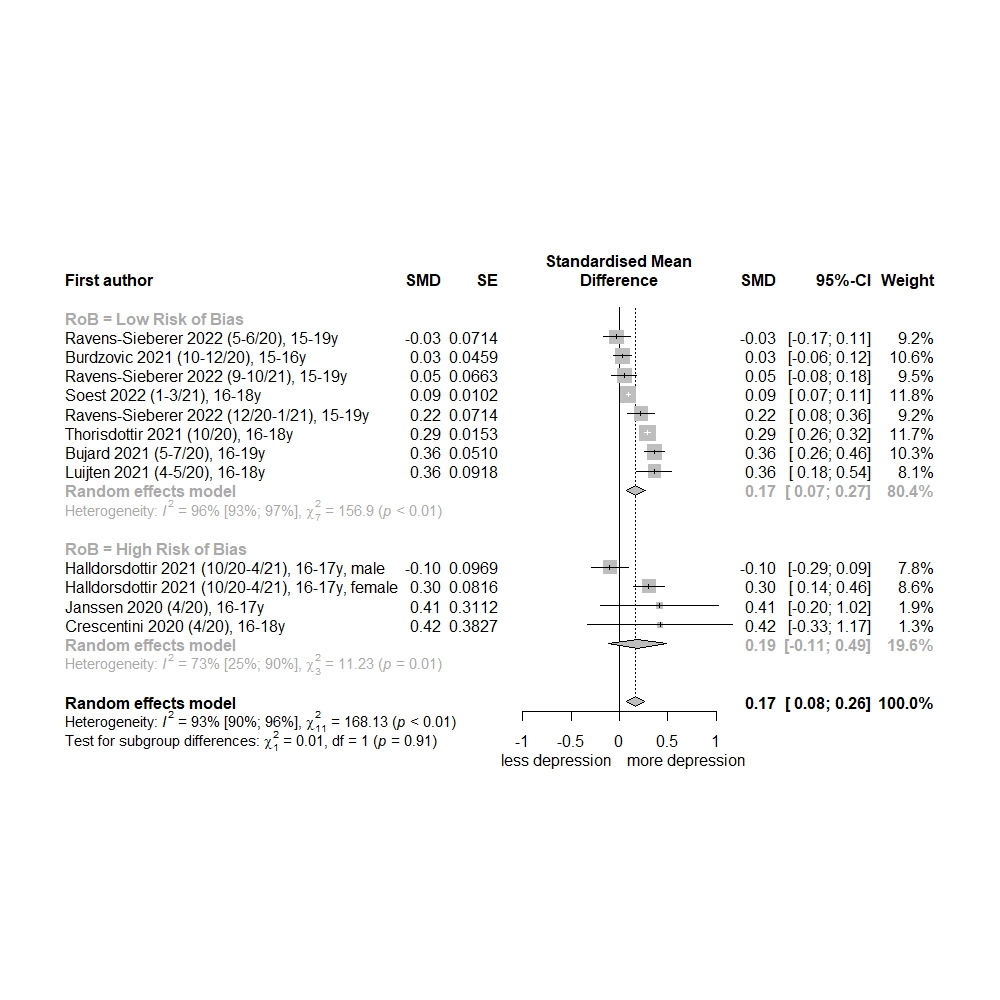


# Figure S10. Forest Plot of Changes in Male (16-19 years) General Depression Symptoms Comparing Before and During COVID-19 Pandemic


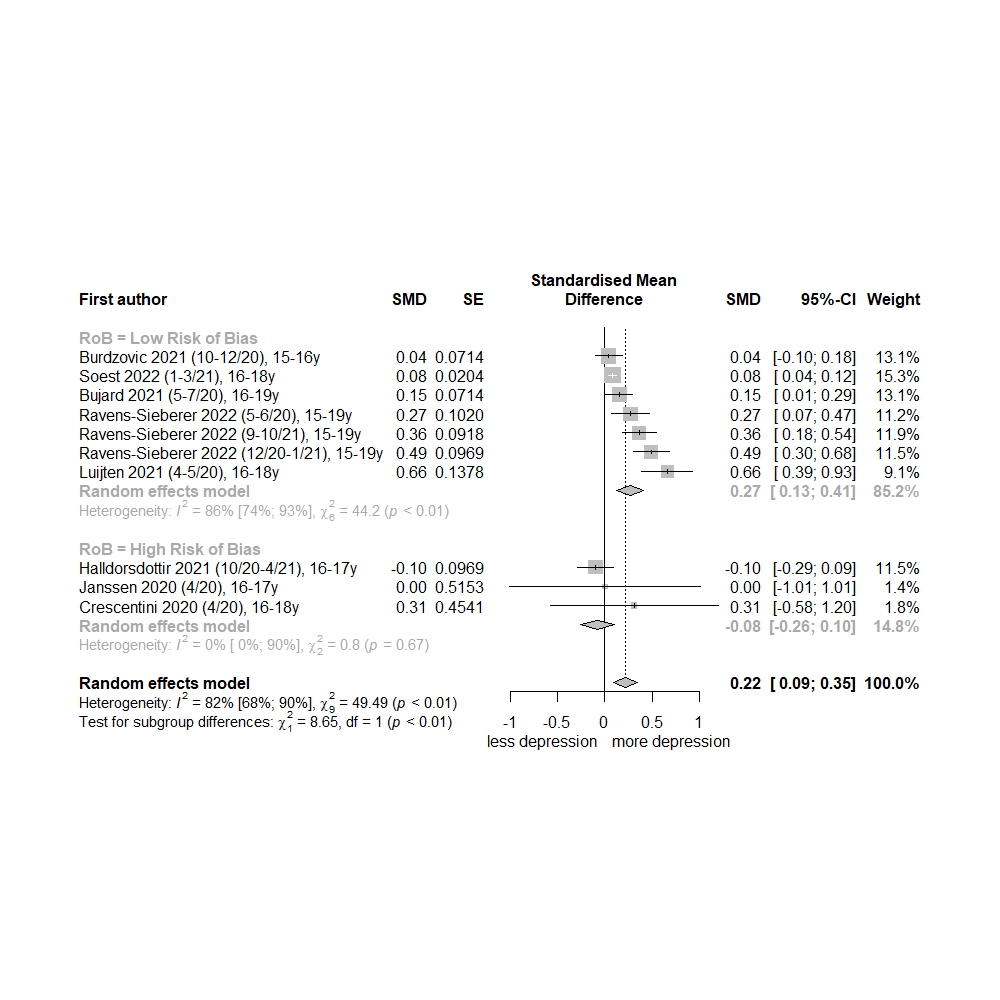


# Figure S11. Forest Plot of Changes in Female (16-19 years) General Depression Symptoms Comparing Before and During COVID-19 Pandemic


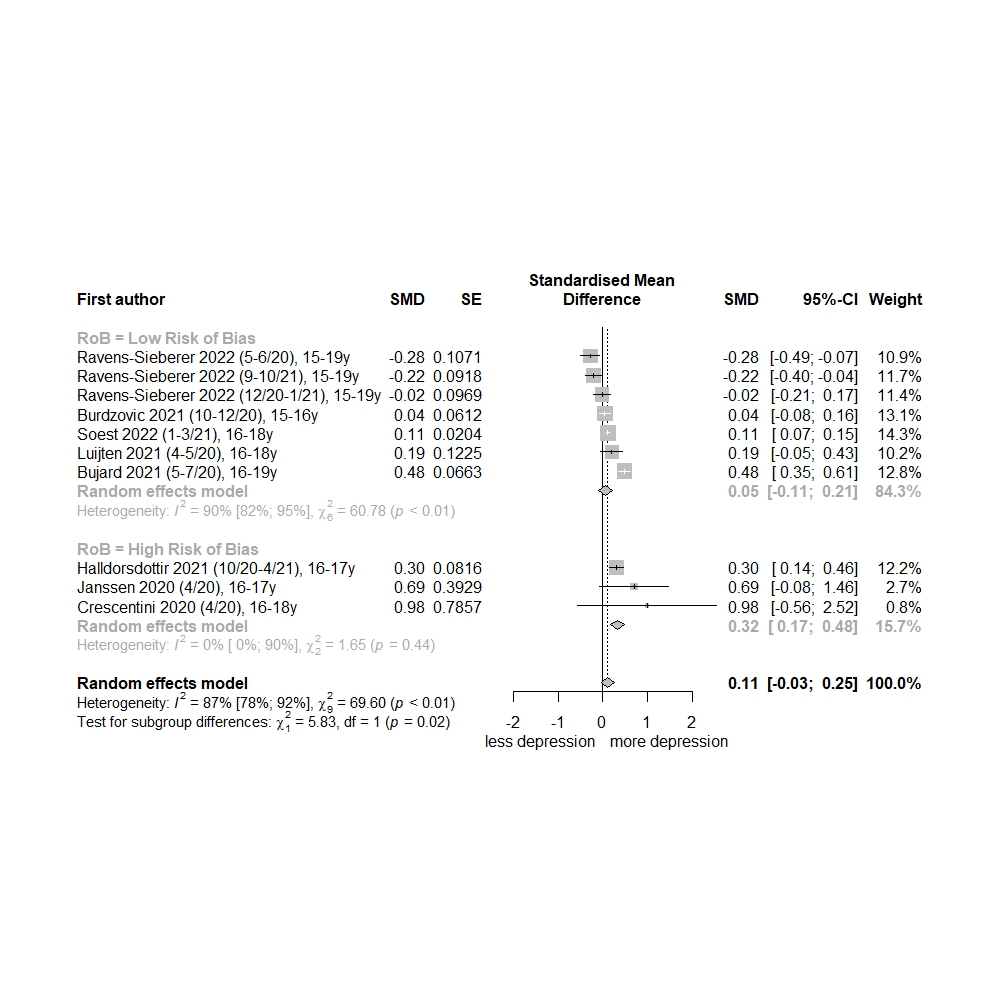


# Figure S12. Plot of change effects (standardized mean differences and 95% confidence interval) from low RoB studies on a time axis


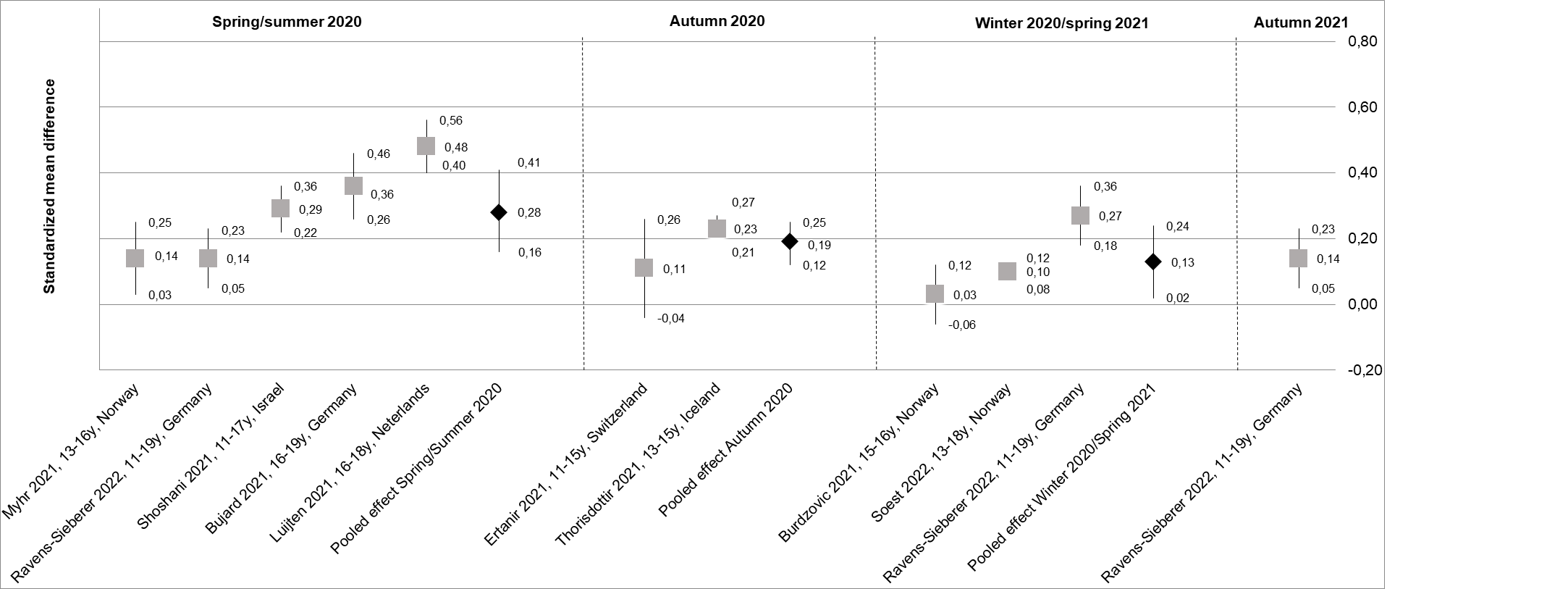


# Figure S13. Forest Plot of Country Changes in General Depression Symptoms Comparing Before and During COVID-19 Pandemic


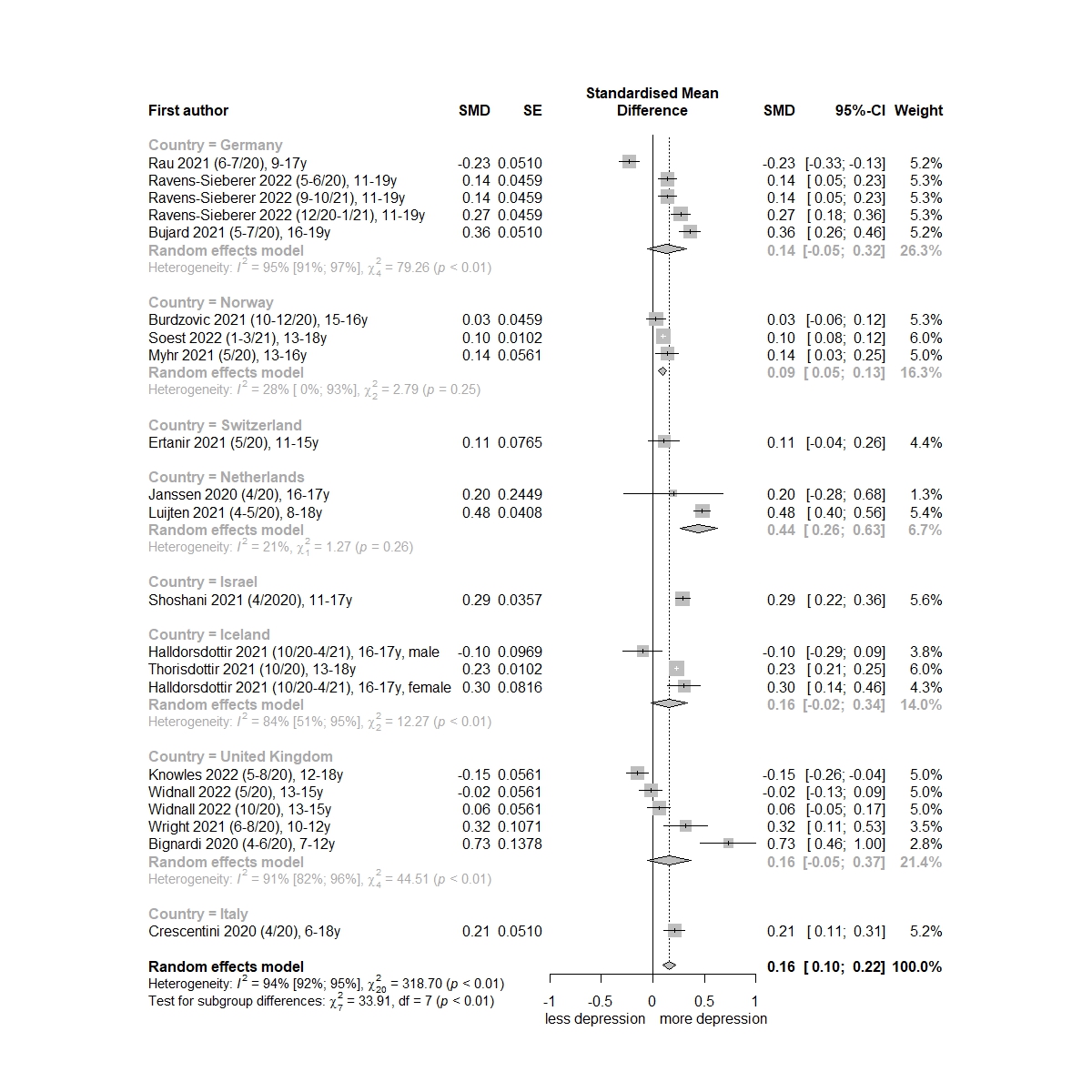


# Figure S14. Forest Plot of Changes in Female Clinically Relevant Depression Rates Comparing Before and During COVID-19 Pandemic


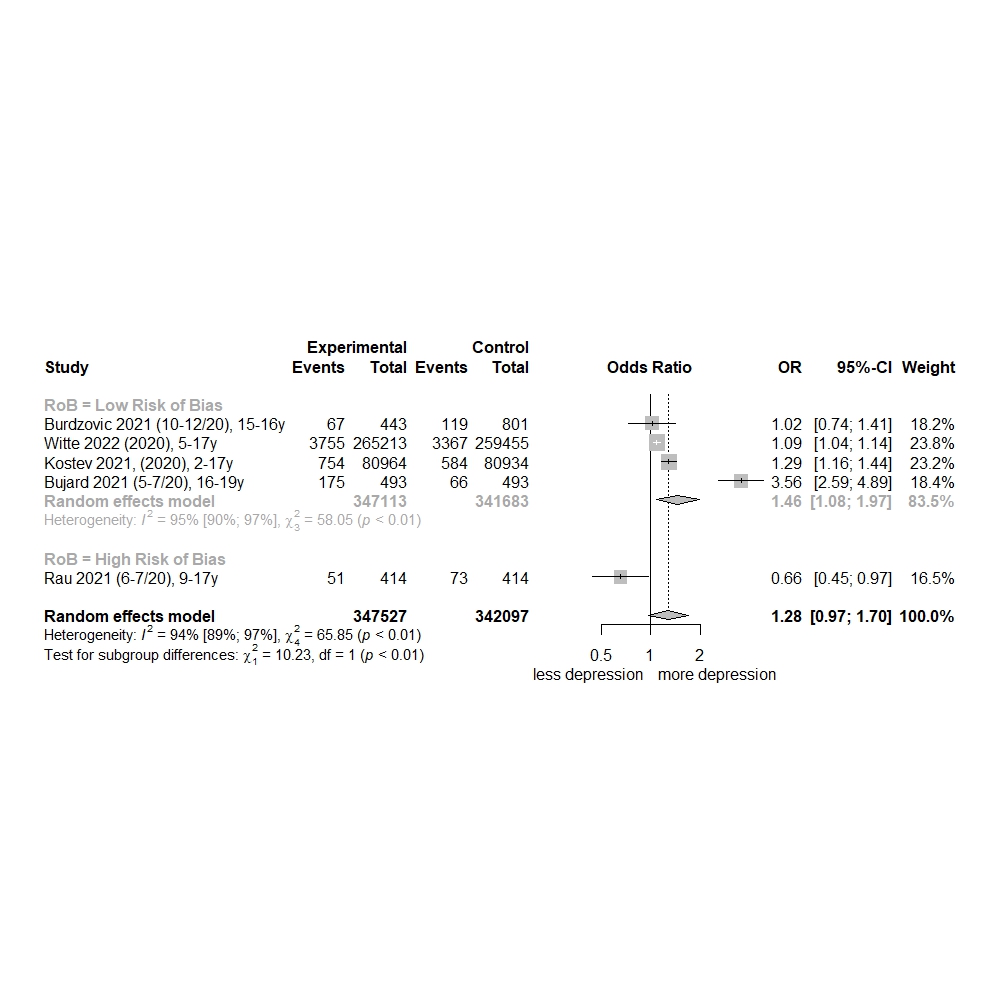


# Figure S15. Forest Plot of Changes in Male Clinically Relevant Depression Rates Comparing Before and During COVID-19 Pandemic


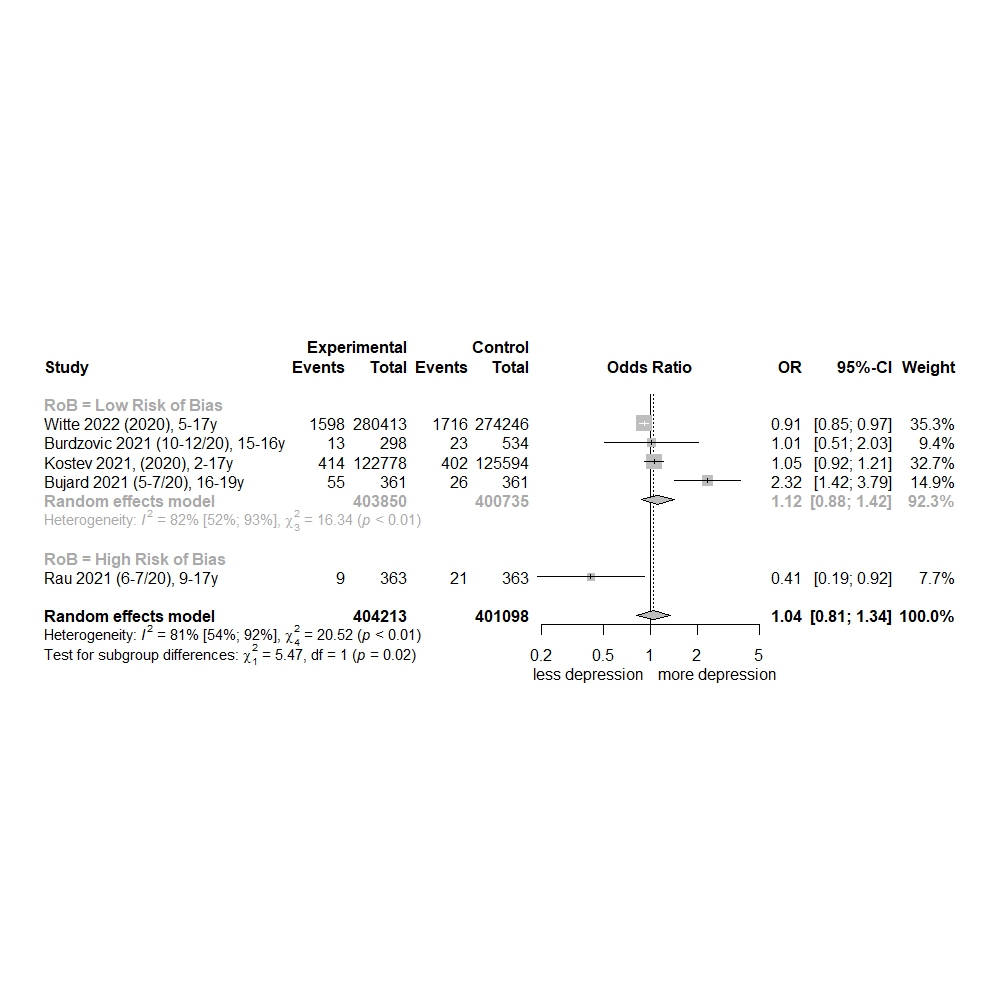


# Figure S16. Funnel Plot of Changes in Total General Depression Symptoms Comparing Before and During COVID-19 Pandemic


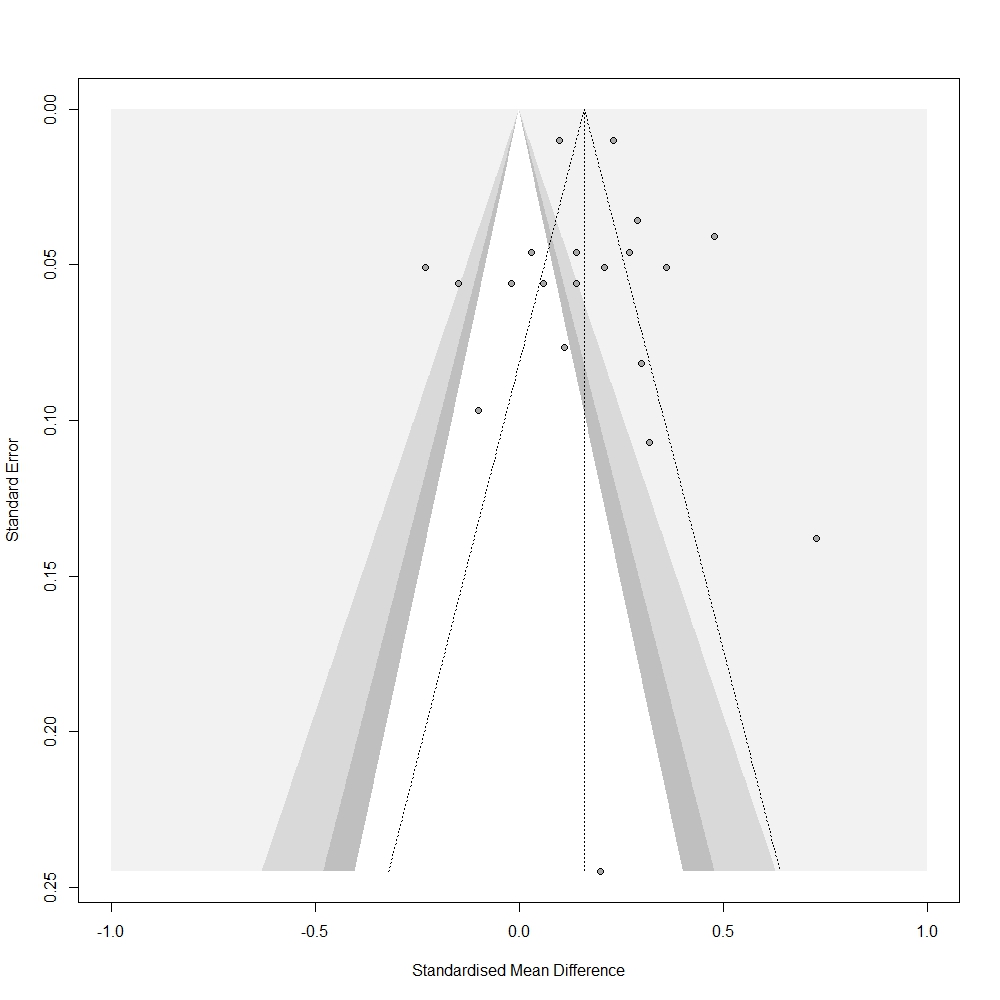


# Figure S17. Funnel Plot of Changes in Female General Depression Symptoms Comparing Before and During COVID-19 Pandemic


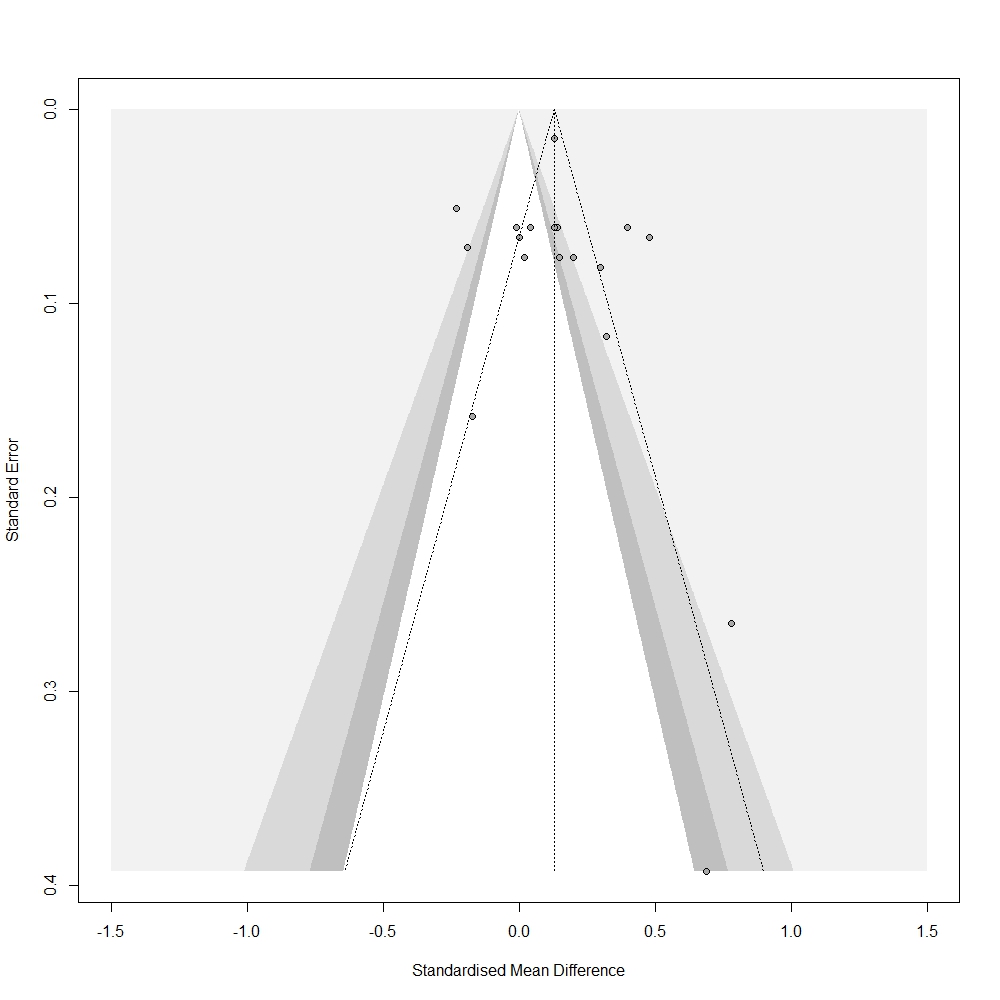


# Figure S18. Funnel Plot of Changes in Male General Depression Symptoms Comparing Before and During COVID-19 Pandemic


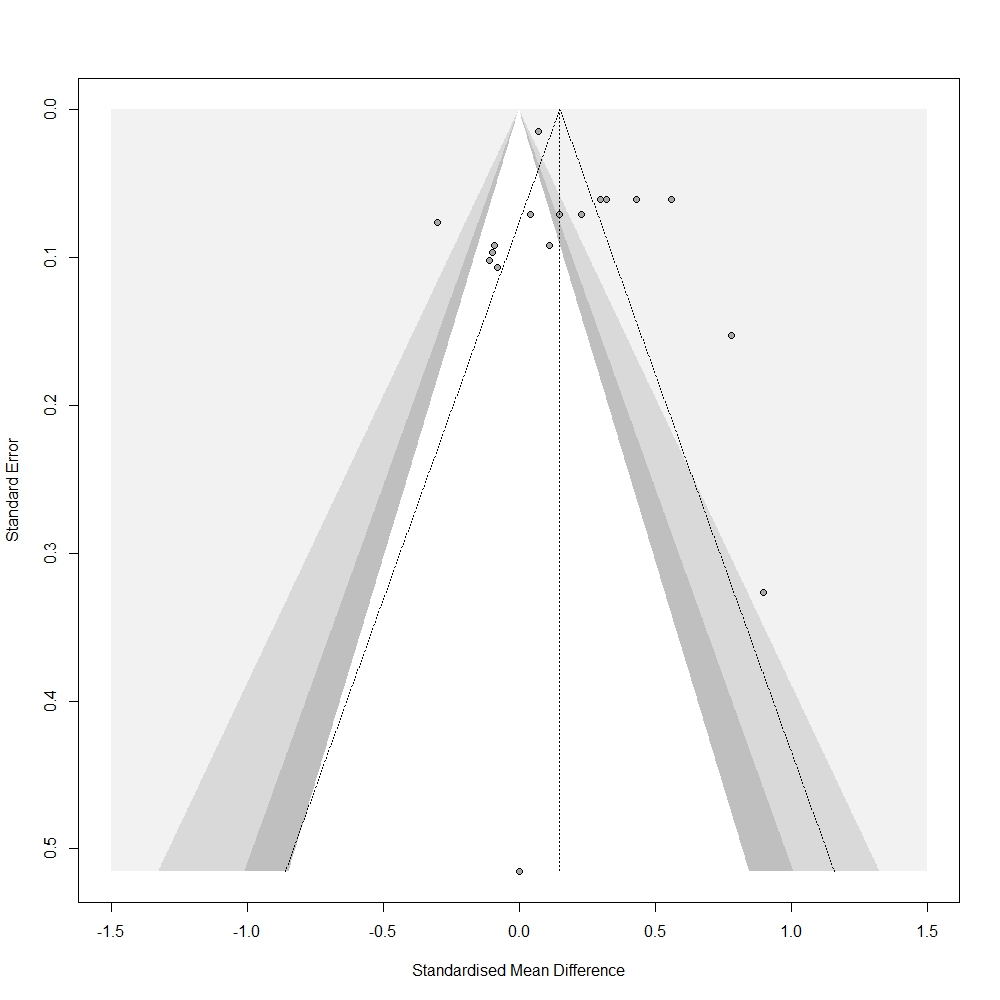


# Figure S19. Funnel Plot of Changes in Total Clinically Relevant Depression Symptoms Comparing Before and During COVID-19 Pandemic


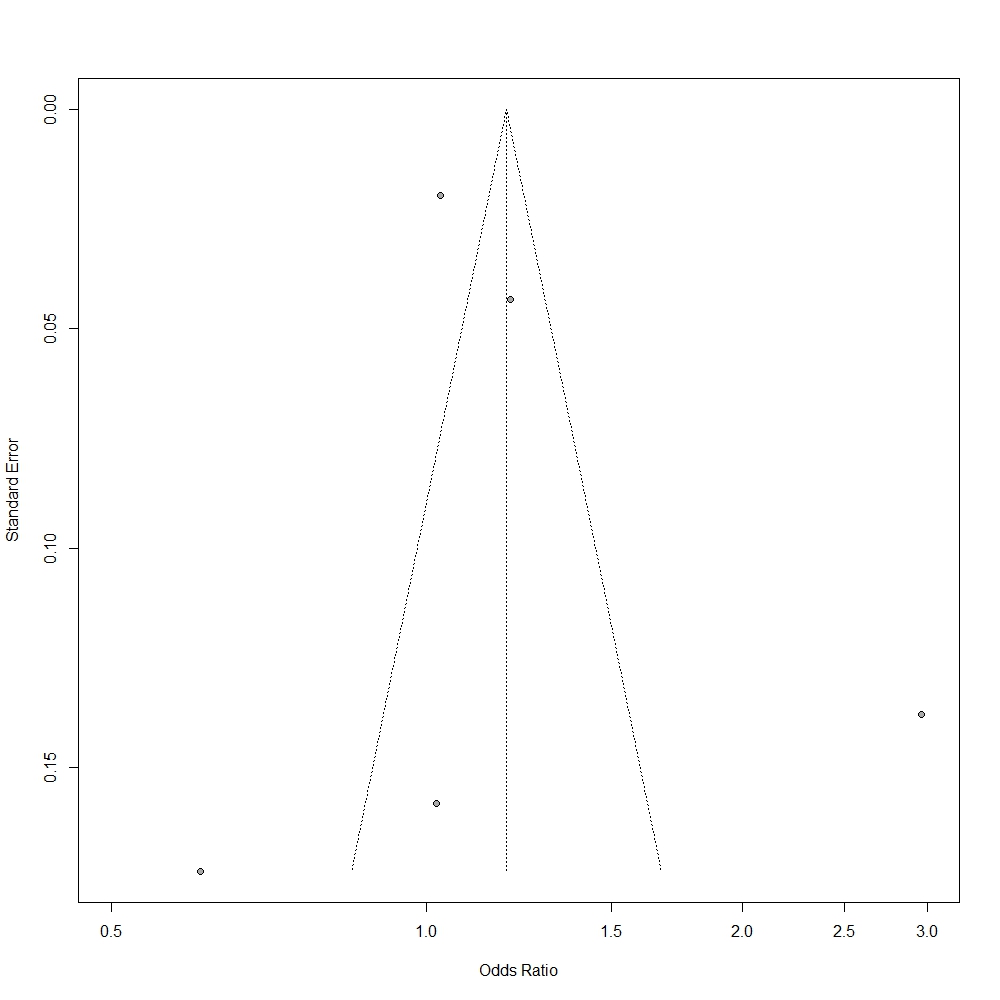


# Figure S20. Funnel Plot of Changes in Female Clinically Relevant Depression Symptoms Comparing Before and During COVID-19 Pandemic


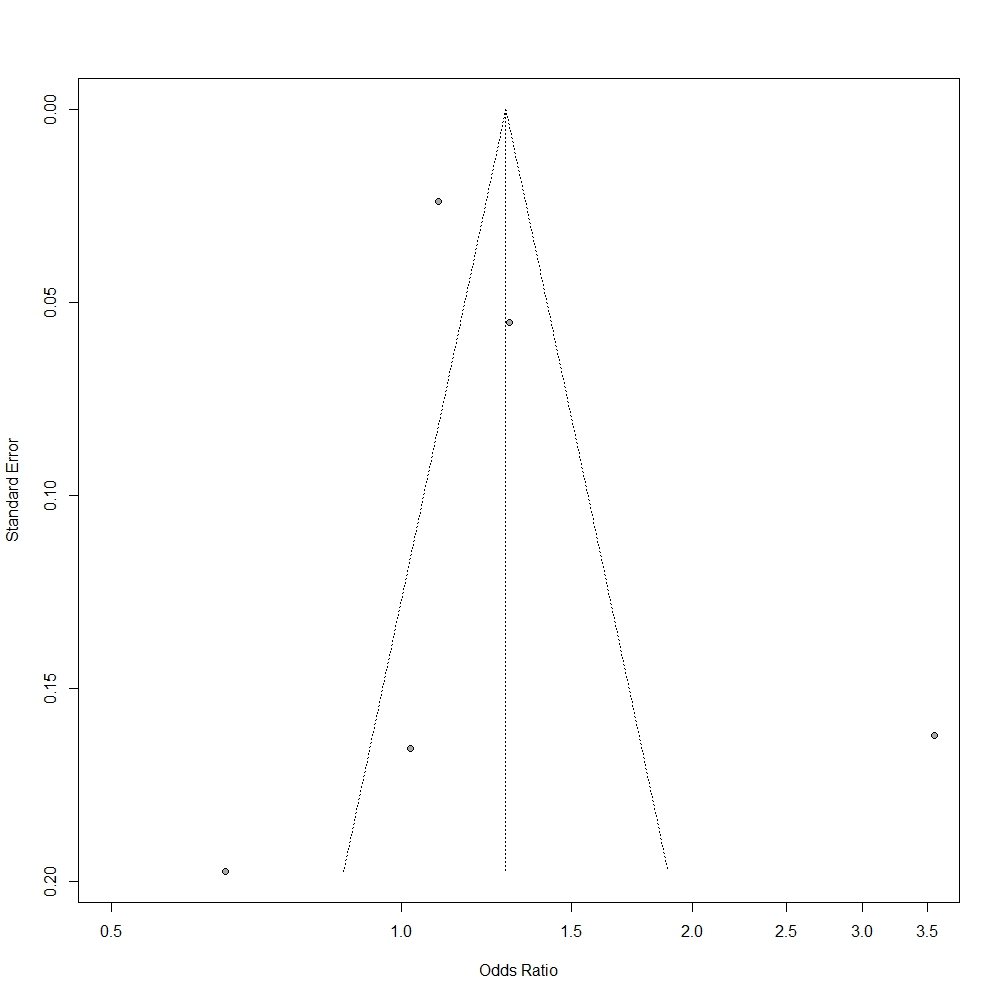


# Figure S21. Funnel Plot of Changes in Male Clinically Relevant Depression Symptoms Comparing Before and During COVID-19 Pandemic


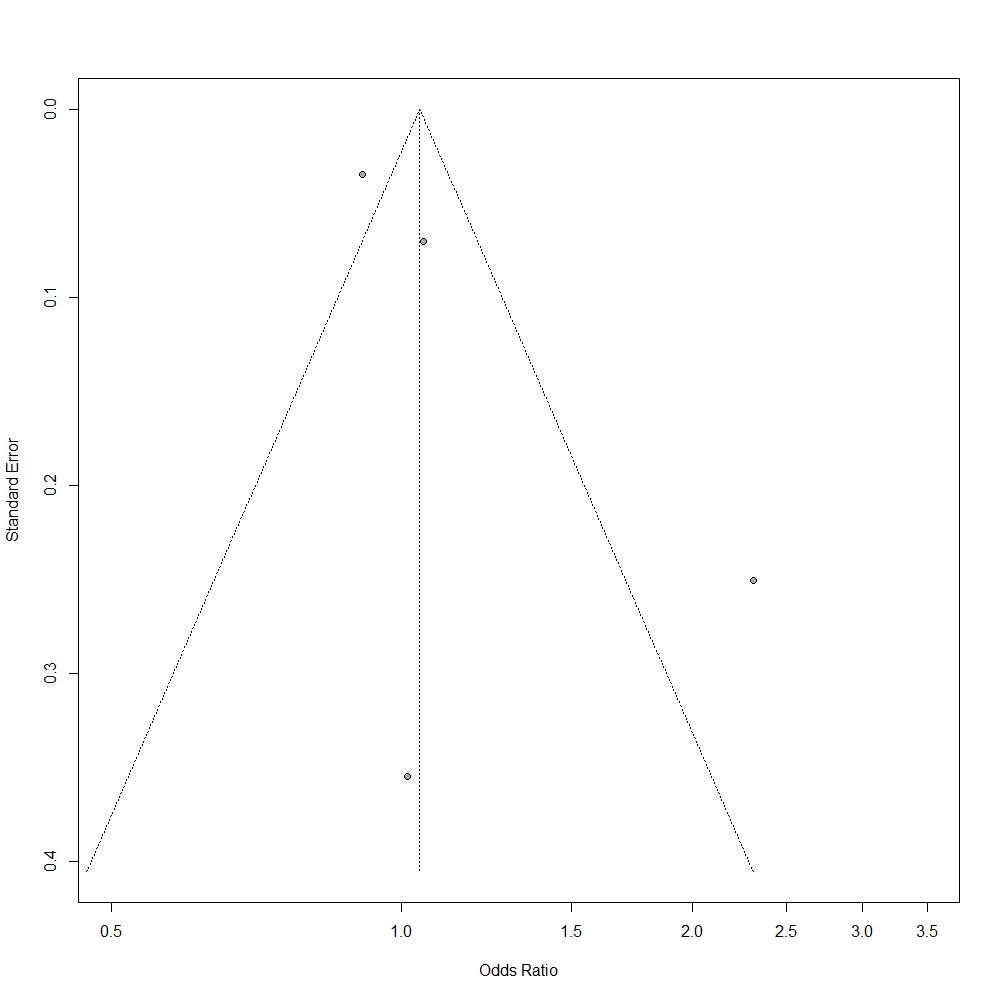


# Methods: Oxford COVID-19 Stringency Index and the School Closure Index

| **Index** | **Description** |
| --- | --- |
| Oxford COVID-19 Stringency Index [45] | The Oxford COVID-19 Stringency Index is a composition of 9 indicators including school closures, workplace closures and stay-at-home requirements. Total scores estimate range from 0 (no restrictions) to 100 (most stringent restrictions). A validation of the score in form of demonstrating associations with population SARS-CoV-2 infection rates and mobile phone mobility data has occurred [45]. A mean score was calculated for each study for the measurement period during the COVID-19 pandemic.  The cut-offs were specify in accordance with COVIDSurg Collaborative[46], defining 3 stringency groups: light restrictions (index<20), moderate lockdowns (index 20–60) and full lockdowns (index>60). |
| School Closure Index [45, 47] | The School Closure Index records closings of schools and universities within the Oxford COVID-19 Stringency Index. The index ranges from 0 to 3. Whereby 0 stands for no measures, 1 includes recommend closing or all schools open with alterations resulting in significant differences compared to non-COVID-19 operations, 2 require closing (only some levels or categories, e.g. just high school, or just public schools) and 3 require closing all levels [45, 47].  For the subgroup analyses the following cut-offs were defined: no or few alterations compared to a pre-COVID-19 situation (index<2) and partial or full school closure (index≥2). |

References

1. Morgan RL, Thayer KA, Santesso N, Holloway AC, Blain R, Eftim SE, et al. A risk of bias instrument for non-randomized studies of exposures: A users' guide to its application in the context of GRADE. Environ Int. 2019;122:168–84. doi:10.1016/j.envint.2018.11.004.

2. Schünemann H, Brożek J, Guyatt G, Oxman A. GRADE Handbook: Handbook for grading the quality of evidence and the strength of recommendations using the GRADE approach. Updated October 2013. https://gdt.gradepro.org/app/handbook/handbook.html#h.w6r7mtvq3mjz. Accessed 31 Aug 2022.

3. Schünemann HJ, Cuello C, Akl EA, Mustafa RA, Meerpohl JJ, Thayer K, et al. GRADE guidelines: 18. How ROBINS-I and other tools to assess risk of bias in nonrandomized studies should be used to rate the certainty of a body of evidence. J Clin Epidemiol. 2019;111:105–14. doi:10.1016/j.jclinepi.2018.01.012.

4. Bujard M, den Driesch E von, Kerstin R, Laß I, Thönnissen C, Schumann A, Schneider N. Belastungen von Kindern, Jugendlichen und Eltern in der Corona-Pandemie: BiB.Bevölkerungs.Studien 2/2021. Wiesbaden: Bundesinstitut für Bevölkerungsforschung; 2021.

5. Ravens-Sieberer U, Kaman A, Otto C, Adedeji A, Napp A-K, Becker M, et al. Mental health and psychological burden of children and adolescents during the first wave of the COVID-19 pandemic-results of the COPSY study. [Mental health and psychological burden of children and adolescents during the first wave of the COVID-19 pandemic-results of the COPSY study]. Bundesgesundheitsblatt Gesundheitsforschung Gesundheitsschutz. 2021;64:1512–21. doi:10.1007/s00103-021-03291-3.

6. Ravens-Sieberer U, Erhart M, Devine J, Gilbert M, Reiss F, Barkmann C, et al. Child and Adolescent Mental Health During the COVID-19 Pandemic: Results of the Three-Wave Longitudinal COPSY Study. SSRN Journal 2022. doi:10.2139/ssrn.4024489.

7. Ravens-Sieberer U, Kaman A, Erhart M, Devine J, Schlack R, Otto C. Impact of the COVID-19 pandemic on quality of life and mental health in children and adolescents in Germany. Eur Child Adolesc Psychiatry. 2022;31:879–89. doi:10.1007/s00787-021-01726-5.

8. Ravens-Sieberer U, Kaman A, Erhart M, Otto C, Devine J, Löffler C, et al. Quality of life and mental health in children and adolescents during the first year of the COVID-19 pandemic: results of a two-wave nationwide population-based study. Eur Child Adolesc Psychiatry 2021. doi:10.1007/s00787-021-01889-1.

9. Barkmann C, Erhart M, Schulte-Markwort M. The German version of the Centre for Epidemiological Studies Depression Scale for Children: psychometric evaluation in a population-based survey of 7 to 17 years old children and adolescents--results of the BELLA study. Eur Child Adolesc Psychiatry. 2008;17 Suppl 1:116–24. doi:10.1007/s00787-008-1013-0.

10. Witte J, Zeitler A, Hasemann L. Krankenhausversorgung von Kindern und Jugendlichen während der Pandemie: Fokus: Psychische Erkrankungen. Bielefeld; 2022.

11. Spaderna H, Schmukle SC, Krohne HW. Bericht über die deutsche Adaptation der State-Trait Depression Scales (STDS). Diagnostica. 2002;48:80–9. doi:10.1026//0012-1924.48.2.80.

12. Kostev K, Weber K, Riedel-Heller S, Vultée C von, Bohlken J. Increase in depression and anxiety disorder diagnoses during the COVID-19 pandemic in children and adolescents followed in pediatric practices in Germany. Eur Child Adolesc Psychiatry 2021. doi:10.1007/s00787-021-01924-1.

13. Rau L-M, Grothus S, Sommer A, Grochowska K, Claus BB, Zernikow B, Wager J. Chronic Pain in Schoolchildren and its Association With Psychological Wellbeing Before and During the COVID-19 Pandemic. J Adolesc Health. 2021;69:721–8. doi:10.1016/j.jadohealth.2021.07.027.

14. Chorpita BF, Yim L, Moffitt C, Umemoto LA, Francis SE. Assessment of symptoms of DSM-IV anxiety and depression in children: a revised child anxiety and depression scale. Behav Res Ther. 2000;38:835–55. doi:10.1016/s0005-7967(99)00130-8.

15. Thorisdottir IE, Asgeirsdottir BB, Kristjansson AL, Valdimarsdottir HB, Jonsdottir Tolgyes EM, Sigfusson J, et al. Depressive symptoms, mental wellbeing, and substance use among adolescents before and during the COVID-19 pandemic in Iceland: a longitudinal, population-based study. The Lancet Psychiatry. 2021;8:663–72. doi:10.1016/S2215-0366(21)00156-5.

16. Rytilä-Manninen M, Fröjd S, Haravuori H, Lindberg N, Marttunen M, Kettunen K, Therman S. Psychometric properties of the Symptom Checklist-90 in adolescent psychiatric inpatients and age- and gender-matched community youth. Child Adolesc Psychiatry Ment Health. 2016;10:23. doi:10.1186/s13034-016-0111-x.

17. Halldorsdottir T, Thorisdottir IE, Meyers CCA, Asgeirsdottir BB, Kristjansson AL, Valdimarsdottir HB, et al. Adolescent well-being amid the COVID-19 pandemic: Are girls struggling more than boys? JCPP Adv. 2021;1:e12027. doi:10.1002/jcv2.12027.

18. Shoshani A, Kor A. The mental health effects of the COVID-19 pandemic on children and adolescents: Risk and protective factors. Psychol Trauma 2021. doi:10.1037/tra0001188.

19. Frigerio A, Nettuno F, Nazzari S. Maternal mood moderates the trajectory of emotional and behavioural problems from pre- to during the COVID-19 lockdown in preschool children. Eur Child Adolesc Psychiatry 2022. doi:10.1007/s00787-021-01925-0.

20. Achenbach TM, Rescorla L. Manual for the ASEBA Preschool forms & profiles. Burlington, VT: ASEBA; 2001.

21. Crescentini C, Feruglio S, Matiz A, Paschetto A, Vidal E, Cogo P, Fabbro F. Stuck Outside and Inside: An Exploratory Study on the Effects of the COVID-19 Outbreak on Italian Parents and Children's Internalizing Symptoms. Front Psychol. 2020;11:586074. doi:10.3389/fpsyg.2020.586074.

22. Achenbach TM, Rescorla L. Manual for the ASEBA school-age forms & profiles: An integrated system of multi-informant assessment. Burlington, VT: ASEBA; 2001.

23. Luijten MAJ, van Muilekom MM, Teela L, Polderman TJC, Terwee CB, Zijlmans J, et al. The impact of lockdown during the COVID-19 pandemic on mental and social health of children and adolescents. Qual Life Res. 2021;30:2795–804. doi:10.1007/s11136-021-02861-x.

24. Howell CR, Gross HE, Reeve BB, DeWalt DA, Huang I-C. Known-groups validity of the Patient-Reported Outcomes Measurement Information System (PROMIS(®)) in adolescents and young adults with special healthcare needs. Qual Life Res. 2016;25:1815–23. doi:10.1007/s11136-016-1237-2.

25. Janssen LHC, Kullberg M-LJ, Verkuil B, van Zwieten N, Wever MCM, van Houtum LAEM, et al. Does the COVID-19 pandemic impact parents' and adolescents' well-being? An EMA-study on daily affect and parenting. PLoS One. 2020;15:e0240962. doi:10.1371/journal.pone.0240962.

26. Allgaier A-K, Pietsch K, Frühe B, Sigl-Glöckner J, Schulte-Körne G. Screening for depression in adolescents: validity of the patient health questionnaire in pediatric care. Depress Anxiety. 2012;29:906–13. doi:10.1002/da.21971.

27. Soest T von, Kozák M, Rodríguez-Cano R, Fluit DH, Cortés-García L, Ulset VS, et al. Adolescents' psychosocial well-being one year after the outbreak of the COVID-19 pandemic in Norway. Nat Hum Behav. 2022;6:217–28. doi:10.1038/s41562-021-01255-w.

28. Burdzovic Andreas J, Brunborg GS. Self-reported Mental and Physical Health Among Norwegian Adolescents Before and During the COVID-19 Pandemic. JAMA Netw Open. 2021;4:e2121934. doi:10.1001/jamanetworkopen.2021.21934.

29. Myhr A, Naper LR, Samarawickrema I, Vesterbekkmo RK. Impact of COVID-19 Pandemic Lockdown on Mental Well-Being of Norwegian Adolescents During the First Wave-Socioeconomic Position and Gender Differences. Front Public Health. 2021;9:717747. doi:10.3389/fpubh.2021.717747.

30. Hafstad GS, Sætren SS, Wentzel-Larsen T, Augusti E-M. Adolescents' symptoms of anxiety and depression before and during the Covid-19 outbreak - A prospective population-based study of teenagers in Norway. Lancet Reg Health Eur. 2021;5:100093. doi:10.1016/j.lanepe.2021.100093.

31. Kleppang AL, Hagquist C. The psychometric properties of the Hopkins Symptom Checklist-10: a Rasch analysis based on adolescent data from Norway. Fam Pract. 2016;33:740–5. doi:10.1093/fampra/cmw091.

32. Ertanir B, Kassis W, Garrote A. Longitudinal Changes in Swiss Adolescent's Mental Health Outcomes from before and during the COVID-19 Pandemic. Int J Environ Res Public Health 2021. doi:10.3390/ijerph182312734.

33. Borbás R, Fehlbaum LV, Dimanova P, Negri A, Arudchelvam J, Schnider CB, Raschle NM. Mental well-being during the first months of Covid-19 in adults and children: behavioral evidence and neural precursors. Sci Rep. 2021;11:17595. doi:10.1038/s41598-021-96852-0.

34. Walter R, Remschmidt H. Untersuchungen zur Reliabilität, Validität und Faktorenstruktur einer deutschsprachigen Version der Child Behavior Checklist. Zeitschrift für Klinische Psychologie und Psychotherapie. 1999;28:177–84. doi:10.1026//0084-5345.28.3.177.

35. Knowles G, Gayer-Anderson C, Turner A, Dorn L, Lam J, Davis S, et al. Covid-19, social restrictions, and mental distress among young people: a UK longitudinal, population-based study. J Child Psychol Psychiatry 2022. doi:10.1111/jcpp.13586.

36. Turner N, Joinson C, Peters TJ, Wiles N, Lewis G. Validity of the Short Mood and Feelings Questionnaire in late adolescence. Psychol Assess. 2014;26:752–62. doi:10.1037/a0036572.

37. Widnall E, Winstone L, Plackett R, Adams EA, Haworth CMA, Mars B, Kidger J. Impact of School and Peer Connectedness on Adolescent Mental Health and Well-Being Outcomes during the COVID-19 Pandemic: A Longitudinal Panel Survey. Int J Environ Res Public Health 2022. doi:10.3390/ijerph19116768.

38. White D, Leach C, Sims R, Atkinson M, Cottrell D. Validation of the Hospital Anxiety and Depression Scale for use with adolescents. Br J Psychiatry. 1999;175:452–4. doi:10.1192/bjp.175.5.452.

39. Wright N, Hill J, Sharp H, Pickles A. Interplay between long-term vulnerability and new risk: Young adolescent and maternal mental health immediately before and during the COVID-19 pandemic. JCPP Adv. 2021;1:e12008. doi:10.1111/jcv2.12008.

40. Rhew IC, Simpson K, Tracy M, Lymp J, McCauley E, Tsuang D, Stoep AV. Criterion validity of the Short Mood and Feelings Questionnaire and one- and two-item depression screens in young adolescents. Child Adolesc Psychiatry Ment Health. 2010;4:8. doi:10.1186/1753-2000-4-8.

41. Bignardi G, Dalmaijer ES, Anwyl-Irvine AL, Smith TA, Siugzdaite R, Uh S, Astle DE. Longitudinal increases in childhood depression symptoms during the COVID-19 lockdown. Arch Dis Child 2020. doi:10.1136/archdischild-2020-320372.

42. Chinn S. A simple method for converting an odds ratio to effect size for use in meta-analysis. Statist. Med. 2000;19:3127–31. doi:10.1002/1097-0258(20001130)19:22%3C3127::AID-SIM784%3E3.0.CO;2-M.

43. Naumann E, den Driesch E von, Schumann A, Thönnissen C. Anstieg depressiver Symptome bei Jugendlichen und jungen Erwachsenen während des ersten Lockdowns in Deutschland : Ergebnisse des Beziehungs- und Familienpanels pairfam. [Increase of depressive symptoms among adolescents during the first COVID-19 lockdown in Germany : Results from the German family panel pairfam]. Bundesgesundheitsblatt Gesundheitsforschung Gesundheitsschutz. 2021;64:1533–40. doi:10.1007/s00103-021-03451-5.

44. McGuinness LA, Higgins JPT. Risk-of-bias VISualization (robvis): An R package and Shiny web app for visualizing risk-of-bias assessments. Res Synth Methods. 2021;12:55–61. doi:10.1002/jrsm.1411.

45. Hale T, Angrist N, Goldszmidt R, Kira B, Petherick A, Phillips T, et al. A global panel database of pandemic policies (Oxford COVID-19 Government Response Tracker). Nat Hum Behav. 2021;5:529–38. doi:10.1038/s41562-021-01079-8.

46. COVIDSurg Collaborative. Effect of COVID-19 pandemic lockdowns on planned cancer surgery for 15 tumour types in 61 countries: an international, prospective, cohort study. Lancet Oncol. 2021;22:1507–17. doi:10.1016/S1470-2045(21)00493-9.

47. Phillips T. Codebook for the Oxford Covid-19 Government Response Tracker: Codebook version 4.0. 2022. https://github.com/OxCGRT/covid-policy-tracker/blob/master/documentation/codebook.md. Accessed 21 Aug 2022.

1. Due to the lack of randomization, studies should be typically be judged as “Serious RoB” within the item “Bias due to confounding” [1]. However, randomization of the exposure and comparison group automatically exists in this study design because pre-pandemic and during pandemic estimates were compared. [↑](#footnote-ref-1)
2. It must be considered that the influence factors on the pandemic and/or on depression and anxiety are very heterogeneous and depends on various factors (e.g., time point in pandemic). So, there is possibility of residual unmeasured (and unidentified) confounding. [↑](#footnote-ref-2)
3. Due to the lack of randomization, studies maybe be judged as “Serious RoB” within the item “Bias in selection of participants into study” [1]. [↑](#footnote-ref-3)
4. European population is automatically affected by the COVID-19 pandemic as the virus achieved Europe in March 2020. [↑](#footnote-ref-4)
5. The COVID-19 pandemic exposure classification was not further subdivided (e.g., into school closures) to reflect the full radius of effects of the COVID-19 pandemic. [↑](#footnote-ref-5)
6. Departures from the intended exposure (COVID-19 pandemic) are not possible. [↑](#footnote-ref-6)
